# Fecal microbiota transfer between young and aged mice reverses hallmarks of the aging gut, eye, and brain

**DOI:** 10.1186/s40168-022-01243-w

**Published:** 2022-04-29

**Authors:** Aimée Parker, Stefano Romano, Rebecca Ansorge, Asmaa Aboelnour, Gwenaelle Le Gall, George M. Savva, Matthew G. Pontifex, Andrea Telatin, David Baker, Emily Jones, David Vauzour, Steven Rudder, L. Ashley Blackshaw, Glen Jeffery, Simon R. Carding

**Affiliations:** 1 Gut Microbes and Health Research Programme, Quadram Institute, Norwich, NR4 7UQ UK; 2 Institute of Ophthalmology, University College London, London, EC1V 9EL UK; 3 Norwich Medical School, University of East Anglia, Norwich, NR4 7TJ UK

**Keywords:** Aging, Microbiota, Gut–brain axis, Gut–retina, Intestine, Fecal microbiota transplantation, Leaky gut, Inflammaging

## Abstract

**Background:**

Altered intestinal microbiota composition in later life is associated with inflammaging, declining tissue function, and increased susceptibility to age-associated chronic diseases, including neurodegenerative dementias. Here, we tested the hypothesis that manipulating the intestinal microbiota influences the development of major comorbidities associated with aging and, in particular, inflammation affecting the brain and retina.

**Methods:**

Using fecal microbiota transplantation, we exchanged the intestinal microbiota of young (3 months), old (18 months), and aged (24 months) mice. Whole metagenomic shotgun sequencing and metabolomics were used to develop a custom analysis workflow, to analyze the changes in gut microbiota composition and metabolic potential. Effects of age and microbiota transfer on the gut barrier, retina, and brain were assessed using protein assays, immunohistology, and behavioral testing.

**Results:**

We show that microbiota composition profiles and key species enriched in young or aged mice are successfully transferred by FMT between young and aged mice and that FMT modulates resulting metabolic pathway profiles. The transfer of aged donor microbiota into young mice accelerates age-associated central nervous system (CNS) inflammation, retinal inflammation, and cytokine signaling and promotes loss of key functional protein in the eye, effects which are coincident with increased intestinal barrier permeability. Conversely, these detrimental effects can be reversed by the transfer of young donor microbiota.

**Conclusions:**

These findings demonstrate that the aging gut microbiota drives detrimental changes in the gut–brain and gut–retina axes suggesting that microbial modulation may be of therapeutic benefit in preventing inflammation-related tissue decline in later life.

Video abstract

**Graphical abstract:**

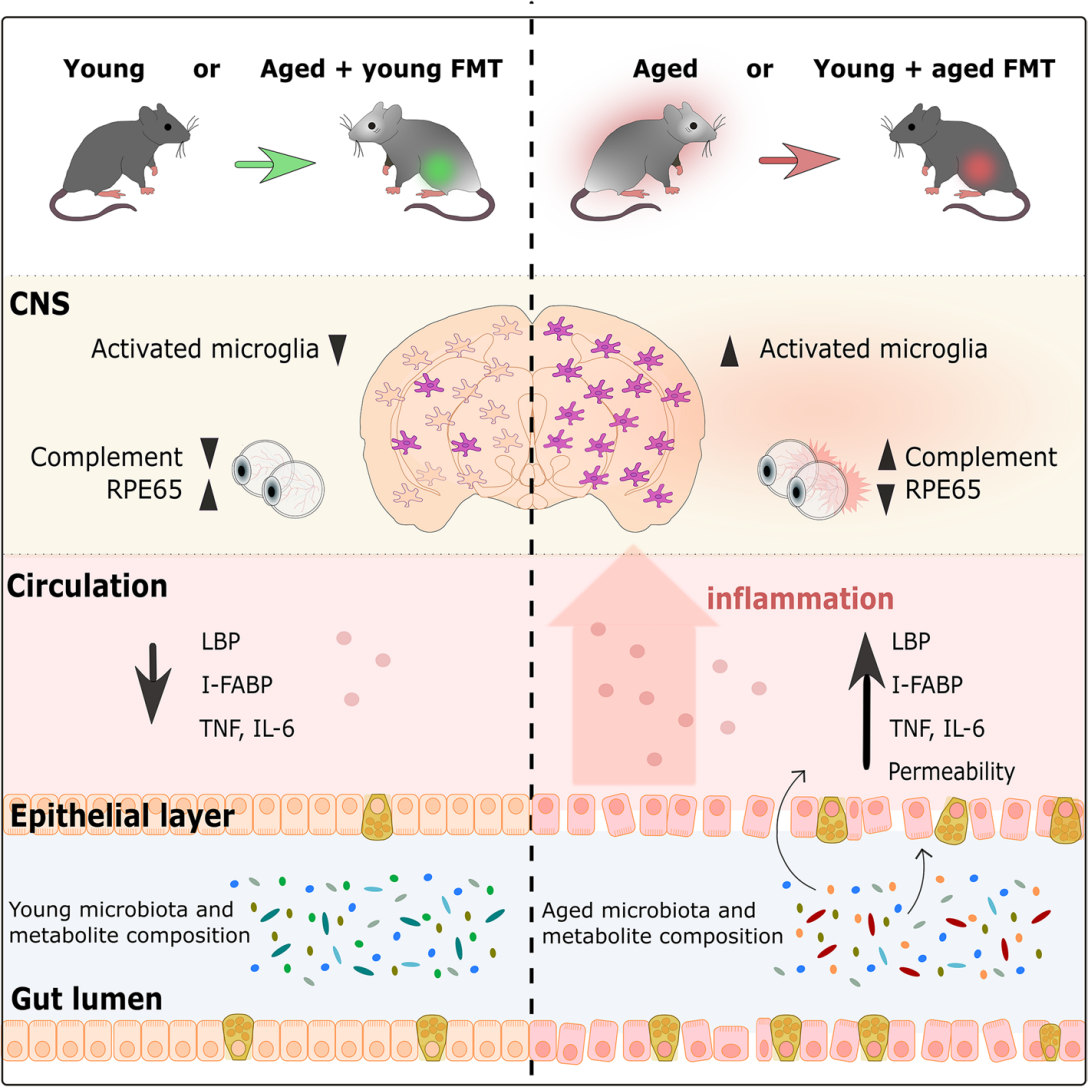

**Supplementary Information:**

The online version contains supplementary material available at 10.1186/s40168-022-01243-w.

## Background

Aging is characterized by declining cell, tissue, and organ function and increasing susceptibility to chronic and debilitating diseases. Particularly vulnerable are tissues of the central nervous system (CNS) and the eye, which have high sensitivity to metabolic dysregulation, and the gastrointestinal (GI) epithelial barrier, which must maintain rapid cellular turnover and barrier integrity while faced with constant exposure to environmental insults [14, 85].

Comprising bacteria, viruses, fungi, protozoa, and archaea, the intestinal microbiota contributes to the life-long health of the host, playing key roles in the development and maintenance of the host immune system and the intestinal epithelial barrier [111]. During aging, the intestinal microbiota undergoes changes in the community structure and function, with samples from elderly individuals showing changes in the proportions of different taxa, increased inter-individual variability, and altered diversity. These changes can adversely affect the host metabolism and immunity [25–27, 40, 64, 86, 110, 112, 118] and are associated with the development of cardiovascular, autoimmune, metabolic, and neurodegenerative disorders [17, 90, 122, 132].

Microbiota transfer studies in fly and fish models of aging suggest a direct role of the microbiota in regulating lifespan and age-associated disease pathogenesis. Young flies fed homogenates from aged flies had significantly decreased lifespan and increased incidence of intestinal barrier dysfunction, compared with flies fed homogenates from young animals [27]. In the short-lived African killifish, the transfer of young donor microbiota to aged individuals ameliorated behavioral decline and extended lifespan [110]. Similar beneficial effects have been reported in aged mice receiving microbiota from young mice to extend life span [8, 61] or halt the functional decline in the mucosal immune system [112].

One of the major organ systems adversely affected by aging and inflammaging is the brain, with various lines of investigation providing evidence for a “microbiota–gut–brain axis” via which gut microbes can influence brain health via various mechanisms. These include complex and interlinked hormonal signaling, immune signaling, and neural signaling networks in the host, which can be modulated by gut microbial products and which can in turn modify the gut microbiota composition and function [90]. Sequencing studies showing associations between particular gut microbiota profiles and various diseases in humans, along with studies modifying the gut bacterial composition in animal models, by probiotic administration or microbiota transfer, implicate the gut microbiota as a key regulator of systemic homeostasis, including neuroinflammation and behavioral disorders [16, 106]. One possible scenario is that changes in the gut microbiota composition or function trigger or, contribute to, the decline of normal gut epithelial barrier function, which in turn drives systemic chronic inflammation that has detrimental effects on various tissues around the body. In aging, detrimental changes in the diversity, composition, or function of the gut microbiota could therefore promote neuroinflammation and functional decline in the CNS.

In the aged GI tract, compromised epithelial and mucosal cell turnover, integrity, and function [32, 36, 76, 83] can lead to increased epithelial barrier permeability [19, 75, 118, 120], with the increased possibility of luminal microbial and dietary antigens gaining access to the circulation. This, together with declining immune cell function and immunosenescence, promotes a state of elevated systemic inflammation termed “inflammaging” [39, 40, 118], contributing to the functional decline in the intestine and CNS in old age [10, 13, 48].

In the brain, microglial cells play multiple roles in regulating inflammatory responses and neural functioning and are now considered central to the pathology of neurodegenerative diseases [48]. Studies using germ-free mice, antibiotic treatment, and selective microbial colonization suggest that the intestinal microbiota can regulate microglial maturation and function in the enteric and central nervous systems [37, 54]. Attenuation of pathology following microbial modulation, in transgenic mouse models of Alzheimer’s disease [57, 115], also supports a role for the microbiota in inflammaging in the brain.

As an extension of the CNS, the eye is exposed to multiple stressors, including oxidative stress-inducing UV light and age-related immune dysregulation, and is vulnerable to energy insufficiency resulting from altered circulation and metabolism in old age [72]. With advancing age, the outer retina of the eye displays elevated inflammation and widespread deposition of extracellular debris, including amyloid species [55, 88]. Age-related macular degeneration (AMD) is associated with chronic inflammation [29, 65] and altered fecal microbial metabolite profiles [100]. The altered microbial composition has also been associated with neovascularization and immune cell activation in retinal disease models [4, 50, 99].

To investigate the effects of age-associated microbiota composition on inflammaging and its impact on the gut, brain, and retina, we used young (3 months), old (18 months), and aged (24 months) mice. We compared mice receiving microbiota from a different age group (heterochronic) fecal microbiota transplant (FMT) with mice receiving microbiota from the same age group (coeval FMT). Control animals received antibiotics only (antibiotic treatment controls) or PBS only (gavage/procedural controls). We compared the systemic and tissue-specific biomarkers of inflammation and function in the brain and retina, intestinal epithelial barrier integrity, circulating inflammatory markers, and behavioral impacts. To avoid confounding impacts of vendor influences on the microbiota of different age groups, our study used mice of three different ages from the same colony, which were all bred and maintained in the same environment for their entire lifespan. Importantly, we also accounted for any potential cage grouping or housing effects in our analyses.

We show that FMT from between young and aged mice replaced the recipient microbiota composition with a composition resembling the donor, enriched for particular bacterial species, and altered the metabolic potential of the resulting gut microbial composition. Our results demonstrate that the aging microbiota mediates detrimental changes in microglial activation in the brain, and inflammation and functional protein expression in the eye in parallel with altered intestinal barrier permeability. Detrimental effects were accelerated upon transfer of aged donor microbiota into young mice, but conversely, age-associated inflammatory changes were reversed in aged mice by transfer of young donor microbiota.

## Methods

### Experimental model details

#### Mice

All mice were bred and maintained under specific pathogen-free (SPF) conditions at the University of East Anglia (UEA) Disease Modelling Unit Facility, housed in individually ventilated cages with three to five mice per cage. Mice were fed a standard chow diet provided with water ad libitum and maintained under a 12-h light:12-h dark cycle. Male C57BL/6J mice aged 3, 18, or 24 months were used in this study and were all bred and housed in the same room. All experiments were approved by the UEA Animal Welfare and Ethical Review Body

### Experimental procedures

#### Microbiota transfer

**Fig. 1:**
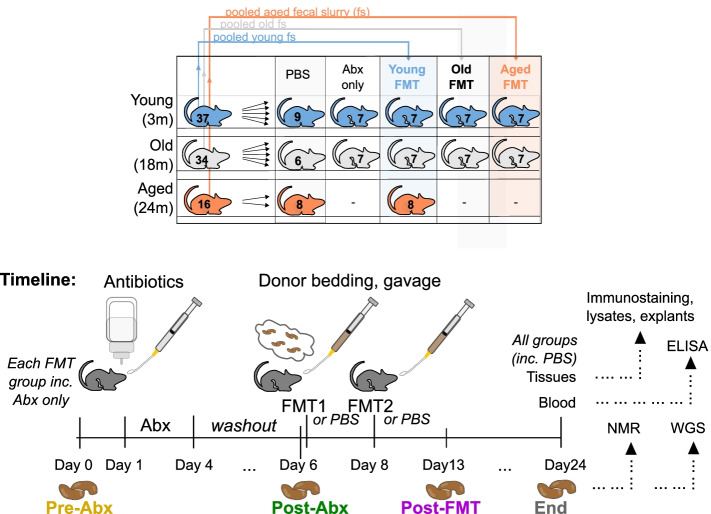
Experimental Overview. **A** Mouse grouping and **B** microbiota transfer timeline overview. Young (3 m, blue), old (18 m, white), or aged (24 m, orange) SPF C57BL/6 males were randomly allocated to experimental cages by age group 2 weeks before the start of the experiment. Existing microbiota were depleted by a 3-day antibiotic cocktail (Abx; oral gavage and drinking water). Post-antibiotic washout, recipients were rehoused in donor cages containing soiled bedding and fecal pellets and were twice delivered donor microbiota by oral gavage of fecal slurry preparation from 3-, 18-, or 24-month-old donors. Antibiotic-only groups were returned to their home cage and received no FMT

Groups of young (3 months), old (18 months), or aged (24 months) male mice were randomly re-assigned to experimental cages 2 weeks prior to the start of the experiment. Groups of seven mice (see Fig. 1A), designated for antibiotic/fecal microbiota transfer (FMT) interventions, were sub-housed as groups of three or four due to cage occupancy restrictions. PBS-only groups were also sub-housed in groups of three, four, or five as necessary. Microbiota modulation can be induced by simply co-housing heterochronic groups of mice [112]; however, we chose to also deplete host gut microbiota by prior antibiotic cocktail administration, to maximize potential engraftment of the donor microbiota. Existing microbiota were depleted by delivery of 3-day broad-spectrum antibiotic cocktail regime by combined oral gavage of unpalatable antibiotics (100 μL of vancomycin, 5 mg/mL; metronidazole, 10 mg/mL) while others were able to be provided in drinking water (ampicillin 1 g/L, neomycin 0.5 g/L). Post-antibiotic washout, recipients were rehoused in heterochronic donor cages containing soiled bedding and fecal pellets and were twice delivered heterochronic donor microbiota 72 h apart by oral gavage of fecal slurry preparation. Pre-Abx samples and FMT slurry are equivalent in that they were prepared from pellets collected at the Pre-Abx time point, with half the sample sent to sequence and half used to prepare the slurry. Fecal slurries were prepared from Pre-Abx donor mice by pooling fecal pellet material from two mice per cage (chosen randomly within each cage), for each age group. Thus, young donor microbiota was a pool representing 20 of 37 mice housed across 10 cages; old donor microbiota was also a pool representing 20 of 34 mice housed across 10 cages. For the aged pool, pellets were included from all 16 mice across four cages. Each mouse received 100 μL fecal slurry preparation at each gavage. At pellet collection, mice were separated into individual sterile boxes and two pellets collected before returning to the cage. The Pre-Abx samples represented in Fig. 5 show the results of sequencing one fecal pellet of the two from each individual mouse. The fecal slurry was produced by combining the second pellets from a random subset of these same mice. The pellets sequenced and represented as the “baseline” composition for each age group in our analyses therefore representing the composition from the mice whose same pellets were delivered as donor pellets the FMT. Control groups for 3-month-old and 18-month-old mice receiving heterochronic transfer included gavage with PBS only and antibiotic treatment/PBS gavage only, and groups receiving coeval (donor pool of the same age group) soiled cages and gavage. Due to the limited availability of 24-month aged mice, only heterochronic FMT and no-transfer controls were possible for this age group. Transfer of soiled bedding was repeated every 3 days. Fecal pellets were collected for sequencing at “Pre-Abx,” “Post-Abx,” “Post-FMT” (7 days post-transfer), and “end” (18 days post-transfer) time points (see Fig. 1B). Pellets were collected from all mice at the four time points, after briefly separating into single-occupancy sterile boxes, and collected using sterile picks and DNA/RNA-free sterile microtubes, which were then stored at − 80 °C until DNA extraction. NMR analysis used pellet samples at the “end” time point. Blood and tissues were also collected at the end of the experiment. Fecal pellets, blood, and tissue collections and FMT interventions were all performed at the same time of day across all cages to account for circadian rhythm variability in feeding and microbiota/metabolite composition.

#### Metagenomic library preparation and sequencing

Genomic DNA from fecal samples was isolated using the FastDNA SPIN Kit for Soil (MP Biomedicals), quantified using the Qubit dsDNA BR Assay Kit and Qubit 3.0 fluorimeter (Invitrogen) and normalized to 5 ng/μL. A miniaturized reaction was set up using the Illumina Nextera DNA Flex Library Prep Kit (Illumina). 0.5 μL Tagmentation Buffer 1 (TB1) was mixed with 0.5 μL bead-linked transposomes (BLT) and 4.0 μL PCR-grade water in a master mix and 5 μL added to a chilled 96-well plate. Two microliters of normalized DNA (10 ng total) was pipette mixed with the 5 μL of the tagmentation mix and heated to 55 °C for 15 min. PCR master mix (Kap2G Robust PCR kit, Merck) was combined with P7 and P5 Nextera XT Index Kit v2 index primers (Illumina) and tagmentation mix and amplified by PCR. Libraries were quantified using the Quant–iT dsDNA Assay high-sensitivity kit (Invitrogen) and run on a GloMax® Explorer Multimode Microplate Reader (Promega). Libraries were pooled in equal quantities following quantification. The final pool was double-SPRI size selected between 0.5 and 0.7× bead volumes using KAPA Pure Beads (Roche), quantified on a Qubit 3.0 fluorimeter and run on a D5000 ScreenTape system (Agilent) using the Agilent Tapestation 4200 to calculate the final library pool molarity. “Kitom” controls (water and PBS controls processed through the same DNA extraction process as all samples) were included along with the DNA extraction process, but these control samples then failed at the library preparation step due to very low available input material. This suggests there was no or insufficient DNA contamination resulting from the kit to be of significance to the subsequent analysis of the metagenomic data. Libraries were sequenced by NGS Illumina sequencing to a depth of 10 Gbp/sample by Novogene, UK. Per-sample read numbers are shown in Supplementary Fig. S6.

#### Metagenomic sequence data processing

Raw sequence reads were trimmed to a quality of phred 20, and adapters and PhiX Illumina standards removed using BBDuk v38.76 (sourceforge.net/projects/bbmap/) [20]. Human read contamination was removed using BBMap v38.76 and a masked reference of the hg19 dataset released by Brian Bushnell (https://zenodo.org/record/1208052#.X9DiS6r7SHw). Reads mapping to the mouse genome (*Mus musculus* GRCm38) were removed using BBMap v38.76 (parameters: *minid=0.95*; *maxindel=3*; *bwr=0.16*; *bw=12*; *quickmatch*; *fast; minhits=2*). Taxonomic profiling was performed on the filtered reads using MetaPhlAn (v3.0.0.0–alpha) [12, 107], including estimation of the unknown fraction (parameter: --*unknown_estimation*) with the database mpa_v30_CHOCOPhlAn_201901. Functional read profiling was performed using HUMAnN3 (v3.0.0.alpha.1) [12, 41] including MetaPhlAn, DIAMOND v0.9.24 [18], and the databases uniref90 (v201901) [116] and mpa_v30_CHOCOPhlAn_201901.

#### Alpha and beta diversity analyses

Species relative abundances obtained from MetaPhlAn (v3.0.0.0–alpha) were imported into R [92] and manipulated using the *phyloseq* package v1.30.0 [79]. The number of observed species was calculated using the *richness* function in the R package *microbiome* v1.8.0 [63]. Statistical differences in the number of observed species across the different groups were tested using linear mixed models (*lmm*) with cage as a random effect (*observed species ~ time point + (1|cage)*). Models were built using the *lmer* function in *lme4* v1.1.21 [9] R packages, and pairwise contrasts were extracted using the *emmeans* v1.4.5 [69] function with default parameters.

Beta-diversity analyses were initially performed using Bray–Curtis (BC) and Jensen–Shannon divergence (JSD) indices on all samples obtained throughout the experiment. Ordinations were built using PCoA, and negative eigenvalues were set to 0. While the BC index allowed better visualization of the data, JSD explained a greater proportion of data variance and thus was used for further testing. To verify the divergence between time points and simultaneously account for cage effect, we used a JSD distance matrix and created a dbRDA, conditioning the data for cage and constraining per time point. dbRDA was performed using the “CAP” option in the *ordinate* function in *phyloseq*. The resulting model was used to perform a permutational ANOVA, function *anova.cc* in the *vegan* v2.5.6 [89] R package, limiting the permutation within cages (permutations = how (blocks = cage, nperm = 10,000)). Pairwise PERMANOVA [5] were carried out between time points using *pairwiseadonis* v0.0.1, limiting the 10,000 permutations within cages and using Benjamini–Hochberg (BH) for *P* value correction. Additional ordinations were done for samples obtained at the beginning of the experiment (Pre-Abx) and for samples of young mice receiving either young or aged FMT and old mice receiving young FMT (Post-FMT). Ordinations used JSD as indicated above. Pairwise PERMANOVA was used to assess microbiota divergence between groups using BH *P* value correction. For comparison between pre- and post-FMT, permutation was restricted within cages (*strata* = cage). Since recipients were confounded within cages, free permutation was allowed in the pairwise comparisons for the Pre-Abx dataset. To infer species that were major drivers of sample divergence in the beta diversity analyses, species abundance was correlated to the ordinations using the *envfit* function in the R *vegan* package allowing 10,000 permutations. *P* values were corrected using false discovery rate (fdr), and only taxa showing *P* < .005 and the strongest degree of variation along the axis (≥|0.43|) were selected. All species showing *P* < .05 were instead plotted on the ordination including only samples referring to the Pre-Abx time point. Finally, *procrustes* tests were used to estimate the correlation between ordinations obtained for single time points. PCoA ordinations obtained for the shared samples across time points were used to run a *protest* in the R *vegan* package allowing 10,000 permutations.

#### Differential abundance analyses

Differential abundance (DA) analyses were performed using linear mixed models (*lmm*) for family and species, and the unstratified pathways were obtained from MetaPhlAn (v3.0.0.0–alpha). For taxonomy, *lmm* were built using samples from Pre-Abx, Post-FMT, and at the end of the experiment. To accommodate the different data distributions of each species, we built *lmm* and tested multiple transformations, building *lmm* for each, using centered log ratios (function codaSeq.clr in the R package CodaSeq v0.99.6) [44], log2, ArcSin, and logit, all performed after adding a constant to 0 values, and log2Zero, where Na/Inf, derived from the log transformation, were converted to 0. Residuals were manually inspected. We selected all *lmm*-transformation combinations that had roughly random and normally distributed residuals. We then selected all species that had significant *P*-values for each contrast of interest (e.g., Pre-Abx vs. Post-FMT). Finally, to homogenize the results, we reported the CLR transformed data for the final figures as presented. For the metabolic pathway analysis, we again tested multiple transformations, and *lmm* were also built on non-transformed data as this condition produced residuals roughly randomly and normally distributed. Differential abundance analysis was performed after filtering the datasets retaining all species with relative abundance > 0 in ≥ 5% of samples and all genera and families with relative abundance > 0 in ≥ 15% of samples. Only pathways present in all samples of at least one time point across the groups were considered (e.g., all samples of young receiving old FMT at the beginning of the experiment). *lmm* were built as follows: taxa/path ~ receiver * donor * time point + (1|cage/mice). Specific contrasts were selected using the *emmeans* function (*specs = ~ receiver*:*donor*:*time point*) without *P* value correction. After obtaining all contrasts of interest for all taxa/pathways, all *P* values were corrected using fdr. Finally, to test the differential abundance of species Pre-Abx between the age groups, we selected only the samples relative to this time point and built the *lmm*: *taxa/path ~ receiver + (1|cage)* using the approach specified above and considering only species with a relative abundance > 0 in ≥ 10% of samples. The extraction of the contrasts and *P* value correction were performed as above.

#### Analysis of fecal metabolite concentrations by nuclear magnetic resonance (NMR)

Fecal samples were homogenized in 1:12 w/v of saline phosphate buffer (1.9 mM Na_2_HPO_4_, 8.1 mM NaH_2_PO_4_, 150 mM NaCl) with 1 mM sodium 3-trimethysilyl-propionate-d4 (TSP-d4) mixed 1:1 v/v with deuterated water (Merck) using ceramic bead beating (Lysing Matrix E, MP Biomedicals), followed by centrifugation at 15,000*g*, 5 min, and filtration of supernatants at 0.2 μm. ^1^H NMR spectra were recorded using a 600-MHz Bruker Avance spectrometer fitted with a 5-mm TCI proton-optimized triple resonance NMR inverse cryoprobe and autosampler (Bruker). Sample temperature was controlled at 300 K. Spectra were transformed with a 0.3-Hz line broadening and zero filling, manually phased, baseline corrected, and referenced by setting the TSP–d4 signal to 0 ppm. Metabolites were identified by comparison with existing literature, an in-house database of mouse fecal metabolite spectra, the Human Metabolome Database (http://www.hmdb.ca/), and by use of 2-dimensional NMR ^1^H–^1^H correlation spectroscopy, ^1^H–^13^C heteronuclear single quantum correlation, and ^1^H–^13^C heteronuclear multiple bond correlation spectroscopy [67, 68, 121]. Concentrations were calculated using Chenomx NMR Suite 7.0, centered (CLR), and log-transformed.

#### Metabolome data analysis

Only metabolites present in > 10% of samples or at least three individual animals within one experimental group were analyzed. Data were log2-transformed after adding a constant (0.001) to 0 values and were centered using the function *scale* in R. After calculating, the Euclidean distance data were plotted using PCoA. One sample from the aged Pre-Abx group was removed as it clearly represented an outlier in the ordination. A dbRDA model was then built constraining the data for mouse groups and conditioning the data for cage. Permutational ANOVA on the model (*anova.cca*) was then performed restricting the 10,000 permutations within cages. Similarly, pairwise PERMANOVA on the Euclidean distance matrix was performed using 10,000 permutations restricted within cages and correcting *P* values using FDR. Sparse PLS–DA was performed using the function *splsda* in the R package *mixOmics* v6.10.9 [96]. Model performance and component and variable selections were tested using a five-fold validation and 75 repetitions with the functions *perf* and *tune.splsda* within the *mixOmics* package. Differential abundance analysis was initially performed using lmm as specified above on scaled values. Since models showed a poor fit (residuals not randomly or normally distributed), we performed the analysis using a Kruskal–Wallis non-parametric test, blocking the dataset for cages. For this purpose, we used the *kruskal_test* function in the R package *coin* v1.3.1 [51]. *P* values were corrected using FDR.

#### Quantification of serum/intestinal proteins

Blood samples collected at the end of the experiment were allowed to coagulate for 30 min at room temperature, before centrifugation at 1500*g* for 10 min. The serums were removed and stored at − 80 °C until further use. Serum I-FABP, lipopolysaccharide-binding protein (LBP), and IL-6 were measured using specific ELISAs or as part of a mouse magnetic Luminex panel (detailed in resources file). Lower detection limits of specific ELISAs: I-FABP 0.15 ng/μL, LBP 0.8 ng/mL, IL-6 24.6 pg/mL, and TNF 0.29 pg/mL. Intestinal lysates were prepared from duodenal samples. Tissue samples (50 mg) were homogenized in CelLytic MT (Merck) with added complete protease inhibitor cocktail (Roche) using a FastPrep Bead Beater and Lysing Matrix D tubes (MP Biomedicals) and centrifuged 15 min at 17,000*g*, 4 °C. Duodenal lysate tumor necrosis factor (TNF) was measured using specific ELISA (detailed in key resources). Total protein concentrations in the serum and lysates were normalized by Bradford assay (BCA protein assay) prior to use in ELISA/Luminex. Four-parameter logistic regression fits for a 6-point standard curve, interpolation of sample concentrations (triplicate serum samples from *n* = 5 mice per group).

#### Quantification of retinal proteins

Levels of ~ 40 target cytokines were assayed using a Proteome Profiler Mouse Cytokine Array (BioTechne), using retinal lysates from aged mice young donor microbiota vs. aged donor microbiota (*n* = 8 mice/group). Lysates were generated for whole individual retinae by lysis in PBS + complete protease inhibitors (SigmaFast), sonication to disrupt cell membranes, freeze-thaw with detergent (Triton X-100), and clarification by centrifugation (10 min at 17,000*g*, 4 °C, before storage at − 80 °C. Pooled lysates (*n* = 8 retinae/group) were incubated with antibody panel-coated membranes overnight at 4 °C, and the rest of the procedure was performed as per the manufacturer’s instructions. Chemiluminescent detection of membranes used a ChemiDoc XRS system (BioRad), and images were exported to dedicated analysis software (ImageLab, BioTechne) for quantification. Following background removal and control membrane (PBS, no lysate) removal, pixel intensities were converted to % change in aged + young FMT vs. aged + aged FMT membranes for each target spot. Nine targets which fell below the threshold of detection or below the control membrane intensity are not displayed.

#### Retinal immunostaining and quantification

Eyes were fixed 20 min in 4% paraformaldehyde (PFA) before cryopreservation and embedding in OCT compound (Agar Scientific). Cryosections were prepared at 5 μm and stained with goat polyclonal antiserum against complement C3 (MP Biomedicals) or mouse monoclonal antibody to RPE65 (Merck Millipore) and secondary antibodies donkey anti-goat A488, donkey anti-mouse A568 (Invitrogen) nuclei stained with DAPI (Merck). Images were collected using an epifluorescence widefield microscope. The average pixel intensity of a 1280-μm area of the interface between the RPE and Bruch’s membrane interface was measured in Adobe Photoshop CS5. Five mice (retinae) were analyzed per age per group.

#### Brain Iba-1 cell staining and counts

**Fig. 2:**
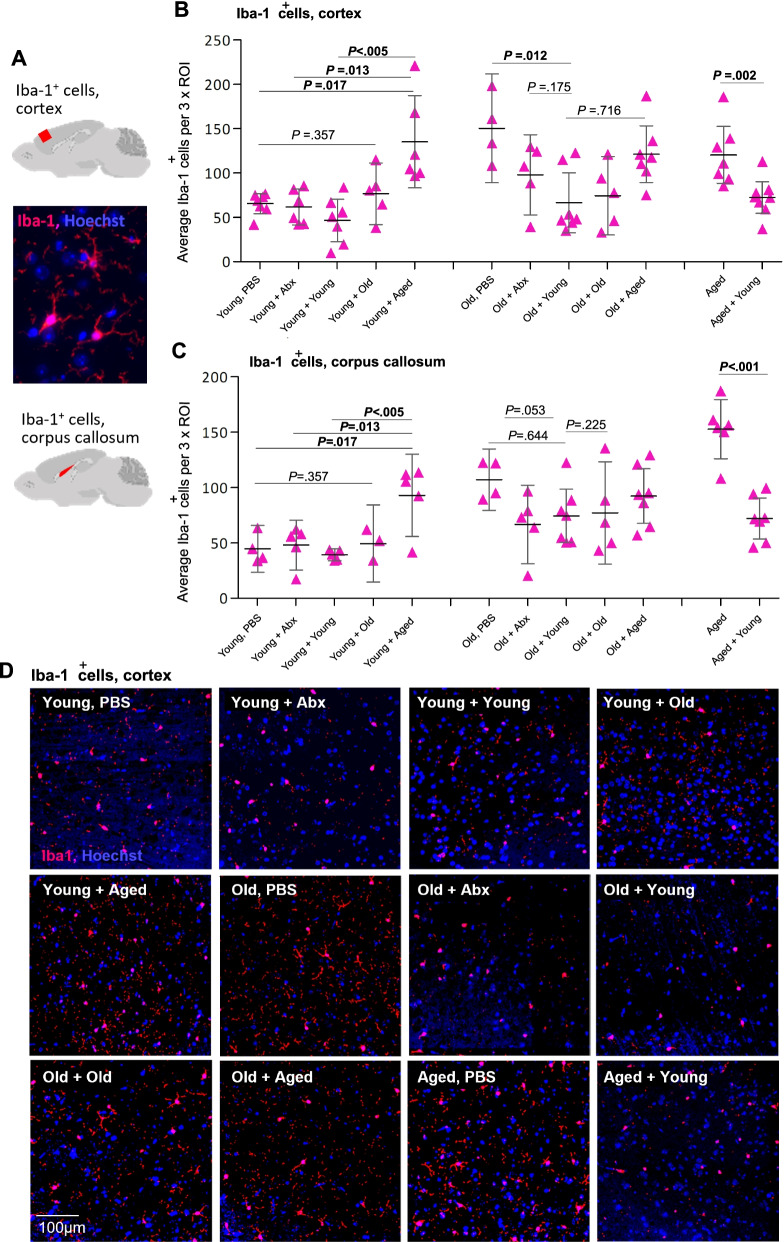
Inflammatory (Iba-1^+^) microglia density in cortex and corpus callosum is regulated by the intestinal microbiota. **A** Iba-1+ microglia were identified by immunostaining (red), nuclei counterstained with Hoechst (blue), and quantified in the cortex and corpus callosum (highlighted in cartoons) of sagittal mouse brain sections from all groups of mice. **B** Quantification of Iba-1+ cells in the cortex, average count across 3 regions of interest (ROI) from each of 4–7 mice per group from all groups. **C** Quantification of Iba-1+ cells in the corpus callosum, an average of 3 regions of interest from each of 4–7 mice per group from all groups. Statistical analysis between the groups of interest by Welch’s *t* test; error bars denote 95% CI. Significant values are in bold. **D** Representative immunostaining of Iba-1+ cells in the cortex of young, old, and aged mice, either treated with PBS only, treated with antibiotics only, or with antibiotics followed by FMT from young, old, or aged donors (see also Fig. S1)

Brains were formalin-fixed, paraffin-embedded, and sagittal sections were prepared at 4 μm. Iba1-positive microglia were detected by immunostaining with rabbit monoclonal antibody against Iba-1 (1:200, Abcam), donkey anti-rabbit Alexa-594 secondary antibody (Invitrogen), and Hoescht as the nuclear stain. Images were captured by Zeiss LSM880 confocal microscope and the Zen 2010 software. The choice of the cortex and corpus callosum for the analysis was based primarily on our initial observations that Iba-1 densities appeared different between pre-and post-treatment groups in these areas. Cell counts were performed in FIJI/ImageJ [101, 105]. Using images of the Iba-1 channel (A594) only, background random pixel noise was reduced using the “Despeckle” function. The threshold was then set the same for all images and images made binary. Triplicate area-matched circular regions of interest (ROIs) were placed to encompass as much of the tissue section as possible. The ROIs were then re-used for all images so that identically sized areas were captured for each image. The “Analyze Particles” function was then used to count particles within the three regions, and the results were exported to Excel. Three regions of interest per image, per section, per mouse, and per brain region were analyzed, *n* = 5–7 mice per group. Where any groups have fewer than 7 points represented in Fig. 2, this reflects the exclusion of any samples which had gaps/folds or autofluorescent debris within one or more ROIs.

### Behavioral tests

To assess the impact of FMT upon cognition and behavior, Y-maze spontaneous alternation test and novel object recognition (NOR) were performed as previously described [91]. Aged mice were tested pre- and post-FMT with young donor fecal microbiota (*n* = 10) and compared with aged control mice receiving aged donor microbiota (*n* = 10), and with young baseline mice (*n* = 11). Briefly, mice were placed into the Y-maze and allowed to explore freely for 5 min while tracking software recorded zone transitioning and locomotor activity (Smart 3.0 tracking software, Panlab, Kent, UK). Spontaneous alternation was calculated using the following formula: spontaneous alternation = (number of alternations/total arm entries − 2) × 100. Novel object recognition (NOR) was conducted as follows: on day 1, mice were habituated to the empty maze, being allowed to move freely for 10 min. On day 2, mice were conditioned to a single object for a 10-min period. On day 3, mice were placed into the same experimental area in the presence of two identical objects for 15 min, after which they were returned to their respective cages and an inter-trial interval of 1 h was observed. One familiar object was replaced with a novel object. Mice were placed back within the testing area for a final 10 min. Videos were analyzed for a 5-min period, after which if total object exploration time failed to reach an accumulative 10 s, the analysis continued until the 10 s was met. Animals not achieving 10 s were excluded from the analysis. Similarly, animals not achieving a cumulative 10 s with the familiar object were excluded. The discrimination index was calculated as follows: DI = (TN − TF)/(TN + TF), where TN is the time spent exploring the novel object and TF is the time spent exploring the familiar object.

### Statistical analysis

Statistical analysis of microbial diversity and pathway differential abundance were performed as described in detail above in the “Alpha and beta diversity analyses,” Metabolome data analysis,” and “Differential abundance analyses” sections. Other standard statistical tests (as stated in figure legends) were performed in *Jamovi*, v1.1.9.0 [117] using *P* values < .05 as the cutoff for significance. Typically, pairwise group comparisons were made within age groups between PBS-only and FMT-treated mice and for PBS-only mice between the age groups.

## Results

Using FMT, we exchanged the fecal microbiota of the “young” (3-month-old), “old” (18-month-old), and “aged” (24-month-old) groups of C57BL/6 mice (referred to throughout as “young,” “old,” and “aged” mice, respectively) The experimental design and timeline are summarized in Fig. 1. Tissues and blood were collected at the end of the experiment (Fig. 1, “end”) to determine the impact of heterochronic FMT on the susceptibility of key tissues and organs to inflammaging, in particular, the brain, retina, and intestinal epithelium.

### The intestinal microbiota regulates microglial activation in the CNS

Using immunohistochemistry, we quantified activated microglial cells, a hallmark of inflammatory neuropathology [48], using the marker Iba-1, in both “gray” and “white” matter areas (cortex and corpus callosum), in the sagittal brain sections of all groups of mice following FMT (Fig. 2 and Fig. S1). Iba-1^+^ microglial cell densities were higher in both the cortex and corpus callosum in old and aged mice compared with young mice (Fig. 2 and Fig. S1, Welch’s *t* test PBS groups young vs. aged cortex *P* = .005, corpus callosum *P* < .001). No significant differences were seen between the old (18 months) and aged (24 months) control, PBS-treated groups. In young mice, there were no significant differences in Iba-1^+^ cell counts between mice receiving mock transfer, coeval (young) transfer, or old (18 months) donor microbiota. However, young mice that received aged (24 months) donor microbiota had significantly more Iba-1^+^ cells in both the cortex and corpus callosum compared with all other young treatment and control groups that were at levels comparable to those in aged mice (Fig. 2B–D and Fig. S1). Conversely, in aged and old mice receiving young donor microbiota, Iba-1^+^ cells were significantly decreased in the cortex and in the corpus callosum compared with controls (Fig. 2B–D and Fig. S1).

Collectively, these data demonstrate that the intestinal microbiota strongly influences microglia activation and that heterochronic FMT modulates levels of Iba-1^+^ microglia, increasing the levels in young adult mice transplanted with an aged donor microbiota and reducing them in the CNS of aged animals receiving a young donor microbiota.

Chronic inflammation and microglial overactivation in the brain contribute to neurodegenerative pathology, which in turn can lead to cognitive decline [48]). We therefore assessed whether the reversal of inflammatory microglial upregulation in aged mice by FMT may translate to any improvement in established tests of murine cognitive function. We compared aged mice pre- and post-receipt of either a young or aged donor FMT using the Y-maze, a test of spatial working memory, and the novel object recognition (NOR) test, a measure of recognition memory. Although aged mice showed decreased performance in the NOR test at baseline, there was no significant improvement in aged mice receiving a young donor microbiota transfer compared to controls in either test (Supplementary Fig. S5). Our data does not support an impact of short-term microbial modulation by FMT on measures of working spatial and recognition memory in aged mice.

### Heterochronic FMT reverses age-associated retinal inflammation

**Fig. 3:**
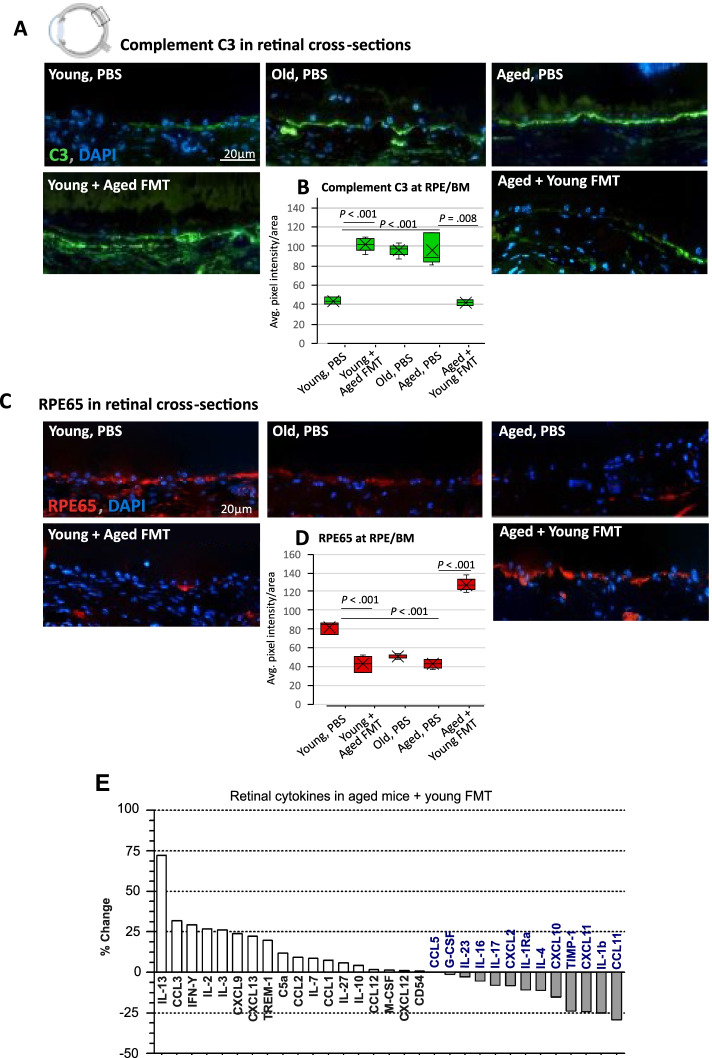
Heterochronic microbiota transfer reverses age-associated retinal inflammation and functional visual protein expression. **A** Immunostaining of complement C3 (green) in the cross-sections of the retinal pigment epithelium (RPE)/Bruch’s membrane (BM) interface from mice receiving PBS vs. heterochronic FMT. **B** Quantification of the average complement C3 staining pixel intensity (PI) per area (1280 μm^2^) at the RPE/BM interface in young, old, or aged PBS-treated mice vs. heterochronic FMT (*n* = 5 mice/group). **C** Immunostaining (RPE65, red; nuclei DAPI, blue) and quantification (**D**) of the crucial visual cycle protein RPE65 in the cross-sections of the RPE/BM interface from untreated mice vs. mice receiving heterochronic FMT, *n* = 5 mice/group. Statistical comparison between ages and between the FMT and PBS groups by Welch’s *t* test. **E** Percentage change in the expression of retinal lysate cytokine levels in aged mice receiving young donor FMT

Having identified regulatory effects of heterochronic FMT on inflammation in the CNS, both in the white and gray matter areas of the brain, we next considered the retina, as it is considered an extension or “end organ” of the central nervous system and is also especially vulnerable to age-related functional decline and inflammatory damage. Increasing expression of inflammatory complement protein C3 in the aging rodent retina is associated with progressive extracellular deposits between the retinal pigment epithelium (RPE) and Bruch’s membrane (BM), which contribute to retinal degeneration [7, 103]. Given that our results in the brain showed the most significant impact of heterochronic FMT in the young and aged groups, we characterized the retinal response to FMT in those age groups. At baseline, aged mice showed significantly more staining for complement C3 at the RPE/BM interface compared with young mice (Fig. 3A and B). Young mice receiving aged donor microbiota showed an increase in retinal complement C3 in this region (Fig. 3A, B) compared with control groups, up to levels comparable to aged mice. In contrast, aged mice receiving young donor microbiota showed a significant reduction in C3 staining compared with PBS-only aged controls, to levels comparable to those in young mice (Fig. 3A, B).

The photoreceptors of the retina are especially sensitive to fluctuations in systemic inflammation and metabolism in old age. Retinal pigment epithelial protein RPE65, which is critical for regeneration of retinal visual pigment in photoreceptors, was depleted in aged mice compared with young mice (Fig. 3C, D). In young mice, baseline levels of RPE65 were reduced post-transfer with an aged donor microbiota (Fig. 3C, D). Strikingly, the transfer of a young donor microbiota into aged mice restored RPE65 expression to levels seen in young mice (Fig. 3C, D). Retinal inflammation and degeneration have been reported to commence earlier than 24 months in rodents [22, 103, 129]. Therefore, along with our young (3 months) and aged (24 months) groups, we also assessed the old (18 months) group and found that although RPE65 staining progressively declined with age (Fig. 3D), C3 staining was not significantly different between the old (18 months) and aged (24 months) PBS groups.

We also assessed the cytokine levels in retinal lysates in aged mice receiving FMT, using a proteome profiling array. Multiple pro-inflammatory cytokines elevated in aged mice were reduced after transplantation of young microbiota including CCL11 (eotaxin), CXCL11, and IL-1β. On the other hand, IL-13 which is a putative neuroprotective cytokine [98] showed the highest increase following FMT from young donors into aged mice.

Together, our data support a causal role for the gut microbiota–retina axis in regulating retinal inflammation and age-related cytokine signaling, along with the expression of a key functional visual protein (RPE65).

### Heterochronic FMT reverses age-associated breakdown of epithelial barrier integrity and systemic inflammation

**Fig. 4:**
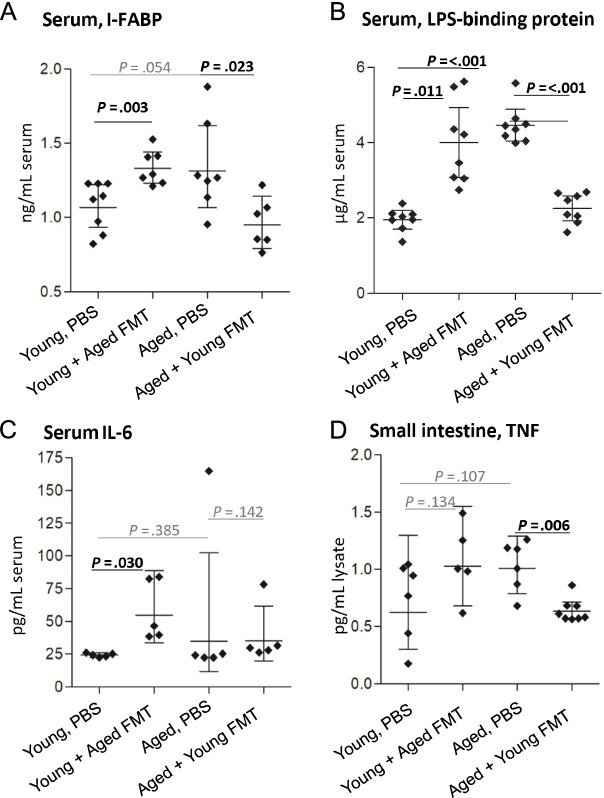
Heterochronic FMT reverses age-associated breakdown of epithelial barrier integrity and systemic inflammation. **A** ELISA analysis of serum intestinal fatty acid-binding protein (I-FABP) in young and aged mice (*n* = 6–8/group) before and after heterochronic FMT. **B** ELISA analysis of serum lipopolysaccharide-binding protein (*n* = 8/group). **C** Serum levels of IL-6 were analyzed by multiplex magnetic bead assay (Luminex) (*n* = 5/group). **D** TNF levels in small intestinal tissue lysates as measured by specific ELISA (*n* = 5–8/group). Statistical comparison between ages and between FMT and PBS groups by Welch’s *t* test; error bars denote 95% CI; significant results in bold black text

Inflammaging and pathological inflammation in the CNS have been associated with loss of intestinal epithelial barrier integrity, so-called leaky gut. Here, we investigated whether FMT had any impacts on serum levels of the intestinal epithelial cell protein intestinal fatty acid-binding protein (I-FABP), a key indicator of microbiota-related epithelial cell damage and disruption [66] and a surrogate biomarker of intestinal epithelial permeability [42, 71, 125, 127]. Serum I-FABP concentration was increased by ∼ 30% in the young mice receiving aged donor microbiota compared with young mice receiving PBS and conversely was reduced by ∼ 30% in aged mice receiving young donor microbiota compared with aged mice receiving PBS (Fig. 4A). Serum concentrations of lipopolysaccharide (LPS)-binding protein (LBP), which is secreted by the liver in response to bacterial LPS leakage into the bloodstream (and which accelerates inflammaging [56], were approximately two times higher in aged mice than in young mice (Fig. 4B) and were increased in young mice receiving an aged donor microbiota, to levels comparable to aged mice. Conversely, aged mice receiving young donor microbiota had reduced circulating concentrations of LBP, to levels comparable to young mice. Analysis of the key inflammaging-associated cytokines, IL-6 and tumor necrosis factor (TNF) [95], showed elevated concentrations of serum IL-6 in young mice receiving an aged donor microbiota, approximately twice as high as in young mice receiving PBS (Fig. 4C), with no significant difference seen between any other groups. Levels of TNF were reduced by around 40% in small intestinal lysates from aged mice receiving young donor microbiota, while pre- and post-transplant levels were not significantly different in young mice receiving an aged donor microbiota (Fig. 4D). Our cytokine analysis also included IL-12, IL-17, IL-1β, and IL-27, but none of these was above the assay threshold of detection in any group (data not shown).

Together, these data demonstrate that the aged microbiota promotes the breakdown of epithelial barrier integrity, the translocation of bacterial products, and the elevated serum levels of pro-inflammatory cytokines, all of which were reversed by transplantation with young donor microbiota.

Next, we investigated how the microbiota changes with age and following FMT, with the aim of identifying microbial signatures and features that could account for inflammaging and the beneficial outcomes of young donor FMT in aged recipients.

### Donor-derived species define the intestinal microbiota composition of FMT recipients

**Fig. 5:**
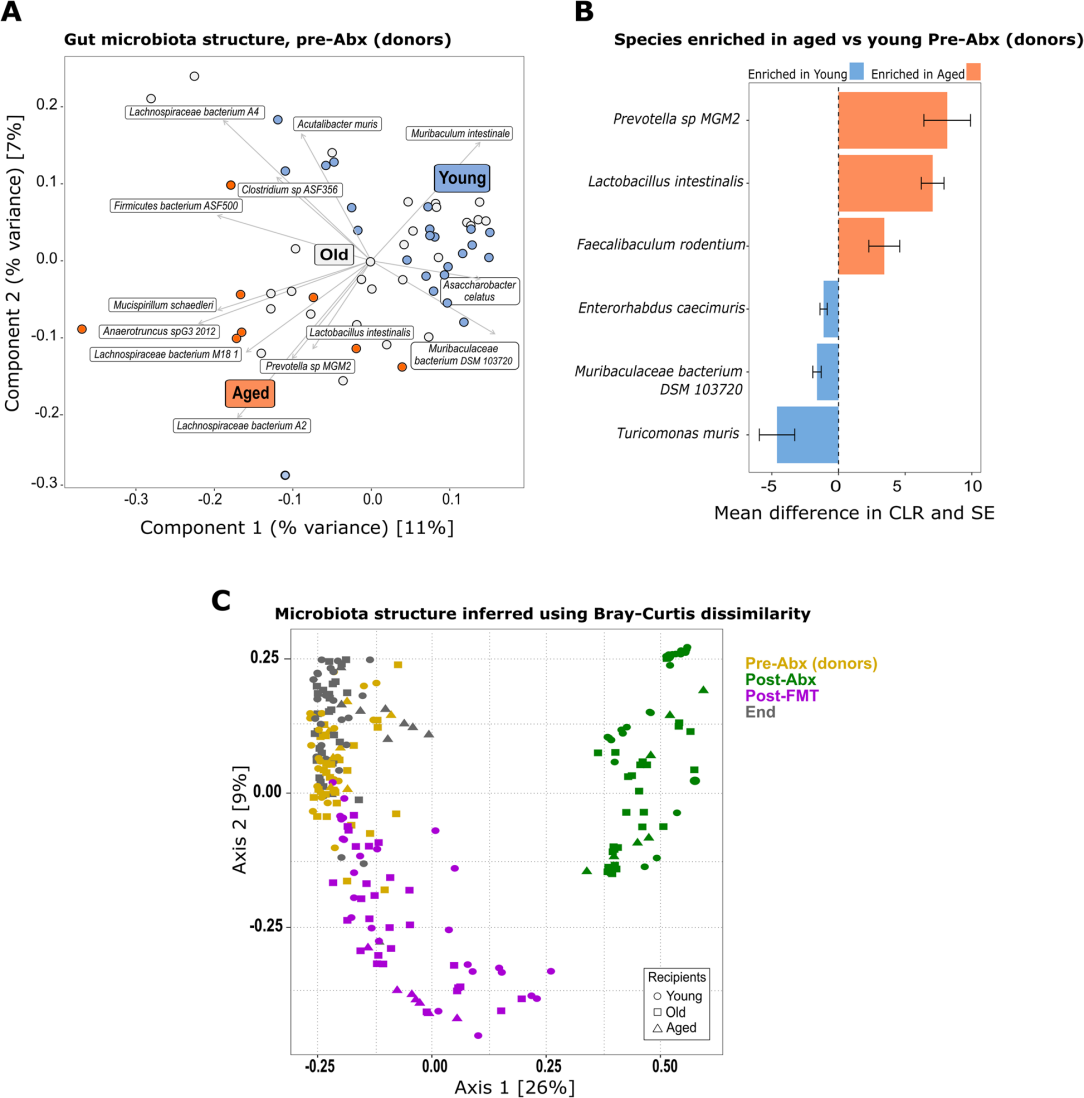
Fecal microbiota structure pre- and post-FMT. **A** PCoA showing clustering of fecal microbiota samples from young (blue), old (white), and aged (orange) mice pre-antibiotic treatment (Pre-Abx) overlaid with species driving the clustering. **B** Significantly enriched species in aged mice (orange bars, *n* = 8) vs. young mice (blue bars, *n* = 7) pre-antibiotic treatment (Pre-Abx); the results are displayed as the mean difference in centered log ratio, CLR, and SE. **C** PCoA (Bray–Curtis) depicting clustering of all mice from all age and treatment groups pre- (yellow) and post- (green) antibiotic treatment, post-FMT (magenta), and at the end of the experiment (“End,” gray). **D** PCoA (Bray–Curtis) depicting clustering of all mice from all age and treatment groups, colored by age group, young in blue markers, old in white/black markers, and aged in orange markers (see also Fig. S2)

To identify specific changes in the gut microbiota with age, and following transplantation, we performed whole-genome shotgun (WGS) metagenomic sequencing of all mice at multiple time points. Prior to FMT, we first established the baseline taxonomy of the fecal microbiota of the young, old, and aged mice. Altered microbial beta diversity profiles were seen with age (Fig. 5A, Figs. S7 and S8), as noted previously in mice [64, 112, 118] and humans [19, 25, 26, 49, 86, 87]. We identified species which drove the clustering of the age groups (Fig. 5A), including *Muribaculaceae*, *Lacnospiraceae*, *Lactobacillus*, *Clostridium*, and *Prevotella* species. *Prevotella* sp., *Lactobacillus intestinalis*, and *Faecalibaculum rodentium* were significantly enriched in the aged vs. young groups whereas *Enterorhabdus caecimuris*, *Turicimonas muris*, and *Muribaculaecae* bacterium DSM 103720 were significantly enriched in the young vs. old group (Fig. 5B and Fig. S7). A pairwise PERMANOVA showed that the intestinal microbiota was significantly different between all age groups (*P* < .01 after Benjamini–Hochberg, BH correction). The young (3 months) and aged (24 months) groups formed separate clusters in the ordination; however, the old (18 months) group did not form a separate cluster and shared features with both young and aged mice (Fig. 5A, Fig. S8). Importantly, we accounted for potential inter-cage variability, both before FMT by randomizing housing in the weeks prior to the start of the experiment and post-FMT by factoring inter-cage variation into our statistical analyses of the metagenomic data.

Using FMT, we exchanged the fecal microbiota of young, old, and aged groups of mice as detailed in Fig. 1. Fecal pellets were collected pre- and post-antibiotic treatments and at 1 and 2 weeks post-FMT. Fecal microbial and metabolite compositions were assessed by metagenomic sequencing, metabolic pathway abundance analysis, and NMR. Antibiotic pre-treatment, as expected, resulted in significant depletion of observed species across all treated groups of all ages (Fig. S2, Tukey post hoc test *P* < .01) and drastically altered the overall bacterial community composition in all age groups compared with pre-antibiotic treatment (Fig. 5C, green vs. yellow markers, 5D, S7 and S8). FMT restored the number of observed species to pre-antibiotic levels (Fig. S2). The FMT induced significant alterations in the fecal microbiota of recipients (Fig. 5C, D, Figs. S7 and S8, permutational ANOVA on dbRDA *F* = 55.8, df = 3, *P* < .01) with experimental time point accounting for 50% of the data variance (*R*^2^_adj_ = 50%). More importantly, heterochronic FMT between young and aged groups induced clear shifts in the recipient fecal microbiota composition, indicating successful divergence of the recipients’ gut microbiota from its initial composition (pairwise PERMANOVA *P* < .01 after BH correction, Fig. 5C, magenta vs. yellow markers, and Fig. S7). This was most pronounced at the “Post-FMT” time point, whereas by the end of the study, there was a greater overlap with the original donor profiles (Fig. 5C, yellow vs. gray markers), suggesting that the recipients’ intestinal environment strongly modulated long-term engraftment success.

**Fig. 6:**
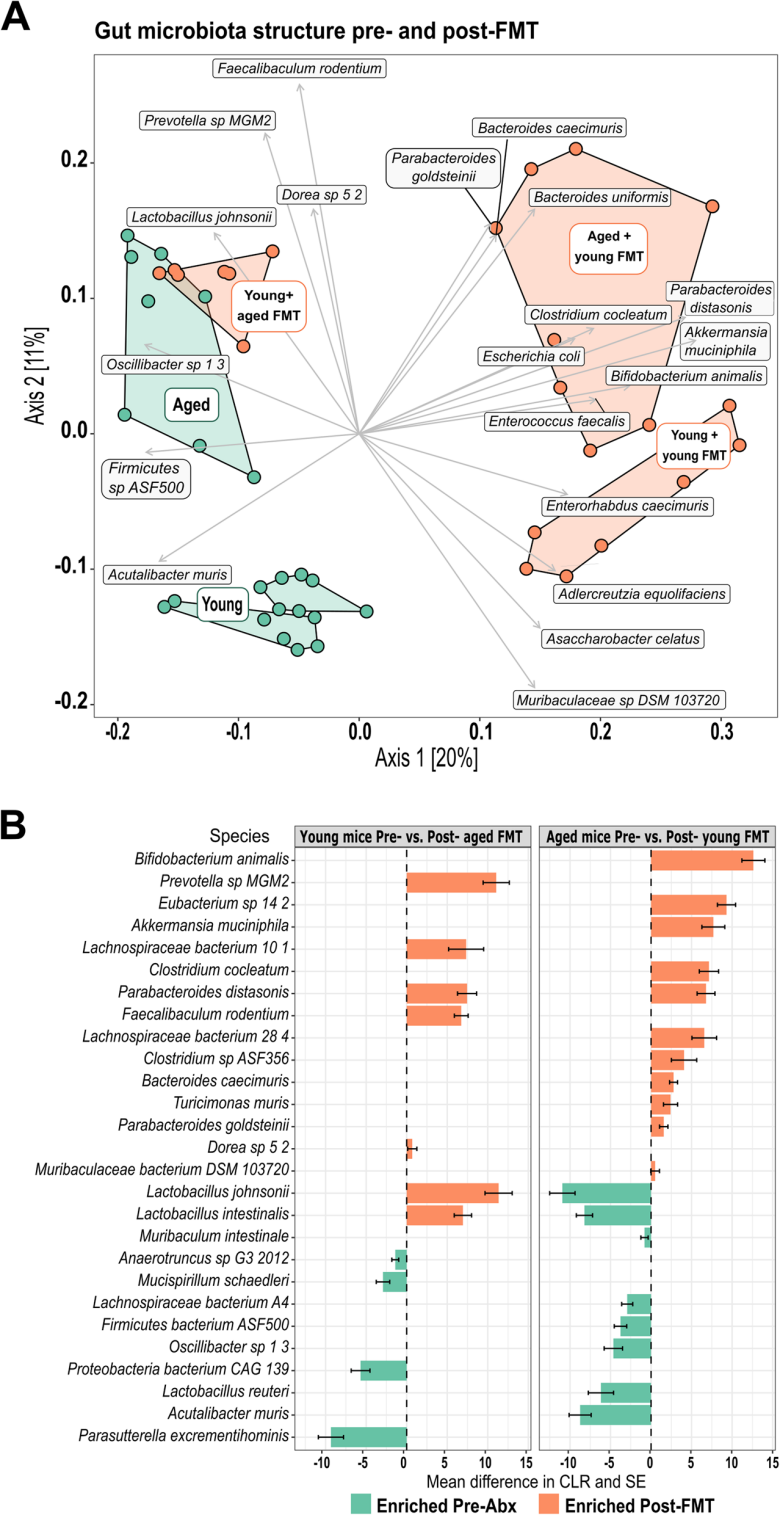
Donor-derived species define the fecal microbiota composition of FMT recipients. **A** PCoA showing clustering of fecal microbiota samples from young and aged mice Pre–Abx (green) and post-FMT (orange), and overlaid with species driving the clustering. **B** Differential abundance analysis (results displayed as the mean difference in centered log ratio, CLR, and SE) of enriched species post-heterochronic transfer (Post-FMT) vs. pre-transfer (Pre–Abx) in young and aged mice. Family abundance, young mice receiving coeval transfer, and antibiotic-only groups shown in Fig. S3

The old (18 months) group of animals had significant inter-individual variation in the microbiota composition and did not form a discrete cluster in the ordination (Fig. 5A, Fig. S8). Therefore, we focused our further metagenomic analysis on the more divergent young and aged mice, and therein on detecting the major temporal shifts in microbiota structure by comparing the “Pre-Abx” and “Post-FMT” time points (Fig. 6). Transfer of aged donor microbiota into young mice dramatically altered the recipients’ fecal microbiota composition (pairwise PERMANOVA *P* = .01, after BH correction) and resulted in a microbiota profile resembling that of aged mice at baseline (Fig. 6A). A significant compositional shift (pairwise PERMANOVA *P* = .01, after BH correction) was also induced in aged mice receiving young donor microbiota. In this case, the altered profile was distinct from that of young baseline mice, with the observed shifts following those of young mice transplanted with a young donor microbiota (Fig. 6A). This suggests that the aged donor microbiota engraftment was strongest in the young recipients, while the transfer of young donor microbiota was more susceptible to modification by the influence of the FMT procedure, or the recipient intestinal environment, or both.

Species belonging to the *Oscillibacter* and *Prevotella* genera, an unknown species in the Firmicutes phylum, and *Lactobacillus johnsonii* drove the clustering of aged mice and that of young mice transplanted with aged donor microbiota (Fig. 6A). In contrast, the divergence of young mice receiving young donor microbiota and aged mice receiving young donor microbiota was driven by species within the genera *Bifidobacterium*, *Akkermansia*, *Parabacteroides*, *Clostridium*, and *Enterococcus* (Fig. 6A).

Members of the *Bifidobacteriaceae*, *Akkermansiaceae*, and *Eubacteriaceae* families that are associated with beneficial effects on intestinal and extra-intestinal health in both mice and humans ([8, 38, 43, 73, 78, 81]) were more abundant in both young and aged recipients of young donor microbiota (Fig. S3A, differentially abundant families listed in Table S2).

Transplanting young mice with an aged donor microbiota resulted in enrichment of the signature species of the aged mice, namely *Prevotella* sp. MGM2, and *L. intestinalis*, along with *Lachnospiraceae bacterium* 10–1, *Parabacteroides distasonis*, and *L. johnsonii* (Fig. 6A, B).

Conversely, transplanting aged mice with a young donor microbiota resulted in enrichment of signature species of the young mice, *T. muris* and *Muribaculaceae* bacterium DSM103720, along with *Bifidobacterium animalis*, *Eubacterium* sp14–2, *Akkermansia muciniphila*, and six other species (Fig. 6A, B). *Enterorhabdus caecimuris*, although slightly enriched in young mice, was not significantly enriched in aged mice receiving young donor microbiota (Fig. 6A, B).

The most significantly enriched species in aged mice receiving young donor microbiota (*Bifidobacterium animalis*, *Eubacterium* sp. 14–2, *A. muciniphila*, and *Clostridium cocleatum*) were also enriched in young mice recolonized with young donor microbiota, although others (*Parabacteroides distasonis*, *Lachnospiraceae* 28–4, *Clostridium* ASF356) were only enriched in the aged recipients (Fig. 6B and Fig. S3B), consistent with an impact of the recipient intestinal environment on engraftment success of different species.

We confirmed that enriched species in aged mice receiving young donor FMT did not reflect only those species which were not eliminated, or were better able to flourish after antibiotic treatment, by cross-checking against species which were enriched post-antibiotics only in controls (Fig. S3C). A table of differentially abundant species is provided in Table S1.

Collectively, our results showed that heterochronic FMT resulted in significant alteration of the recipient microbiota structure, enriching for bacterial species distinctive of the donor age group, and that both donor and recipient were influential in the resulting microbiota composition.

To estimate whether the FMT interventions altered potential microbial functional profiles in recipients, and to identify potential molecular mediators of the effects seen in the gut, brain, and retina, we next analyzed microbial metabolic pathway abundance in the WGS data to infer functional capacity and, in parallel, analyzed fecal metabolite concentrations by NMR in young and aged mice pre- and post-FMT.

### Heterochronic FMT alters lipid and vitamin metabolism

Microbial metabolites are key mediators of microbiota function and influencers of host cell physiology and metabolism. Targeted nuclear magnetic resonance (^1^H–NMR) spectroscopy-based metabolomics of fecal samples from young and aged mice, pre- and post-heterochronic FMT, was performed primarily to assess amino acids, sugars, short-chain fatty acids (SCFA), and alcohols (the metabolites detected, and their raw concentrations are detailed in Table S3). Distance-based redundancy analysis (dbRDA) showed high inter-individual variation with only 24% of variance explained by treatment groupings (4% of variance was explained by cage groupings). Permutational ANOVA on the modeling suggested significant differences among the groups (*F* = 2.27, df = 3, *P =* .003). However, subsequent pairwise PERMANOVA comparisons on the Euclidean distance matrix found no statistically significant differences between any two groups (.05 < *P* < .1 after BH correction).

Using a partial least squares-discriminant analysis (PLS–DA), we identified metabolites with the strongest influence on defining the separation between groups (Figs. S4A and S4B). Overall, metabolic profiles were broadly similar, with only a limited degree of variation in some metabolites across age and treatment groups. In young mice receiving aged donor microbiota, these included metabolites involved in amino acid metabolism (malate threonine and creatine) and sugar metabolism (fucose and sucrose). In aged mice receiving young donor microbiota, the greatest contributors to the loadings were tauro-conjugated bile acids, acetate, the polyamine putrescine, and polyamine regulator ornithine, metabolites previously associated with beneficial effects in aging [74, 94].

**Fig. 7:**
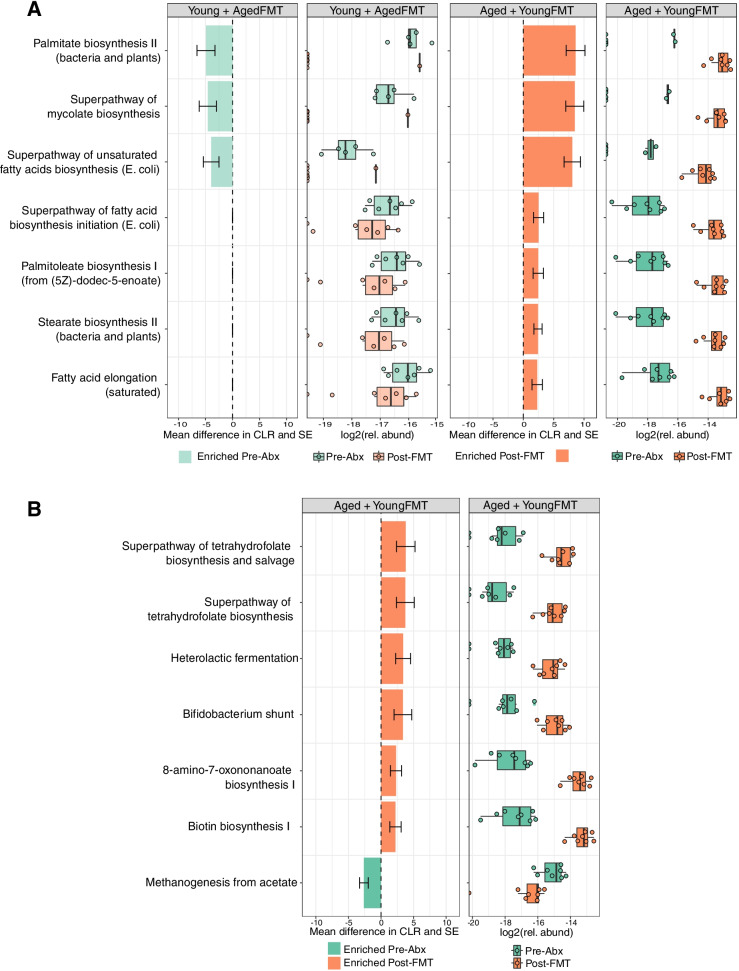
Heterochronic transfer alters lipid and vitamin metabolism. **A** Selected lipid metabolism pathways significantly enriched or depleted in young and aged mice following heterochronic FMT. **B** Other selected pathways, which were significantly enriched or depleted in aged mice receiving young FMT. The results are displayed as the mean difference in centered log ratio (CLR); error bars denote standard error. Boxplots depict log2 transformed relative abundance of individual pathways

In parallel, we analyzed functional metabolic pathway abundances in the metagenomic data. In aged mice receiving young donor microbiota, we detected significant enrichment of multiple pathways involved in lipid synthesis compared with pre-transfer samples (Fig. 7A) and, in particular, pathways involved in the synthesis of medium- and long-chain fatty acids (MCFA, LCFA). Significantly, some of these lipid metabolism pathways were depleted in young mice receiving aged donor microbiota, including biosynthesis of palmitate, mycolate, and unsaturated fatty acids. Other pathways enriched in aged mice transplanted with young donor microbiota included those involved in the biosynthesis of the vitamins B7 and B9 (biotin and folate) (Fig. 7B). There was also a reduction in methanogenesis suggestive of some impact on gut archaea, an increase in heterolactic fermentation, utilized by some *Lactobacillus* species, and an increase in the *Bifidobacterium* shunt pathway (Fig. 7B), the latter in keeping with the increase in proportional abundance of *Bifidobacterium* spp. in this group (Fig. 6B). A full list of differentially enriched metabolic pathways pre- and post-FMT is provided in Table S4.

In summary, heterochronic FMT resulted in significant shifts in lipid and vitamin metabolism pathways, increasing their proportional abundance in aged mice transplanted with the microbiota of young donors and decreasing their abundance in young mice receiving aged donor microbiota. We also identified enrichment of heterolactic fermentation and *Bifidobacterium* shunt in aged mice receiving young donor FMT.

## Discussion

Altered intestinal microbiota composition in old age is associated with chronic low-grade inflammation and increasing susceptibility to age-associated chronic diseases. However, whether age-associated changes in the diversity, composition, or function of the gut microbiota are causal in disease etiology remains largely unknown. Here, we used FMT between young and aged mice, to bi-directionally modulate recipient microbiota structure and metabolic capability. We show that aged donor microbiota transfer to young mice drives inflammation and loss of integrity in the intestinal epithelial barrier elevated systemic and tissue markers of inflammaging, and upregulated inflammation in the retina and brain. The transferred aged microbiota and resulting recipient microbiota composition were distinguished by enrichment for *Prevotella*, *Lacnospiraceae*, and *Facecalibaculum* species and depletion of long-chain fatty acid synthesis. Conversely, transfer of aged mice with young donor microbiota, which was distinguished by enrichment *Bifidobacteria*, *Eubacteria*, and *Akkermansia* species, and enrichment for B vitamin biosynthesis and lipid synthesis pathways reversed inflammatory changes in the aged gut, brain, and retina. Our data support the suggestion that altered gut microbiota in old age contributes to intestinal and systemic inflammation, and so may contribute to driving inflammatory pathologies of aged organs. Targeting the gut–brain axis in aging, by modification of microbial composition to modulate immune and metabolic pathways, may therefore be a potential avenue for therapeutic approaches to age-associated inflammatory and functional decline.

### Signature microbes of the aging GI tract pre- and post-FMT

We identified age-associated signature bacteria which, after transfer by heterochronic FMT, become prominent within the recipients’ engrafted microbiota. Importantly, this analysis accounted for any potential inter-cage variability, by factoring inter-cage variation into our statistical analyses. In all animals, the shift in microbial structure induced by heterochronic FMT was most apparent at 7 days post-FMT. By 18 days post-FMT, the transplanted microbiota more closely resembled the baseline, pre-antibiotic treatment microbiota profile (correlation in a symmetric Procrustes rotation = 25%, *P* = .05). This suggests that age and environmental factors in the GI tract of the recipient provide particular ecological niches favoring specific bacterial taxa.

This adaptation over time however did not limit the impact of the transplanted microbiota on tissues, in which changes in key functional cell types and biomarkers of health were apparent at 18 days post-FMT, the point of tissue collection. This raises the question of the stability of the engrafted microbiota and whether long-term persistence is necessary to maintain beneficial health effects, which could be addressed in larger long-term studies.

In both aged mice and in young recipients of aged donor microbiota, we saw significant enrichment of *Prevotella* sp_MGM2. *Prevotella* species associated with detrimental impacts on intestinal barrier integrity are enriched in IBS patients and in models of intestinal inflammation [52, 113] and may promote inflammatory arthritis [3, 104, 126]. However, other reports identify beneficial impacts of *Prevotella* species on glucose metabolism [60], and there are conflicting associations with Parkinson’s disease progression [97].

*Bifidobacterium animalis* and *Akkermansia muciniphila* were among the most enriched taxa in young mice and in aged mice receiving a young donor microbiota and are associated with promoting intestinal and extra-intestinal health and healthy aging. Furthermore, *A. muciniphila* is depleted in models of inflammation and metabolic disorders and displays probiotic potential, by regulating mucus production and epithelial barrier integrity and by reducing serum LPS [38]. This latter impact on LPS is in accordance with the reduced serum LBP levels we saw in young mice and aged mice receiving young donor microbiota. In mouse models of accelerated aging, supplementation with *A. muciniphila* can also attenuate age-related decline in colon thickness and immune activation [73], and exert beneficial effects on lifespan and healthspan [8].

Other species we found enriched in aged recipients of young donor microbiota included *B. animalis*, a probiotic species positively associated with longevity in mice [78] and negatively correlated with inflammation and human obesity [81], and *Eubacterium* sp. 14–2. The precise classification of many species within the *Eubacteriaceae* family is under review. However, some species are currently thought to modulate cholesterol and bile acid metabolism and are considered potential therapeutics [43].

Overall, our data indicate that FMT with microbiota from young mice leads to an enrichment of beneficial taxa in aged mice. Refined colonization studies using single species, or defined consortia, would help to define the contributions of particular taxa to the systemic effects we have described, and to identify their specific mechanisms of action. It also remains unknown whether specific microbial species mediate their effects through unique bacterial products, or by expansion and potential colonization of new niches. Our procedures were optimized to assess the bacterial fraction of the intestinal microbiota, and as such, we cannot exclude potential contributions from other microbial constituents in the effects we describe. The identification of small differences in the abundance of the methanogenesis pathway following FMT suggests a possible shift in archaeal metabolism or abundance between our treatment groups; however, further investigation to evaluate this and other components, which may have significant impacts on host health, will require the use of additional protocols optimized for fungal [53], viral [30, 34], and archaeal [77] extraction and identification.

### The aged intestinal microbiota drives barrier permeability and inflammation

A central question regarding inflammaging and age-associated intestinal pathology is whether breakdown of epithelial barrier integrity and function drives changes in the intestinal microbiota, or, whether a shift in microbiota composition leads to epithelial damage and permeability, or if both occur concomitantly [2, 128]. Our data show that transplanting an aged donor microbiota into young recipients results in elevated intestinal concentrations of TNF, and elevated surrogate biomarkers of altered epithelial barrier permeability (I-FABP, LBP), supporting a driving role for the age-associated microbiota in the breakdown of barrier integrity and promotion of systemic inflammatory responses. That these effects are reversed in aged recipients of young donor microbiota supports this hypothesis, suggesting a positive impact of the microbiota of young mice on barrier integrity, or that removal of age-associated microbiota allows for barrier recovery. In agreement with our findings and interpretation is the observation that the microbiota from aged donors exacerbates paracellular permeability and inflammation in germ-free mice [40, 118].

### The intestinal microbiota regulates CNS microglia activation

In mammalian models, altered microglial morphology and function, and elevated expression of microglial activation markers are seen with advancing age [82]. However, it is unclear whether age and absolute numbers of activated microglia in different regions of the brain are positively associated. Here, we observed higher numbers of Iba-1^+^ microglia in both the corpus callosum and the cortex with age in healthy mice. Further, we show that Iba1^+^ cell density in both regions is regulated by the age-associated gut microbiota. Transplanting young donor microbiota reduced elevated numbers of Iba-1^+^ microglia in the CNS of aged recipients, while aged donor microbiota increased Iba-1^+^ cells in the CNS of young recipients. These striking changes in microglia activation could reflect expansion and/or contraction in total microglial cell number, or modulation of Iba-1^+^ expression in resident populations, or both. CNS microglial populations constantly and rapidly remodel [6], making it difficult to discriminate between changes in cell number versus changes in Iba-1 expression in the setting of FMT. Long-term imaging, lineage-tracing mouse models, and pulse-chase studies would be useful in addressing these distinctions.

Loss of intestinal barrier integrity and elevated circulating pro-inflammatory cytokine concentrations are linked with the polarization of CNS microglia toward a pro-inflammatory phenotype [11, 73, 109] and promote neurodegenerative disease [10, 13, 48]. In accordance with these data, we saw disrupted barrier integrity and increased bacterial translocation following the transfer of aged donor microbiota into young recipients.

Modulation of metabolism is another way in which FMT may impact microglial activation. Here, we found lipid metabolism pathways were significantly altered by heterochronic FMT, particularly in aged mice receiving young donor microbiota. Evidence for unsaturated, mono-, and poly-unsaturated fatty acids impacting microglial function comes from both in vitro and in vivo studies [70]. Palmitate, for example, which here was enriched in the aged mice receiving young donor microbiota, has anti-inflammatory effects in microglial cells [58, 80, 119]. Targeted metabolomics approaches and regional sampling would aid further investigation by determining levels of specific metabolites identified by our analysis along the GI tract. Other possible mediators of FMT impacts on CNS inflammation include inflammatory cytokine signaling and immunomodulatory factors induced by members of the microbiota and gut–brain transport of microbial products including microvesicles which can cross the blood–brain barrier [45, 47, 124]. Of note, Iba-1 numbers also appeared reduced in antibiotic-treated mice, suggesting that depletion or elimination of detrimental species, may be more significant in regulating microglial inflammation than the introduction of beneficial species. The antibiotics used in this study are, to varying degrees, able to penetrate the blood–brain barrier and cerebrospinal fluid. It is therefore theoretically possible that treatment with antibiotics could also affect microglial reactivity in the brain by depleting any local CNS-associated microbes, although the existence of a brain-associated microbiome remains highly controversial.

Young mice transplanted with an aged donor microbiota display defects in spatial learning and memory [33], suggesting the possibility that in the reverse situation, young donor FMT to aged mice could potentially have beneficial impacts on cognitive measures. However, both this study and a recent study also using young donor FMT in aged mice [15] showed no beneficial effect of transplantation with young donor fecal microbiota using behavioral tests of short-term spatial learning and memory. Longer-term assessment using the Morris Water Maze test of aged mice following repeated young donor FMT over 4 weeks did show some improvement for aged recipients of young vs. aged donor FMT [15], suggesting that impacts on behavior may require more subtle analysis and that substantially altering behavioral phenotypes may require sustained microbial intervention. Extrapolating the relevance of our findings to co-morbidities of aging in humans would require comparable FMT studies in elderly individuals. There is some evidence for improved cognition with probiotic use in those with pre-existing cognitive impairment [31], although the utility and effectiveness of single or small consortia of beneficial microbes in modulating age-associated neuro-inflammatory and retinal diseases remain to be determined.

### The intestinal microbiota regulates retinal inflammation

Association studies have linked intestinal microbial products with the development and progression of age-related choroidal neovascularization (CNV) and age-related macular degeneration (AMD) [4, 131, 133]. Here, we provide the first direct evidence that aged intestinal microbiota drives retinal inflammation and regulates expression of the functional visual protein RPE65 at the RPE/BM interface. Polymorphisms in human complement regulatory factor H (CFH) predispose individuals to AMD [35, 46, 59] and CFH deficiency in mice causes retinal abnormalities, including accumulation of complement C3, resulting in significant visual dysfunction [28]*.* We found high levels of C3 expression in aged mice were reversed after receiving a young donor microbiota. Conversely, transplanting an aged donor microbiota into young mice resulted in elevated levels of retinal C3 expression, comparable to levels in aged mice. RPE65 is vital for maintaining normal photoceptor function via trans-retinol conversion; mutations or loss of function are associated with retinitis pigmentosa and are implicated in AMD [21]. Our finding that age-associated decline in host retinal RPE65 expression is induced by an aged donor microbiota, and conversely is rescued by young donor microbiota transfer, suggests age-associated gut microbiota functions or products regulate visual function.

Retinal degeneration induces upregulation of a cytokine and chemokine production whose expression is coordinated by Müller cells, microglia, and RPE [102]. Transfer of young donor microbiota results in a decrease in aged mice of cytokines involved in inflammatory immune cell recruitment and pro-inflammatory signaling, including CCL11 (eotaxin), CXCL11, and IL-1β [23, 62, 84], all of which are elevated in aged mouse retinae [108]. Elevated CCL11 in the plasma and cerebrospinal fluid seen in aging mice and humans are associated with declining neurogenesis, neurodegenerative disease, and cognitive decline and may contribute to driving neovascularisation in AMD via interaction with CCR3 [1, 93, 123]. Upregulation of CXCL11 is seen in RPE cells adjacent to drusen deposits in AMD [62], and its secretion can be triggered by proinflammatory cytokines including TNF and IL-1β. In addition, IL-1β is associated with the progression of retinal pathologies, including AMD [130]. Together, our results show that transfer of young microbiota to aged mice reduces the expression of key cytokines associated with driving inflammation and disease in the retina.

One plausible scenario by which the microbiota influences complement expression in the retina is that these changes are a consequence of leakage of microbial products from the gut into the systemic circulation and/or upregulation of systemic inflammatory cytokine signaling, in both aged mice and in young mice treated with aged donor FMT. This is particularly impactful on tissues such as the retina with high metabolic and circulatory input and demand. It is also possible that upregulated complement expression by the liver as part of an acute phase response to bacterial toxins in the blood, as supported by our finding that heterochronic FMT altered circulating LBP levels, leads to accumulation of complement proteins at retinal membranes.

Our metagenomic pathway analysis identified a positive impact of young donor microbiota on key vitamins important for maintaining eye health during aging. Vitamin B synthesis pathways, important in photoreceptor maintenance and regulation of retinal vascular health, were enriched in aged recipients of young donor microbiota, while being depleted in young recipients of aged donor microbiota. Deficiencies in B vitamins are implicated in retinitis pigmentosa and glaucoma, for which improved outcomes can be achieved by dietary supplementation [24, 114]. It is currently unclear how much of the B vitamin production by gut microbiota is accessible by the host; however, our results suggest that microbial modulation, combined with dietary or pharmacological supplementation of B vitamin levels to modulate retinal RPE65 expression, could have potential therapeutic value in the treatment of retinal diseases and in general maintenance of eye health in old age.

## Conclusions

Our results demonstrate that the age-associated changes in the murine intestinal microbiota contribute to disrupted gut barrier integrity and systemic and tissue inflammation affecting the retina and the brain, but these changes can be reversed by replacement with young donor microbiota. Further work to assess the long-term persistence of the beneficial effects of young donor microbiota transfer, in the eye and brain, will establish whether FMT can promote long-term health benefits in aged individuals and ameliorate age-associated neurodegeneration and retinal functional deterioration.

## Supplementary Information

**Additional file 1: Table S1.** Differential abundance table (species). Relates to Fig. 6B and Figure S3B. Provided as a separate Excel file.

**Additional file 2: Table S2.** Differential abundance table (family). Relates to Figure S3A. Provided as a separate Excel file.

**Additional file 3: Table S3.** Raw fecal metabolite concentrations from NMR analysis. Relates to Figure S4. Provided as a separate Excel table.

**Additional file 4: Table S4.** Full list of differentially abundant metabolic pathways Pre– vs. Post–FMT. Relates to Fig. 7. Provided as a separate Excel file.

**Additional file 5: Figure S1.** Related to Fig. 2. Iba-1^+^ cell density in corpus callosum is regulated by the intestinal microbiota. (**A**) Iba-1^+^ microglia were identified by immunostaining (red), nuclei counterstained with Hoechst (blue) in the corpus callosum (highlighted in cartoon) of sagittal brain sections. (**B**) Representative immunostaining of young, old, and aged mice, either treated with PBS only, treated with antibiotics (Abx) only, or with antibiotics followed by FMT from young, old, or aged donors. Quantified in Fig. 2. **Figure S2.** Related to Fig. 5. Impact of antibiotics and microbiota transfers on observed species. Number of observed bacterial species pre– and post– antibiotic treatment, post–FMT and at the end of the experiment. Pairwise comparison, Kruskal–Wallis, * = *P* < .0001. **Figure S3.** Related to Fig. 6. Clustering of beta–diversity post–FMT is driven by donor age, and differential abundance of bacterial families identified pre–and post–transfer. (**A**) Differential abundance of bacterial families in aged mice receiving young donor microbiota, and in young mice receiving young, old, or aged donor microbiota. (**B**) Species enriched in the young mice receiving young donor microbiota. (**C**) Microbiota surviving/enriched post–antibiotics only. **Figure S4.** Metabolite profiles in fecal pellets estimated by (1 H)–NMR. (**A**) PLS–DA comparing fecal metabolite profiles (full list of metabolites in Supplementary Table S3) of young and aged groups pre– and post–heterochronic transfer estimated by NMR. (**B**) Specific metabolites contributing to the loading of component 1, 2, and 3 in the PLS–DA analysis. **Figure S5.** Behavioral testing in aged mice shows no difference between aged mice receiving young vs. aged donor FMT. **(A)** Novel object recognition (NOR) test and (**B**) Y -maze test results for: young mice (n = 11), aged mice (n = 20), aged mice+ young donor FMT (Y-FMT) (n = 10) and aged mice + aged donor FMT (A-FMT) (n = 10). Error bars denote 95% CI. **Figure S6.** Per-sample read numbers in metagenomic sequencing data. (**A**) Beeswarm plot, (**B**) histogram, and (**C**) scatter plot, depicting per-sample read numbers in trimmed and decontaminated metagenomic sequencing data. Samples from all mice from all groups. **Figure S7.** Bubble plot depicting variation between metagenomic samples across mice within young and aged groups pre- and post-FMT. **Figure S8.** Bubble plot depicting variation between metagenomic samples across mice within old groups pre- and post-FMT.

**Additional file 6. **Key Resources Table.

## Data Availability

Further information and requests for resources and reagents should be directed to, and will be fulfilled by, the lead contact, Simon R. Carding (simon.carding@quadram.ac.uk).

## Abbreviations

Abx: Antibiotics
AMD: Age-related macular degeneration
BM: Bruch’s membrane
C3: Complement component 3
CNS: Central nervous system
FMT: Fecal microbiota transplant
GI: Gastrointestinal
Iba-1: Ionized calcium-binding adaptor protein1
I-FABP: Intestinal fatty acid-binding protein
LBP: Lipopolysaccharide-binding protein
LPS: Lipopolysaccharide
NGS: Next-generation sequencing
NMR: Nuclear magnetic resonance
PBS: Phosphate-buffered saline
RPE: Retinal pigment epithelium
RPE65: Retinal pigment epithelium-specific 65 kDa protein
(S/M/L) CFA: Short-, medium-, long-chain fatty acids
SPF: Specific pathogen-free
TNF: Tumor necrosis factor
WGS: Whole-genome shotgun
