# Supplementary material for: Fecal microbiota transfer between young and aged mice reverses hallmarks of the aging gut, eye, and brain
Source: Microbiome. 2022 Apr 29;10:68. doi: 10.1186/s40168-022-01243-w (PMC9063061; doi:10.1186/s40168-022-01243-w)
Supplement: Supplementary file 2 — Additional file 1: Table S1. Differential abundance table (species). Relates to Fig. 6B and Figure S3B. Provided as a separate Excel file. [file 40168_2022_1243_MOESM2_ESM.pdf]

Table S1. Significant differentially-abundant species

Species	contrast2	estimate	SE	df	lower.CL	upper.CL	t.ratio	p.value	padjust
Bacteriap_Actinobacteriac_Actinobacteriao_Bifidobacterialesf_Bifidobacteriaceaeg_Bifidobacteriums_Bifidobacterium_animalis.1	YY-PFMT_YY	-12.53067	1.51812	102.37710	-15.54173	-9.51962	-8.25406	0.00000	0.00000
Bacteriap_Actinobacteriac_Actinobacteriao_Bifidobacterialesf_Bifidobacteriaceaeg_Bifidobacteriums_Bifidobacterium_animalis.2	YM-PFMT_YM	-1.22792	1.72376	108.72150	-4.64445	2.18861	-0.71235	0.47777	0.61535
Bacteriap_Actinobacteriac_Actinobacteriao_Bifidobacterialesf_Bifidobacteriaceaeg_Bifidobacteriums_Bifidobacterium_animalis.3	OY-PFMT_OY	-12.62526	1.42007	102.37710	-15.44185	-9.80867	-8.89056	0.00000	0.00000
Bacteriap_Actinobacteriac_Actinobacteriao_Bifidobacterialesf_Bifidobacteriaceaeg_Bifidobacteriums_Bifidobacterium_animalis.4	YO-PFMT_YO	-1.38077	1.51812	102.37710	-4.39183	1.63028	-0.90953	0.36521	0.54599
Bacteriap_Actinobacteriac_Coriobacteriiao_Eggerthellalesf_Eggerthellaceaeg_Adlercreutzias_Adlercreutzia_equolifaciens.1	YY-PFMT_YY	0.45592	0.56882	102.39161	-0.67229	1.58412	0.80151	0.42469	0.58066
Bacteriap_Actinobacteriac_Coriobacteriiao_Eggerthellalesf_Eggerthellaceaeg_Adlercreutzias_Adlercreutzia_equolifaciens.2	YM-PFMT_YM	0.38258	0.64577	108.75057	-0.89735	1.66251	0.59244	0.55479	0.67434
Bacteriap_Actinobacteriac_Coriobacteriiao_Eggerthellalesf_Eggerthellaceaeg_Adlercreutzias_Adlercreutzia_equolifaciens.3	OY-PFMT_OY	-0.26372	0.53209	102.39161	-1.31906	0.79162	-0.49563	0.62121	0.72141
Bacteriap_Actinobacteriac_Coriobacteriiao_Eggerthellalesf_Eggerthellaceaeg_Adlercreutzias_Adlercreutzia_equolifaciens.4	YO-PFMT_YO	1.07330	0.56882	102.39161	-0.05491	2.20150	1.88687	0.06201	0.14129
Bacteriap_Actinobacteriac_Coriobacteriiao_Eggerthellalesf_Eggerthellaceaeg_Asaccharobacters_Asaccharobacter_celatus.1	YY-PFMT_YY	0.52722	0.54182	102.42253	-0.54741	1.60186	0.97307	0.33281	0.52359
Bacteriap_Actinobacteriac_Coriobacteriiao_Eggerthellalesf_Eggerthellaceaeg_Asaccharobacters_Asaccharobacter_celatus.2	YM-PFMT_YM	0.44420	0.61491	108.80511	-0.77456	1.66296	0.72238	0.47161	0.61535
Bacteriap_Actinobacteriac_Coriobacteriiao_Eggerthellalesf_Eggerthellaceaeg_Asaccharobacters_Asaccharobacter_celatus.3	OY-PFMT_OY	-0.48772	0.50682	102.42253	-1.49295	0.51751	-0.96231	0.33816	0.52473
Bacteriap_Actinobacteriac_Coriobacteriiao_Eggerthellalesf_Eggerthellaceaeg_Asaccharobacters_Asaccharobacter_celatus.4	YO-PFMT_YO	1.37660	0.54182	102.42253	0.30196	2.45124	2.54071	0.01256	0.04112
Bacteriap_Actinobacteriac_Coriobacteriiao_Eggerthellalesf_Eggerthellaceaeg_Enterorhabduss_Enterorhabdus_caecimuris.1	YY-PFMT_YY	0.32029	0.32907	102.37476	-0.33239	0.97297	0.97331	0.33269	0.52359
Bacteriap_Actinobacteriac_Coriobacteriiao_Eggerthellalesf_Eggerthellaceaeg_Enterorhabduss_Enterorhabdus_caecimuris.2	YM-PFMT_YM	0.52651	0.37376	108.66142	-0.21429	1.26731	1.40869	0.16178	0.28030
Bacteriap_Actinobacteriac_Coriobacteriiao_Eggerthellalesf_Eggerthellaceaeg_Enterorhabduss_Enterorhabdus_caecimuris.3	OY-PFMT_OY	-0.08882	0.30782	102.37476	-0.69935	0.52170	-0.28856	0.77350	0.84382
Bacteriap_Actinobacteriac_Coriobacteriiao_Eggerthellalesf_Eggerthellaceaeg_Enterorhabduss_Enterorhabdus_caecimuris.4	YO-PFMT_YO	0.64895	0.32907	102.37476	-0.00373	1.30163	1.97206	0.05130	0.12650
Bacteriap_Bacteroidetesc_Bacteroidiao_Bacteroidalesf_Bacteroidaceaeg_Bacteroidess_Bacteroides_caecimuris.1	YY-PFMT_YY	-0.44138	0.52744	102.35231	-1.48752	0.60475	-0.83684	0.40463	0.56460
Bacteriap_Bacteroidetesc_Bacteroidiao_Bacteroidalesf_Bacteroidaceaeg_Bacteroidess_Bacteroides_caecimuris.2	YM-PFMT_YM	-0.58069	0.59905	108.66553	-1.76803	0.60664	-0.96936	0.33452	0.52359
Bacteriap_Bacteroidetesc_Bacteroidiao_Bacteroidalesf_Bacteroidaceaeg_Bacteroidess_Bacteroides_caecimuris.3	OY-PFMT_OY	-2.77725	0.49338	102.35231	-3.75583	-1.79868	-5.62906	0.00000	0.00000
Bacteriap_Bacteroidetesc_Bacteroidiao_Bacteroidalesf_Bacteroidaceaeg_Bacteroidess_Bacteroides_caecimuris.4	YO-PFMT_YO	0.91040	0.52744	102.35231	-0.13574	1.95654	1.72606	0.08735	0.19175
Bacteriap_Bacteroidetesc_Bacteroidiao_Bacteroidalesf_Bacteroidaceaeg_Bacteroidess_Bacteroides_thetaiotaomicron.1	YY-PFMT_YY	-0.85266	1.23188	102.42077	-3.29598	1.59066	-0.69216	0.49040	0.62164
Bacteriap_Bacteroidetesc_Bacteroidiao_Bacteroidalesf_Bacteroidaceaeg_Bacteroidess_Bacteroides_thetaiotaomicron.2	YM-PFMT_YM	-1.82666	1.39848	108.74425	-4.59847	0.94516	-1.30617	0.19425	0.33300
Bacteriap_Bacteroidetesc_Bacteroidiao_Bacteroidalesf_Bacteroidaceaeg_Bacteroidess_Bacteroides_thetaiotaomicron.3	OY-PFMT_OY	-0.85899	1.15232	102.42077	-3.14450	1.42652	-0.74544	0.45771	0.60580
Bacteriap_Bacteroidetesc_Bacteroidiao_Bacteroidalesf_Bacteroidaceaeg_Bacteroidess_Bacteroides_thetaiotaomicron.4	YO-PFMT_YO	2.02451	1.23188	102.42077	-0.41881	4.46783	1.64343	0.10336	0.21634
Bacteriap_Bacteroidetesc_Bacteroidiao_Bacteroidalesf_Bacteroidaceaeg_Bacteroidess_Bacteroides_uniformis.1	YY-PFMT_YY	0.56541	0.91683	102.21291	-1.25306	2.38389	0.61671	0.53880	0.66885
Bacteriap_Bacteroidetesc_Bacteroidiao_Bacteroidalesf_Bacteroidaceaeg_Bacteroidess_Bacteroides_uniformis.2	YM-PFMT_YM	0.31481	1.04560	107.73157	-1.75781	2.38742	0.30108	0.76394	0.83847
Bacteriap_Bacteroidetesc_Bacteroidiao_Bacteroidalesf_Bacteroidaceaeg_Bacteroidess_Bacteroides_uniformis.3	OY-PFMT_OY	-0.75751	0.85761	102.21291	-2.45854	0.94352	-0.88328	0.37916	0.54599
Bacteriap_Bacteroidetesc_Bacteroidiao_Bacteroidalesf_Bacteroidaceaeg_Bacteroidess_Bacteroides_uniformis.4	YO-PFMT_YO	-0.12023	0.91683	102.21291	-1.93871	1.69825	-0.13114	0.89592	0.91629
Bacteriap_Bacteroidetesc_Bacteroidiao_Bacteroidalesf_Muribaculaceaeg_Muribaculaceae_unclassifieds_Muribaculaceae_bacterium_DSM_103720.1	YY-PFMT_YY	0.44149	0.57575	102.36456	-0.70046	1.58344	0.76681	0.44496	0.59328
Bacteriap_Bacteroidetesc_Bacteroidiao_Bacteroidalesf_Muribaculaceaeg_Muribaculaceae_unclassifieds_Muribaculaceae_bacterium_DSM_103720.2	YM-PFMT_YM	1.39157	0.65383	108.69428	0.09566	2.68747	2.12834	0.03557	0.09850
Bacteriap_Bacteroidetesc_Bacteroidiao_Bacteroidalesf_Muribaculaceaeg_Muribaculaceae_unclassifieds_Muribaculaceae_bacterium_DSM_103720.3	OY-PFMT_OY	-0.50171	0.53857	102.36456	-1.56990	0.56649	-0.93156	0.35375	0.53509
Bacteriap_Bacteroidetesc_Bacteroidiao_Bacteroidalesf_Muribaculaceaeg_Muribaculaceae_unclassifieds_Muribaculaceae_bacterium_DSM_103720.4	YO-PFMT_YO	1.35902	0.57575	102.36456	0.21707	2.50097	2.36043	0.02015	0.06053
Bacteriap_Bacteroidetesc_Bacteroidiao_Bacteroidalesf_Muribaculaceaeg_Muribaculums_Muribaculum_intestinale.1	YY-PFMT_YY	1.39154	0.48062	102.24346	0.43826	2.34481	2.89532	0.00463	0.01774
Bacteriap_Bacteroidetesc_Bacteroidiao_Bacteroidalesf_Muribaculaceaeg_Muribaculums_Muribaculum_intestinale.2	YM-PFMT_YM	1.19204	0.54795	107.83188	0.10589	2.27819	2.17546	0.03178	0.09037
Bacteriap_Bacteroidetesc_Bacteroidiao_Bacteroidalesf_Muribaculaceaeg_Muribaculums_Muribaculum_intestinale.3	OY-PFMT_OY	0.80684	0.44958	102.24346	-0.08487	1.69855	1.79467	0.07566	0.16814
Bacteriap_Bacteroidetesc_Bacteroidiao_Bacteroidalesf_Muribaculaceaeg_Muribaculums_Muribaculum_intestinale.4	YO-PFMT_YO	0.37806	0.48062	102.24346	-0.57521	1.33134	0.78662	0.43332	0.58645
Bacteriap_Bacteroidetesc_Bacteroidiao_Bacteroidalesf_Prevotellaceaeg_Prevotellas_Prevotella_sp_MGM1.1	YY-PFMT_YY	0.92896	1.37353	102.23763	-1.79536	3.65329	0.67633	0.50036	0.62982
Bacteriap_Bacteroidetesc_Bacteroidiao_Bacteroidalesf_Prevotellaceaeg_Prevotellas_Prevotella_sp_MGM1.2	YM-PFMT_YM	-4.58637	1.56754	107.57506	-7.69365	-1.47909	-2.92583	0.00419	0.01640
Bacteriap_Bacteroidetesc_Bacteroidiao_Bacteroidalesf_Prevotellaceaeg_Prevotellas_Prevotella_sp_MGM1.3	OY-PFMT_OY	0.34976	1.28482	102.23763	-2.19861	2.89814	0.27223	0.78600	0.84718
Bacteriap_Bacteroidetesc_Bacteroidiao_Bacteroidalesf_Prevotellaceaeg_Prevotellas_Prevotella_sp_MGM1.4	YO-PFMT_YO	0.74963	1.37353	102.23763	-1.97469	3.47396	0.54577	0.58641	0.68990
Bacteriap_Bacteroidetesc_Bacteroidiao_Bacteroidalesf_Prevotellaceaeg_Prevotellas_Prevotella_sp_MGM2.1	YY-PFMT_YY	-0.02598	1.61593	102.36165	-3.23103	3.17907	-0.01608	0.98720	0.98720
Bacteriap_Bacteroidetesc_Bacteroidiao_Bacteroidalesf_Prevotellaceaeg_Prevotellas_Prevotella_sp_MGM2.2	YM-PFMT_YM	-9.29707	1.83512	108.68764	-12.93433	-5.65980	-5.06619	0.00000	0.00001
Bacteriap_Bacteroidetesc_Bacteroidiao_Bacteroidalesf_Prevotellaceaeg_Prevotellas_Prevotella_sp_MGM2.3	OY-PFMT_OY	2.44551	1.51156	102.36165	-0.55254	5.44356	1.61787	0.10877	0.21786
Bacteriap_Bacteroidetesc_Bacteroidiao_Bacteroidalesf_Prevotellaceaeg_Prevotellas_Prevotella_sp_MGM2.4	YO-PFMT_YO	-11.04763	1.61593	102.36165	-14.25268	-7.84258	-6.83671	0.00000	0.00000
Bacteriap_Bacteroidetesc_Bacteroidiao_Bacteroidalesf_Tannerellaceaeg_Parabacteroidess_Parabacteroides_distasonis.1	YY-PFMT_YY	-2.34384	1.17755	102.14258	-4.67947	-0.00820	-1.99043	0.04922	0.12477
Bacteriap_Bacteroidetesc_Bacteroidiao_Bacteroidalesf_Tannerellaceaeg_Parabacteroidess_Parabacteroides_distasonis.2	YM-PFMT_YM	-5.86392	1.34864	106.60477	-8.53755	-3.19029	-4.34803	0.00003	0.00020

Bacteriap_Bacteroidetesc_Bacteroidiao_Bacteroidalesf_Tannerellaceaeg_Parabacteroidess_Parabacteroides_distasonis.3	OY-PFMT_OY	-6.78315	1.10150	102.14258	-8.96794	-4.59837	-6.15810	0.00000	0.00000
Bacteriap_Bacteroidetesc_Bacteroidiao_Bacteroidalesf_Tannerellaceaeg_Parabacteroidess_Parabacteroides_distasonis.4	YO-PFMT_YO	-7.45052	1.17755	102.14258	-9.78616	-5.11489	-6.32711	0.00000	0.00000
Bacteriap_Bacteroidetesc_Bacteroidiao_Bacteroidalesf_Tannerellaceaeg_Parabacteroidess_Parabacteroides_goldsteinii.1	YY-PFMT_YY	-0.21868	0.55305	102.43790	-1.31560	0.87824	-0.39540	0.69337	0.79494
Bacteriap_Bacteroidetesc_Bacteroidiao_Bacteroidalesf_Tannerellaceaeg_Parabacteroidess_Parabacteroides_goldsteinii.2	YM-PFMT_YM	-1.55341	0.62757	108.82913	-2.79725	-0.30958	-2.47530	0.01485	0.04610
Bacteriap_Bacteroidetesc_Bacteroidiao_Bacteroidalesf_Tannerellaceaeg_Parabacteroidess_Parabacteroides_goldsteinii.3	OY-PFMT_OY	-1.56569	0.51733	102.43790	-2.59177	-0.53962	-3.02647	0.00313	0.01280
Bacteriap_Bacteroidetesc_Bacteroidiao_Bacteroidalesf_Tannerellaceaeg_Parabacteroidess_Parabacteroides_goldsteinii.4	YO-PFMT_YO	-0.13037	0.55305	102.43790	-1.22730	0.96655	-0.23574	0.81411	0.86710
Bacteriap_Deferribacteresc_Deferribacteresо_Deferribacteralesf_Deferribacteraceaeg_Mucispirillums_Mucispirillum_schaedleri.1	YY-PFMT_YY	-1.58012	0.83416	102.29519	-3.23462	0.07438	-1.89426	0.06102	0.14129
Bacteriap_Deferribacteresc_Deferribacteresо_Deferribacteralesf_Deferribacteraceaeg_Mucispirillums_Mucispirillum_schaedleri.2	YM-PFMT_YM	0.36447	0.95036	107.98701	-1.51930	2.24825	0.38351	0.70209	0.79985
Bacteriap_Deferribacteresc_Deferribacteresо_Deferribacteralesf_Deferribacteraceaeg_Mucispirillums_Mucispirillum_schaedleri.3	OY-PFMT_OY	1.93947	0.78029	102.29519	0.39182	3.48711	2.48558	0.01455	0.04596
Bacteriap_Deferribacteresc_Deferribacteresо_Deferribacteralesf_Deferribacteraceaeg_Mucispirillums_Mucispirillum_schaedleri.4	YO-PFMT_YO	2.92629	0.83416	102.29519	1.27178	4.58079	3.50805	0.00067	0.00327
Bacteriap_Firmicutesc_Bacillio_Bacillalesf_Staphylococcaceaeg_Staphylococcuss_Staphylococcus_aureus.1	YY-PFMT_YY	3.37800	1.64363	102.43790	0.11804	6.63796	2.05521	0.04240	0.11062
Bacteriap_Firmicutesc_Bacillio_Bacillalesf_Staphylococcaceaeg_Staphylococcuss_Staphylococcus_aureus.2	YM-PFMT_YM	0.90736	1.86507	108.82913	-2.78921	4.60393	0.48650	0.62759	0.72414
Bacteriap_Firmicutesc_Bacillio_Bacillalesf_Staphylococcaceaeg_Staphylococcuss_Staphylococcus_aureus.3	OY-PFMT_OY	0.25901	1.53747	102.43790	-2.79040	3.30842	0.16847	0.86655	0.89643
Bacteriap_Firmicutesc_Bacillio_Bacillalesf_Staphylococcaceaeg_Staphylococcuss_Staphylococcus_aureus.4	YO-PFMT_YO	-1.38975	1.64363	102.43790	-4.64971	1.87021	-0.84554	0.39978	0.56219
Bacteriap_Firmicutesc_Bacillio_Lactobacillalesf_Enterococcaceaeg_Enterococcuss_Enterococcus_faecalis.1	YY-PFMT_YY	0.45637	1.24203	102.41962	-2.00707	2.91981	0.36744	0.71405	0.80331
Bacteriap_Firmicutesc_Bacillio_Lactobacillalesf_Enterococcaceaeg_Enterococcuss_Enterococcus_faecalis.2	YM-PFMT_YM	-2.41391	1.41004	108.73844	-5.20864	0.38082	-1.71194	0.08976	0.19466
Bacteriap_Firmicutesc_Bacillio_Lactobacillalesf_Enterococcaceaeg_Enterococcuss_Enterococcus_faecalis.3	OY-PFMT_OY	-1.02882	1.16181	102.41962	-3.33316	1.27552	-0.88553	0.37795	0.54599
Bacteriap_Firmicutesc_Bacillio_Lactobacillalesf_Enterococcaceaeg_Enterococcuss_Enterococcus_faecalis.4	YO-PFMT_YO	-1.90751	1.24203	102.41962	-4.37095	0.55593	-1.53580	0.12767	0.24448
Bacteriap_Firmicutesc_Bacillio_Lactobacillalesf_Lactobacillaceaeg_Lactobacilluss_Lactobacillus_intestinalis.1	YY-PFMT_YY	0.92896	1.06908	102.43790	-1.19145	3.04938	0.86893	0.38691	0.55273
Bacteriap_Firmicutesc_Bacillio_Lactobacillalesf_Lactobacillaceaeg_Lactobacilluss_Lactobacillus_intestinalis.2	YM-PFMT_YM	-3.46603	1.21312	108.82913	-5.87044	-1.06163	-2.85712	0.00512	0.01899
Bacteriap_Firmicutesc_Bacillio_Lactobacillalesf_Lactobacillaceaeg_Lactobacilluss_Lactobacillus_intestinalis.3	OY-PFMT_OY	8.22024	1.00004	102.43790	6.23678	10.20371	8.21995	0.00000	0.00000
Bacteriap_Firmicutesc_Bacillio_Lactobacillalesf_Lactobacillaceaeg_Lactobacilluss_Lactobacillus_intestinalis.4	YO-PFMT_YO	-6.93508	1.06908	102.43790	-9.05549	-4.81467	-6.48694	0.00000	0.00000
Bacteriap_Firmicutesc_Bacillio_Lactobacillalesf_Lactobacillaceaeg_Lactobacilluss_Lactobacillus_johnsonii.1	YY-PFMT_YY	1.66032	1.67523	102.35503	-1.66236	4.98300	0.99110	0.32397	0.52359
Bacteriap_Firmicutesc_Bacillio_Lactobacillalesf_Lactobacillaceaeg_Lactobacilluss_Lactobacillus_johnsonii.2	YM-PFMT_YM	-10.31757	1.90261	108.67210	-14.08860	-6.54654	-5.42286	0.00000	0.00000
Bacteriap_Firmicutesc_Bacillio_Lactobacillalesf_Lactobacillaceaeg_Lactobacilluss_Lactobacillus_johnsonii.3	OY-PFMT_OY	10.94184	1.56704	102.35503	7.83376	14.04992	6.98250	0.00000	0.00000
Bacteriap_Firmicutesc_Bacillio_Lactobacillalesf_Lactobacillaceaeg_Lactobacilluss_Lactobacillus_johnsonii.4	YO-PFMT_YO	-11.34082	1.67523	102.35503	-14.66350	-8.01814	-6.76970	0.00000	0.00000
Bacteriap_Firmicutesc_Bacillio_Lactobacillalesf_Lactobacillaceaeg_Lactobacilluss_Lactobacillus_murinus.1	YY-PFMT_YY	0.06494	0.82006	102.20339	-1.56160	1.69149	0.07919	0.93703	0.94756
Bacteriap_Firmicutesc_Bacillio_Lactobacillalesf_Lactobacillaceaeg_Lactobacilluss_Lactobacillus_murinus.2	YM-PFMT_YM	1.33619	0.93579	107.59456	-0.51878	3.19117	1.42788	0.15622	0.27842
Bacteriap_Firmicutesc_Bacillio_Lactobacillalesf_Lactobacillaceaeg_Lactobacilluss_Lactobacillus_murinus.3	OY-PFMT_OY	-0.59515	0.76710	102.20339	-2.11665	0.92634	-0.77585	0.43963	0.59055
Bacteriap_Firmicutesc_Bacillio_Lactobacillalesf_Lactobacillaceaeg_Lactobacilluss_Lactobacillus_murinus.4	YO-PFMT_YO	1.83682	0.82006	102.20339	0.21027	3.46336	2.23985	0.02727	0.07916
Bacteriap_Firmicutesc_Bacillio_Lactobacillalesf_Lactobacillaceaeg_Lactobacilluss_Lactobacillus_reuteri.1	YY-PFMT_YY	-0.46199	1.66694	102.37221	-3.76821	2.84424	-0.27715	0.78223	0.84718
Bacteriap_Firmicutesc_Bacillio_Lactobacillalesf_Lactobacillaceaeg_Lactobacilluss_Lactobacillus_reuteri.2	YM-PFMT_YM	1.80401	1.89283	108.71114	-1.94763	5.55566	0.95308	0.34267	0.52718
Bacteriap_Firmicutesc_Bacillio_Lactobacillalesf_Lactobacillaceaeg_Lactobacilluss_Lactobacillus_reuteri.3	OY-PFMT_OY	6.18332	1.55928	102.37221	3.09063	9.27601	3.96550	0.00014	0.00070
Bacteriap_Firmicutesc_Bacillio_Lactobacillalesf_Lactobacillaceaeg_Lactobacilluss_Lactobacillus_reuteri.4	YO-PFMT_YO	0.35097	1.66694	102.37221	-2.95526	3.65719	0.21055	0.83366	0.88245
Bacteriap_Firmicutesc_Bacillio_Lactobacillalesf_Lactobacillaceaeg_Lactobacilluss_Lactobacillus_taiwanensis.1	YY-PFMT_YY	-7.12650	1.95489	102.35386	-11.00385	-3.24914	-3.64547	0.00042	0.00211
Bacteriap_Firmicutesc_Bacillio_Lactobacillalesf_Lactobacillaceaeg_Lactobacilluss_Lactobacillus_taiwanensis.2	YM-PFMT_YM	6.77992	2.22025	108.66929	2.37931	11.18053	3.05368	0.00284	0.01190
Bacteriap_Firmicutesc_Bacillio_Lactobacillalesf_Lactobacillaceaeg_Lactobacilluss_Lactobacillus_taiwanensis.3	OY-PFMT_OY	3.69346	1.82863	102.35386	0.06653	7.32040	2.01979	0.04602	0.11833
Bacteriap_Firmicutesc_Bacillio_Lactobacillalesf_Lactobacillaceaeg_Lactobacilluss_Lactobacillus_taiwanensis.4	YO-PFMT_YO	2.04804	1.95489	102.35386	-1.82932	5.92539	1.04765	0.29727	0.49090
Bacteriap_Firmicutesc_Clostridiao_Clostridialesf_Clostridiaceaeg_Clostridiums_Clostridium_sp_ASF356.1	YY-PFMT_YY	-1.16351	1.67339	102.41525	-4.48251	2.15549	-0.69530	0.48844	0.62164
Bacteriap_Firmicutesc_Clostridiao_Clostridialesf_Clostridiaceaeg_Clostridiums_Clostridium_sp_ASF356.2	YM-PFMT_YM	-1.68353	1.89928	108.79307	-5.44793	2.08087	-0.88640	0.37736	0.54599
Bacteriap_Firmicutesc_Clostridiao_Clostridialesf_Clostridiaceaeg_Clostridiums_Clostridium_sp_ASF356.3	OY-PFMT_OY	-4.07457	1.56531	102.41525	-7.17920	-0.96993	-2.60304	0.01061	0.03673
Bacteriap_Firmicutesc_Clostridiao_Clostridialesf_Clostridiaceaeg_Clostridiums_Clostridium_sp_ASF356.4	YO-PFMT_YO	3.79472	1.67339	102.41525	0.47572	7.11372	2.26768	0.02545	0.07509
Bacteriap_Firmicutesc_Clostridiao_Clostridialesf_Clostridiaceaeg_Clostridiums_Clostridium_sp_ASF502.1	YY-PFMT_YY	1.20418	0.81322	102.39963	-0.40877	2.81713	1.48075	0.14174	0.26857
Bacteriap_Firmicutesc_Clostridiao_Clostridialesf_Clostridiaceaeg_Clostridiums_Clostridium_sp_ASF502.2	YM-PFMT_YM	0.33153	0.92315	108.76560	-1.49818	2.16123	0.35912	0.72020	0.80519
Bacteriap_Firmicutesc_Clostridiao_Clostridialesf_Clostridiaceaeg_Clostridiums_Clostridium_sp_ASF502.3	OY-PFMT_OY	1.18118	0.76070	102.39963	-0.32760	2.68996	1.55275	0.12357	0.23916
Bacteriap_Firmicutesc_Clostridiao_Clostridialesf_Clostridiaceaeg_Clostridiums_Clostridium_sp_ASF502.4	YO-PFMT_YO	1.18036	0.81322	102.39963	-0.43259	2.79331	1.45146	0.14971	0.27220
Bacteriap_Firmicutesc_Clostridiao_Clostridialesf_Eubacteriaceaeg_Eubacteriums_Eubacterium_sp_14_2.1	YY-PFMT_YY	-6.08487	1.19761	102.32527	-8.46023	-3.70950	-5.08084	0.00000	0.00001
Bacteriap_Firmicutesc_Clostridiao_Clostridialesf_Eubacteriaceaeg_Eubacteriums_Eubacterium_sp_14_2.2	YM-PFMT_YM	6.25409	1.36059	108.59327	3.55733	8.95085	4.59659	0.00001	0.00008

Bacteriap_Firmicutesc_Clostridiao_Clostridialesf_Eubacteriaceaeg_Eubacteriums_Eubacterium_sp_14_2.3	OY-PFMT_OY	-9.31177	1.12026	102.32527	-11.53372	-7.08982	-8.31214	0.00000	0.00000
Bacteriap_Firmicutesc_Clostridiao_Clostridialesf_Eubacteriaceaeg_Eubacteriums_Eubacterium_sp_14_2.4	YO-PFMT_YO	1.95208	1.19761	102.32527	-0.42328	4.32744	1.62998	0.10618	0.21786
Bacteriap_Firmicutesc_Clostridiao_Clostridialesf_Lachnospiraceaeg_Acetatifactors_Acetatifactor_muris.1	YY-PFMT_YY	6.27383	1.43082	102.22634	3.43588	9.11178	4.38478	0.00003	0.00019
Bacteriap_Firmicutesc_Clostridiao_Clostridialesf_Lachnospiraceaeg_Acetatifactors_Acetatifactor_muris.2	YM-PFMT_YM	7.07467	1.63302	107.56461	3.83759	10.31175	4.33226	0.00003	0.00021
Bacteriap_Firmicutesc_Clostridiao_Clostridialesf_Lachnospiraceaeg_Acetatifactors_Acetatifactor_muris.3	OY-PFMT_OY	3.15860	1.33841	102.22634	0.50394	5.81325	2.35997	0.02018	0.06053
Bacteriap_Firmicutesc_Clostridiao_Clostridialesf_Lachnospiraceaeg_Acetatifactors_Acetatifactor_muris.4	YO-PFMT_YO	2.70189	1.43082	102.22634	-0.13606	5.53984	1.88835	0.06181	0.14129
Bacteriap_Firmicutesc_Clostridiao_Clostridialesf_Lachnospiraceaeg_Doreas_Dorea_sp_5_2.1	YY-PFMT_YY	0.32893	0.56884	102.36917	-0.79931	1.45718	0.57825	0.56437	0.67434
Bacteriap_Firmicutesc_Clostridiao_Clostridialesf_Lachnospiraceaeg_Doreas_Dorea_sp_5_2.2	YM-PFMT_YM	-0.61121	0.64595	108.70452	-1.89150	0.66908	-0.94622	0.34613	0.52800
Bacteriap_Firmicutesc_Clostridiao_Clostridialesf_Lachnospiraceaeg_Doreas_Dorea_sp_5_2.3	OY-PFMT_OY	0.84685	0.53210	102.36917	-0.20852	1.90223	1.59152	0.11458	0.22663
Bacteriap_Firmicutesc_Clostridiao_Clostridialesf_Lachnospiraceaeg_Doreas_Dorea_sp_5_2.4	YO-PFMT_YO	-0.68342	0.56884	102.36917	-1.81167	0.44482	-1.20143	0.23236	0.39457
Bacteriap_Firmicutesc_Clostridiao_Clostridialesf_Lachnospiraceaeg_Lachnospiraceae_unclassifieds_Lachnospiraceae_bacterium_10_1.1	YY-PFMT_YY	3.68337	2.16155	102.30666	-0.60391	7.97065	1.70404	0.09141	0.19588
Bacteriap_Firmicutesc_Clostridiao_Clostridialesf_Lachnospiraceaeg_Lachnospiraceae_unclassifieds_Lachnospiraceae_bacterium_10_1.2	YM-PFMT_YM	-1.42626	2.45811	108.40307	-6.29846	3.44594	-0.58023	0.56297	0.67434
Bacteriap_Firmicutesc_Clostridiao_Clostridialesf_Lachnospiraceaeg_Lachnospiraceae_unclassifieds_Lachnospiraceae_bacterium_10_1.3	OY-PFMT_OY	1.80798	2.02195	102.30666	-2.20240	5.81837	0.89418	0.37332	0.54599
Bacteriap_Firmicutesc_Clostridiao_Clostridialesf_Lachnospiraceaeg_Lachnospiraceae_unclassifieds_Lachnospiraceae_bacterium_10_1.4	YO-PFMT_YO	-7.33665	2.16155	102.30666	-11.62392	-3.04937	-3.39415	0.00098	0.00453
Bacteriap_Firmicutesc_Clostridiao_Clostridialesf_Lachnospiraceaeg_Lachnospiraceae_unclassifieds_Lachnospiraceae_bacterium_28_4.1	YY-PFMT_YY	0.59781	1.62633	102.43790	-2.62784	3.82346	0.36758	0.71394	0.80331
Bacteriap_Firmicutesc_Clostridiao_Clostridialesf_Lachnospiraceaeg_Lachnospiraceae_unclassifieds_Lachnospiraceae_bacterium_28_4.2	YM-PFMT_YM	-2.66425	1.84544	108.82913	-6.32191	0.99342	-1.44369	0.15170	0.27306
Bacteriap_Firmicutesc_Clostridiao_Clostridialesf_Lachnospiraceaeg_Lachnospiraceae_unclassifieds_Lachnospiraceae_bacterium_28_4.3	OY-PFMT_OY	-6.56637	1.52129	102.43790	-9.58369	-3.54906	-4.31632	0.00004	0.00022
Bacteriap_Firmicutesc_Clostridiao_Clostridialesf_Lachnospiraceaeg_Lachnospiraceae_unclassifieds_Lachnospiraceae_bacterium_28_4.4	YO-PFMT_YO	-0.42351	1.62633	102.43790	-3.64916	2.80214	-0.26041	0.79507	0.85186
Bacteriap_Firmicutesc_Clostridiao_Clostridialesf_Lachnospiraceaeg_Lachnospiraceae_unclassifieds_Lachnospiraceae_bacterium_3_1.1	YY-PFMT_YY	0.09866	0.79598	102.20927	-1.48012	1.67743	0.12395	0.90160	0.91688
Bacteriap_Firmicutesc_Clostridiao_Clostridialesf_Lachnospiraceaeg_Lachnospiraceae_unclassifieds_Lachnospiraceae_bacterium_3_1.2	YM-PFMT_YM	3.74876	0.90861	107.52264	1.94764	5.54988	4.12580	0.00007	0.00041
Bacteriap_Firmicutesc_Clostridiao_Clostridialesf_Lachnospiraceaeg_Lachnospiraceae_unclassifieds_Lachnospiraceae_bacterium_3_1.3	OY-PFMT_OY	0.10528	0.74457	102.20927	-1.37153	1.58209	0.14139	0.88784	0.91320
Bacteriap_Firmicutesc_Clostridiao_Clostridialesf_Lachnospiraceaeg_Lachnospiraceae_unclassifieds_Lachnospiraceae_bacterium_3_1.4	YO-PFMT_YO	1.67599	0.79598	102.20927	0.09722	3.25477	2.10558	0.03769	0.10279
Bacteriap_Firmicutesc_Clostridiao_Clostridialesf_Lachnospiraceaeg_Lachnospiraceae_unclassifieds_Lachnospiraceae_bacterium_3_2.1	YY-PFMT_YY	3.29691	1.57674	102.42723	0.16961	6.42421	2.09097	0.03901	0.10479
Bacteriap_Firmicutesc_Clostridiao_Clostridialesf_Lachnospiraceaeg_Lachnospiraceae_unclassifieds_Lachnospiraceae_bacterium_3_2.2	YM-PFMT_YM	-0.36603	1.78967	108.77669	-3.91317	3.18111	-0.20452	0.83833	0.88245
Bacteriap_Firmicutesc_Clostridiao_Clostridialesf_Lachnospiraceaeg_Lachnospiraceae_unclassifieds_Lachnospiraceae_bacterium_3_2.3	OY-PFMT_OY	1.45136	1.47490	102.42723	-1.47395	4.37668	0.98404	0.32741	0.52359
Bacteriap_Firmicutesc_Clostridiao_Clostridialesf_Lachnospiraceaeg_Lachnospiraceae_unclassifieds_Lachnospiraceae_bacterium_3_2.4	YO-PFMT_YO	1.66138	1.57674	102.42723	-1.46592	4.78868	1.05368	0.29451	0.49085
Bacteriap_Firmicutesc_Clostridiao_Clostridialesf_Lachnospiraceaeg_Lachnospiraceae_unclassifieds_Lachnospiraceae_bacterium_A2.1	YY-PFMT_YY	3.19066	1.11648	102.38355	0.97623	5.40508	2.85779	0.00517	0.01899
Bacteriap_Firmicutesc_Clostridiao_Clostridialesf_Lachnospiraceaeg_Lachnospiraceae_unclassifieds_Lachnospiraceae_bacterium_A2.2	YM-PFMT_YM	1.36646	1.26762	108.73473	-1.14599	3.87890	1.07797	0.28343	0.47680
Bacteriap_Firmicutesc_Clostridiao_Clostridialesf_Lachnospiraceaeg_Lachnospiraceae_unclassifieds_Lachnospiraceae_bacterium_A2.3	OY-PFMT_OY	1.77020	1.04437	102.38355	-0.30121	3.84160	1.69499	0.09312	0.19719
Bacteriap_Firmicutesc_Clostridiao_Clostridialesf_Lachnospiraceaeg_Lachnospiraceae_unclassifieds_Lachnospiraceae_bacterium_A2.4	YO-PFMT_YO	1.80547	1.11648	102.38355	-0.40896	4.01989	1.61711	0.10893	0.21786
Bacteriap_Firmicutesc_Clostridiao_Clostridialesf_Lachnospiraceaeg_Lachnospiraceae_unclassifieds_Lachnospiraceae_bacterium_A4.1	YY-PFMT_YY	1.41058	0.73217	102.14691	-0.04166	2.86281	1.92657	0.05681	0.13635
Bacteriap_Firmicutesc_Clostridiao_Clostridialesf_Lachnospiraceaeg_Lachnospiraceae_unclassifieds_Lachnospiraceae_bacterium_A4.2	YM-PFMT_YM	1.21652	0.83770	106.89492	-0.44414	2.87718	1.45222	0.14937	0.27220
Bacteriap_Firmicutesc_Clostridiao_Clostridialesf_Lachnospiraceaeg_Lachnospiraceae_unclassifieds_Lachnospiraceae_bacterium_A4.3	OY-PFMT_OY	2.94413	0.68488	102.14691	1.58569	4.30257	4.29872	0.00004	0.00023
Bacteriap_Firmicutesc_Clostridiao_Clostridialesf_Lachnospiraceaeg_Lachnospiraceae_unclassifieds_Lachnospiraceae_bacterium_A4.4	YO-PFMT_YO	0.65076	0.73217	102.14691	-0.80147	2.10300	0.88881	0.37619	0.54599
Bacteriap_Firmicutesc_Clostridiao_Clostridialesf_Lachnospiraceaeg_Lachnospiraceae_unclassifieds_Lachnospiraceae_bacterium_COE1.1	YY-PFMT_YY	2.83145	1.94030	102.38103	-1.01695	6.67986	1.45929	0.14755	0.27220
Bacteriap_Firmicutesc_Clostridiao_Clostridialesf_Lachnospiraceaeg_Lachnospiraceae_unclassifieds_Lachnospiraceae_bacterium_COE1.2	YM-PFMT_YM	5.87168	2.20303	108.72962	1.50523	10.23813	2.66528	0.00886	0.03129
Bacteriap_Firmicutesc_Clostridiao_Clostridialesf_Lachnospiraceaeg_Lachnospiraceae_unclassifieds_Lachnospiraceae_bacterium_COE1.3	OY-PFMT_OY	-0.06072	1.81498	102.38103	-3.66058	3.53913	-0.03346	0.97337	0.97881
Bacteriap_Firmicutesc_Clostridiao_Clostridialesf_Lachnospiraceaeg_Lachnospiraceae_unclassifieds_Lachnospiraceae_bacterium_COE1.4	YO-PFMT_YO	1.65189	1.94030	102.38103	-2.19651	5.50030	0.85136	0.39656	0.56205
Bacteriap_Firmicutesc_Clostridiao_Clostridialesf_Lachnospiraceaeg_Lachnospiraceae_unclassifieds_Lachnospiraceae_bacterium_M18_1.1	YY-PFMT_YY	3.53295	1.38485	102.36719	0.78621	6.27968	2.55113	0.01222	0.04072
Bacteriap_Firmicutesc_Clostridiao_Clostridialesf_Lachnospiraceaeg_Lachnospiraceae_unclassifieds_Lachnospiraceae_bacterium_M18_1.2	YM-PFMT_YM	-0.51584	1.57261	108.70017	-3.63279	2.60112	-0.32801	0.74353	0.82615
Bacteriap_Firmicutesc_Clostridiao_Clostridialesf_Lachnospiraceaeg_Lachnospiraceae_unclassifieds_Lachnospiraceae_bacterium_M18_1.3	OY-PFMT_OY	2.56819	1.29541	102.36719	-0.00115	5.13752	1.98253	0.05010	0.12525
Bacteriap_Firmicutesc_Clostridiao_Clostridialesf_Lachnospiraceaeg_Lachnospiraceae_unclassifieds_Lachnospiraceae_bacterium_M18_1.4	YO-PFMT_YO	0.79805	1.38485	102.36719	-1.94869	3.54478	0.57627	0.56570	0.67434
Bacteriap_Firmicutesc_Clostridiao_Clostridialesf_Oscillospiraceaeg_Oscillibacters_Oscillibacter_sp_1_3.1	YY-PFMT_YY	6.43776	1.21101	102.39127	4.03583	8.83968	5.31602	0.00000	0.00001
Bacteriap_Firmicutesc_Clostridiao_Clostridialesf_Oscillospiraceaeg_Oscillibacters_Oscillibacter_sp_1_3.2	YM-PFMT_YM	4.22501	1.37484	108.74990	1.50006	6.94996	3.07310	0.00268	0.01148
Bacteriap_Firmicutesc_Clostridiao_Clostridialesf_Oscillospiraceaeg_Oscillibacters_Oscillibacter_sp_1_3.3	OY-PFMT_OY	4.65030	1.13280	102.39127	2.40351	6.89709	4.10515	0.00008	0.00044
Bacteriap_Firmicutesc_Clostridiao_Clostridialesf_Oscillospiraceaeg_Oscillibacters_Oscillibacter_sp_1_3.4	YO-PFMT_YO	0.86122	1.21101	102.39127	-1.54070	3.26314	0.71116	0.47860	0.61535
Bacteriap_Firmicutesc_Clostridiao_Clostridialesf_Ruminococcaceaeg_Acutalibacters_Acutalibacter_muris.1	YY-PFMT_YY	4.60262	1.45627	102.24105	1.71419	7.49104	3.16055	0.00207	0.00910
Bacteriap_Firmicutesc_Clostridiao_Clostridialesf_Ruminococcaceaeg_Acutalibacters_Acutalibacter_muris.2	YM-PFMT_YM	6.74396	1.66202	107.56223	3.44939	10.03853	4.05768	0.00009	0.00050

Bacteriap_Firmicutesc_Clostridiao_Clostridialesf_Ruminococcaceaeg_Acutalibacters_Acutalibacter_muris.3	OY-PFMT_OY	8.75126	1.36222	102.24105	6.04939	11.45314	6.42428	0.00000	0.00000
Bacteriap_Firmicutesc_Clostridiao_Clostridialesf_Ruminococcaceaeg_Acutalibacters_Acutalibacter_muris.4	YO-PFMT_YO	2.36695	1.45627	102.24105	-0.52147	5.25538	1.62535	0.10717	0.21786
Bacteriap_Firmicutesc_Clostridiao_Clostridialesf_Ruminococcaceaeg_Anaerotruncuss_Anaerotruncus_sp_G3_2012.1	YY-PFMT_YY	0.92924	0.42774	102.24606	0.08085	1.77763	2.17245	0.03213	0.09037
Bacteriap_Firmicutesc_Clostridiao_Clostridialesf_Ruminococcaceaeg_Anaerotruncuss_Anaerotruncus_sp_G3_2012.2	YM-PFMT_YM	1.21918	0.48771	107.80820	0.25244	2.18593	2.49981	0.01393	0.04478
Bacteriap_Firmicutesc_Clostridiao_Clostridialesf_Ruminococcaceaeg_Anaerotruncuss_Anaerotruncus_sp_G3_2012.3	OY-PFMT_OY	0.82567	0.40011	102.24606	0.03208	1.61927	2.06361	0.04159	0.11008
Bacteriap_Firmicutesc_Clostridiao_Clostridialesf_Ruminococcaceaeg_Anaerotruncuss_Anaerotruncus_sp_G3_2012.4	YO-PFMT_YO	1.40706	0.42774	102.24606	0.55868	2.25545	3.28956	0.00138	0.00620
Bacteriap_Firmicutesc_Erysipelotrichiao_Erysipelotrichalesf_Erysipelotrichaceaeg_Erysipelatoclostridiums_Clostridium_cocleatum.1	YY-PFMT_YY	-7.31872	1.27241	102.43101	-9.84241	-4.79503	-5.75186	0.00000	0.00000
Bacteriap_Firmicutesc_Erysipelotrichiao_Erysipelotrichalesf_Erysipelotrichaceaeg_Erysipelatoclostridiums_Clostridium_cocleatum.2	YM-PFMT_YM	-3.71724	1.44394	108.81859	-6.57913	-0.85534	-2.57437	0.01139	0.03867
Bacteriap_Firmicutesc_Erysipelotrichiao_Erysipelotrichalesf_Erysipelotrichaceaeg_Erysipelatoclostridiums_Clostridium_cocleatum.3	OY-PFMT_OY	-7.14384	1.19023	102.43101	-9.50454	-4.78315	-6.00207	0.00000	0.00000
Bacteriap_Firmicutesc_Erysipelotrichiao_Erysipelotrichalesf_Erysipelotrichaceaeg_Erysipelatoclostridiums_Clostridium_cocleatum.4	YO-PFMT_YO	0.74963	1.27241	102.43101	-1.77406	3.27332	0.58914	0.55706	0.67434
Bacteriap_Firmicutesc_Erysipelotrichiao_Erysipelotrichalesf_Erysipelotrichaceaeg_Faecalibaculums_Faecalibaculum_rodentium.1	YY-PFMT_YY	0.86159	0.85012	102.36286	-0.82454	2.54773	1.01350	0.31321	0.51253
Bacteriap_Firmicutesc_Erysipelotrichiao_Erysipelotrichalesf_Erysipelotrichaceaeg_Faecalibaculums_Faecalibaculum_rodentium.2	YM-PFMT_YM	-3.32082	0.96542	108.69042	-5.23431	-1.40733	-3.43977	0.00083	0.00392
Bacteriap_Firmicutesc_Erysipelotrichiao_Erysipelotrichalesf_Erysipelotrichaceaeg_Faecalibaculums_Faecalibaculum_rodentium.3	OY-PFMT_OY	-0.15492	0.79521	102.36286	-1.73216	1.42231	-0.19482	0.84592	0.88527
Bacteriap_Firmicutesc_Erysipelotrichiao_Erysipelotrichalesf_Erysipelotrichaceaeg_Faecalibaculums_Faecalibaculum_rodentium.4	YO-PFMT_YO	-6.73171	0.85012	102.36286	-8.41785	-5.04558	-7.91856	0.00000	0.00000
Bacteriap_Firmicutesc_Firmicutes_unclassifiedo_Firmicutes_unclassifiedf_Firmicutes_unclassifiedg_Firmicutes_unclassifieds_Firmicutes_bacterium_ASF500.1	YY-PFMT_YY	1.57348	0.82176	102.35066	-0.05642	3.20337	1.91476	0.05832	0.13812
Bacteriap_Firmicutesc_Firmicutes_unclassifiedo_Firmicutes_unclassifiedf_Firmicutes_unclassifiedg_Firmicutes_unclassifieds_Firmicutes_bacterium_ASF500.2	YM-PFMT_YM	4.66746	0.93334	108.66148	2.81754	6.51737	5.00081	0.00000	0.00002
Bacteriap_Firmicutesc_Firmicutes_unclassifiedo_Firmicutes_unclassifiedf_Firmicutes_unclassifiedg_Firmicutes_unclassifieds_Firmicutes_bacterium_ASF500.3	OY-PFMT_OY	3.77318	0.76869	102.35066	2.24855	5.29780	4.90859	0.00000	0.00003
Bacteriap_Firmicutesc_Firmicutes_unclassifiedo_Firmicutes_unclassifiedf_Firmicutes_unclassifiedg_Firmicutes_unclassifieds_Firmicutes_bacterium_ASF500.4	YO-PFMT_YO	1.58871	0.82176	102.35066	-0.04118	3.21861	1.93330	0.05596	0.13612
Bacteriap_Proteobacteriac_Betaproteobacteriao_Burkholderialesf_Sutterellaceaeg_Parasutterellas_Parasutterella_excrementihominis.1	YY-PFMT_YY	-0.86441	1.55537	102.33433	-3.94935	2.22053	-0.55576	0.57959	0.68635
Bacteriap_Proteobacteriac_Betaproteobacteriao_Burkholderialesf_Sutterellaceaeg_Parasutterellas_Parasutterella_excrementihominis.2	YM-PFMT_YM	3.32048	1.76696	108.60974	-0.18172	6.82268	1.87921	0.06290	0.14152
Bacteriap_Proteobacteriac_Betaproteobacteriao_Burkholderialesf_Sutterellaceaeg_Parasutterellas_Parasutterella_excrementihominis.3	OY-PFMT_OY	-2.30559	1.45491	102.33433	-5.19129	0.58011	-1.58470	0.11612	0.22719
Bacteriap_Proteobacteriac_Betaproteobacteriao_Burkholderialesf_Sutterellaceaeg_Parasutterellas_Parasutterella_excrementihominis.4	YO-PFMT_YO	9.34885	1.55537	102.33433	6.26391	12.43379	6.01071	0.00000	0.00000
Bacteriap_Proteobacteriac_Betaproteobacteriao_Burkholderialesf_Sutterellaceaeg_Turicimonass_Turicimonas_muris.1	YY-PFMT_YY	0.59166	0.93044	102.34829	-1.25379	2.43710	0.63589	0.52627	0.65783
Bacteriap_Proteobacteriac_Betaproteobacteriao_Burkholderialesf_Sutterellaceaeg_Turicimonass_Turicimonas_muris.2	YM-PFMT_YM	0.87671	1.05680	108.65560	-1.21791	2.97133	0.82959	0.40859	0.56574
Bacteriap_Proteobacteriac_Betaproteobacteriao_Burkholderialesf_Sutterellaceaeg_Turicimonass_Turicimonas_muris.3	OY-PFMT_OY	-2.40623	0.87035	102.34829	-4.13249	-0.67998	-2.76469	0.00676	0.02433
Bacteriap_Proteobacteriac_Betaproteobacteriao_Burkholderialesf_Sutterellaceaeg_Turicimonass_Turicimonas_muris.4	YO-PFMT_YO	0.16747	0.93044	102.34829	-1.67798	2.01291	0.17999	0.85752	0.89222
Bacteriap_Proteobacteriac_Gammaproteobacteriao_Enterobacteralesf_Enterobacteriaceaeg_Escherichias_Escherichia_coli.1	YY-PFMT_YY	-2.05837	1.45636	102.43790	-4.94690	0.83016	-1.41337	0.16058	0.28030
Bacteriap_Proteobacteriac_Gammaproteobacteriao_Enterobacteralesf_Enterobacteriaceaeg_Escherichias_Escherichia_coli.2	YM-PFMT_YM	-2.41269	1.65257	108.82913	-5.68809	0.86271	-1.45996	0.14718	0.27220
Bacteriap_Proteobacteriac_Gammaproteobacteriao_Enterobacteralesf_Enterobacteriaceaeg_Escherichias_Escherichia_coli.3	OY-PFMT_OY	-0.97976	1.36230	102.43790	-3.68173	1.72222	-0.71919	0.47366	0.61535
Bacteriap_Proteobacteriac_Gammaproteobacteriao_Enterobacteralesf_Enterobacteriaceaeg_Escherichias_Escherichia_coli.4	YO-PFMT_YO	2.05157	1.45636	102.43790	-0.83696	4.94010	1.40870	0.16195	0.28030
Bacteriap_Proteobacteriac_Proteobacteria_unclassifiedo_Proteobacteria_unclassifiedf_Proteobacteria_unclassifiedg_Proteobacteria_unclassifieds_Proteobacteria_bacterium_CAG_139.1	YY-PFMT_YY	0.92896	1.16183	102.26320	-1.37545	3.23338	0.79957	0.42581	0.58066
Bacteriap_Proteobacteriac_Proteobacteria_unclassifiedo_Proteobacteria_unclassifiedf_Proteobacteria_unclassifiedg_Proteobacteria_unclassifieds_Proteobacteria_bacterium_CAG_139.2	YM-PFMT_YM	0.71297	1.32285	108.12777	-1.90911	3.33505	0.53896	0.59102	0.69080
Bacteriap_Proteobacteriac_Proteobacteria_unclassifiedo_Proteobacteria_unclassifiedf_Proteobacteria_unclassifiedg_Proteobacteria_unclassifieds_Proteobacteria_bacterium_CAG_139.3	OY-PFMT_OY	0.34976	1.08679	102.26320	-1.80582	2.50535	0.32183	0.74824	0.82627
Bacteriap_Proteobacteriac_Proteobacteria_unclassifiedo_Proteobacteria_unclassifiedf_Proteobacteria_unclassifiedg_Proteobacteria_unclassifieds_Proteobacteria_bacterium_CAG_139.4	YO-PFMT_YO	5.69362	1.16183	102.26320	3.38920	7.99804	4.90055	0.00000	0.00003
Bacteriap_Verrucomicrobiac_Verrucomicrobiaeo_Verrucomicrobialesf_Akkermansiaceaeg_Akkermansias_Akkermansia_muciniphila.1	YY-PFMT_YY	-10.42237	1.51209	102.34196	-13.42147	-7.42327	-6.89270	0.00000	0.00000
Bacteriap_Verrucomicrobiac_Verrucomicrobiaeo_Verrucomicrobialesf_Akkermansiaceaeg_Akkermansias_Akkermansia_muciniphila.2	YM-PFMT_YM	-5.03856	1.71756	108.63941	-8.44284	-1.63428	-2.93355	0.00409	0.01635
Bacteriap_Verrucomicrobiac_Verrucomicrobiaeo_Verrucomicrobialesf_Akkermansiaceaeg_Akkermansias_Akkermansia_muciniphila.3	OY-PFMT_OY	-7.68583	1.41443	102.34196	-10.49123	-4.88043	-5.43387	0.00000	0.00000
Bacteriap_Verrucomicrobiac_Verrucomicrobiaeo_Verrucomicrobialesf_Akkermansiaceaeg_Akkermansias_Akkermansia_muciniphila.4	YO-PFMT_YO	-0.91228	1.51209	102.34196	-3.91138	2.08682	-0.60332	0.54763	0.67434
