# Supplementary material for: Fecal microbiota transfer between young and aged mice reverses hallmarks of the aging gut, eye, and brain
Source: Microbiome. 2022 Apr 29;10:68. doi: 10.1186/s40168-022-01243-w (PMC9063061; doi:10.1186/s40168-022-01243-w)
Supplement: Supplementary file 3 — Additional file 2: Table S2. Differential abundance table (family). Relates to Figure S3A. Provided as a separate Excel file. [file 40168_2022_1243_MOESM3_ESM.pdf]

Table S2. Differential adundance table (family).

Post FMT vs. Pre Abx. Groups: S_OY = Aged Pre Abx; PMFT_OY = Aged Post-FMT. S_YY = Young Pre Abx, PFMT YY = Young Post-FMT.

Species	contrast	estimate	SE	df	lower.CL	upper.CL	t.ratio	p.value	padjust	ransformatio
|p_Firmicutes|c_Clostridia|o_Clostridiales|f_Ruminococcaceae	YO-PFMT_YO	2.13E-01	8.32E-02	1.00E+02	4.80E-02	3.78E-01	2.56E+00	1.19E-02	4.34E-02	ArcSin
|p_Bacteroidetes|c_Bacteroidia|o_Bacteroidales|f_Bacteroidaceae	YY-PFMT_YY	-1.87E+00	5.57E-01	1.02E+02	-2.98E+00	-7.64E-01	-3.36E+00	1.11E-03	3.90E-03	Log
|p_Bacteroidetes|c_Bacteroidia|o_Bacteroidales|f_Bacteroidaceae	OY-PFMT_OY	-3.34E+00	5.21E-01	1.02E+02	-4.37E+00	-2.30E+00	-6.40E+00	4.69E-09	4.37E-08	Log
|p_Actino|c_Actino|o_Bifidoles|f_Bifidoceae	YY-PFMT_YY	-1.17E+01	1.47E+00	1.02E+02	-1.46E+01	-8.76E+00	-7.94E+00	2.70E-12	7.55E-11	CLR
|p_Actino|c_Actino|o_Bifidoles|f_Bifidoceae	OY-PFMT_OY	-1.15E+01	1.38E+00	1.02E+02	-1.42E+01	-8.76E+00	-8.35E+00	3.47E-13	1.46E-11	CLR
|p_Actino|c_Coriobacteriia|o_Eggerthellales|f_Eggerthellaceae	YY-PFMT_YY	1.18E+00	3.32E-01	1.02E+02	5.23E-01	1.84E+00	3.56E+00	5.68E-04	1.70E-03	CLR
|p_Actino|c_Coriobacteriia|o_Eggerthellales|f_Eggerthellaceae	OY-PFMT_OY	1.05E+00	3.10E-01	1.02E+02	4.33E-01	1.66E+00	3.38E+00	1.03E-03	2.99E-03	CLR
|p_Bacteroidetes|c_Bacteroidia|o_Bacteroidales|f_Muribaculaceae	YY-PFMT_YY	1.54E+00	4.59E-01	1.02E+02	6.28E-01	2.45E+00	3.35E+00	1.13E-03	3.15E-03	CLR
|p_Bacteroidetes|c_Bacteroidia|o_Bacteroidales|f_Muribaculaceae	OY-PFMT_OY	1.01E+00	4.29E-01	1.02E+02	1.61E-01	1.86E+00	2.36E+00	2.02E-02	4.72E-02	CLR
|p_Bacteroidetes|c_Bacteroidia|o_Bacteroidales|f_Prevotellaceae	YO-PFMT_YO	-1.10E+01	1.67E+00	1.02E+02	-1.43E+01	-7.66E+00	-6.58E+00	2.02E-09	3.39E-08	CLR
|p_Bacteroidetes|c_Bacteroidia|o_Bacteroidales|f_Tannerellaceae	YY-PFMT_YY	-1.08E+00	4.19E-01	1.02E+02	-1.91E+00	-2.47E-01	-2.57E+00	1.15E-02	2.76E-02	CLR
|p_Bacteroidetes|c_Bacteroidia|o_Bacteroidales|f_Tannerellaceae	OY-PFMT_OY	-2.33E+00	3.92E-01	1.02E+02	-3.11E+00	-1.55E+00	-5.95E+00	3.77E-08	3.96E-07	CLR
|p_Deferribacteres|c_Deferribacteres|o_Deferribacterales|f_Deferribacteraceae	OY-PFMT_OY	3.08E+00	7.76E-01	1.02E+02	1.54E+00	4.62E+00	3.97E+00	1.32E-04	4.97E-04	CLR
|p_Deferribacteres|c_Deferribacteres|o_Deferribacterales|f_Deferribacteraceae	YO-PFMT_YO	3.00E+00	8.29E-01	1.02E+02	1.36E+00	4.65E+00	3.62E+00	4.55E-04	1.47E-03	CLR
|p_Firmicutes|c_Clostridia|o_Clostridiales|f_Clostridiaceae	YY-PFMT_YY	2.01E+00	5.59E-01	1.02E+02	9.05E-01	3.12E+00	3.60E+00	4.87E-04	1.52E-03	CLR
|p_Firmicutes|c_Clostridia|o_Clostridiales|f_Clostridiaceae	OY-PFMT_OY	2.12E+00	5.23E-01	1.02E+02	1.08E+00	3.15E+00	4.05E+00	9.85E-05	4.14E-04	CLR
|p_Firmicutes|c_Clostridia|o_Clostridiales|f_Clostridiaceae	YO-PFMT_YO	1.30E+00	5.59E-01	1.02E+02	1.97E-01	2.41E+00	2.34E+00	2.14E-02	4.87E-02	CLR
|p_Firmicutes|c_Clostridia|o_Clostridiales|f_Euceae	YY-PFMT_YY	-5.23E+00	1.17E+00	1.02E+02	-7.55E+00	-2.91E+00	-4.48E+00	1.97E-05	8.71E-05	CLR
|p_Firmicutes|c_Clostridia|o_Clostridiales|f_Euceae	OY-PFMT_OY	-8.17E+00	1.09E+00	1.02E+02	-1.03E+01	-6.00E+00	-7.48E+00	2.67E-11	5.61E-10	CLR
|p_Firmicutes|c_Clostridia|o_Clostridiales|f_Lachnospiraceae	YY-PFMT_YY	2.38E+00	4.90E-01	1.02E+02	1.41E+00	3.35E+00	4.85E+00	4.34E-06	2.18E-05	CLR
|p_Firmicutes|c_Clostridia|o_Clostridiales|f_Lachnospiraceae	OY-PFMT_OY	2.67E+00	4.58E-01	1.02E+02	1.76E+00	3.58E+00	5.84E+00	6.35E-08	5.93E-07	CLR
|p_Firmicutes|c_Clostridia|o_Clostridiales|f_Oscillospiraceae	YY-PFMT_YY	7.29E+00	1.27E+00	1.02E+02	4.78E+00	9.81E+00	5.75E+00	9.12E-08	7.66E-07	CLR
|p_Firmicutes|c_Clostridia|o_Clostridiales|f_Oscillospiraceae	OY-PFMT_OY	5.79E+00	1.19E+00	1.02E+02	3.44E+00	8.14E+00	4.89E+00	3.83E-06	2.14E-05	CLR
|p_Proteo|c_Proteo_unclassified|o_Proteo_unclassified|f_Proteo_unclassified	YO-PFMT_YO	5.77E+00	1.13E+00	1.02E+02	3.52E+00	8.02E+00	5.09E+00	1.61E-06	1.04E-05	CLR
|p_Verrucomicrobia|c_Verrucomicrobiae|o_Verrucomicrobiales|f_Akkermansiaceae	YY-PFMT_YY	-9.57E+00	1.47E+00	1.02E+02	-1.25E+01	-6.65E+00	-6.50E+00	2.95E-09	4.12E-08	CLR
|p_Verrucomicrobia|c_Verrucomicrobiae|o_Verrucomicrobiales|f_Akkermansiaceae	OY-PFMT_OY	-6.54E+00	1.38E+00	1.02E+02	-9.27E+00	-3.81E+00	-4.75E+00	6.53E-06	3.05E-05	CLR
|p_Firmicutes|c_Bacilli|o_Lactobacillales|f_Lactobacillaceae	YY-PFMT_YY	-2.10E+00	6.67E-01	1.02E+02	-3.42E+00	-7.77E-01	-3.15E+00	2.15E-03	1.13E-02	Log_zero
|p_Firmicutes|c_Erysipelotrichia|o_Erysipelotrichales|f_Erysipelotrichaceae	YO-PFMT_YO	-7.95E+00	1.07E+00	1.02E+02	-1.01E+01	-5.82E+00	-7.41E+00	3.73E-11	1.57E-09	Log_zero
|p_Firmicutes|c_Firmicutes_unclassified|o_Firmicutes_unclassified|f_Firmicutes_unclassified	OY-PFMT_OY	4.94E+00	1.02E+00	1.02E+02	2.92E+00	6.95E+00	4.86E+00	4.18E-06	3.90E-05	Log_zero
