# Supplementary material for: Fecal microbiota transfer between young and aged mice reverses hallmarks of the aging gut, eye, and brain
Source: Microbiome. 2022 Apr 29;10:68. doi: 10.1186/s40168-022-01243-w (PMC9063061; doi:10.1186/s40168-022-01243-w)
Supplement: Supplementary file 4 — Additional file 3: Table S3. Raw fecal metabolite concentrations from NMR analysis. Relates to Figure S4. Provided as a separate Excel table. [file 40168_2022_1243_MOESM4_ESM.pdf]

Table S3. Raw metabolite concentrations from NMR analysis

RAW metabolite concentrations (mM.kg-1)

Age	Young	Young	Young	Young	Young	Young	Young	Young	Young
Timepoint	Pre Abx	Pre Abx	Pre Abx	Pre Abx	Pre Abx	Pre Abx	Pre Abx	Post FMT	Post FMT
Ethanol	41.844	63.5166	40.7656	38.2291	41.5772	38.5548	32.8597	56.9137	43.0271
Methanol	3.2521	6.1410	3.6632	2.4511	2.0960	4.0370	1.9865	4.9635	3.0999
Aspartate	10.351	14.0415	4.2613	6.1437	1.8476	1.1081	1.5593	12.2900	2.7394
Alanine	2.0799	11.8468	2.7608	2.0239	1.4632	2.2241	1.3670	4.1385	1.8290
Citrulline	0.0000	8.6027	1.7195	0.0000	2.0506	2.2562	1.8877	2.7074	2.7795
Lysine	1.1908	10.9844	1.5219	0.0000	1.2843	0.8838	1.0573	2.7101	1.5620
Leucine	0.5901	10.0072	0.8330	0.3284	0.0000	1.1668	1.1668	1.6394	0.7743
Valine	0.6435	6.9927	0.8731	0.5313	0.0000	0.8651	0.2670	1.2736	0.6915
Tyrosine	0.0000	7.5107	0.9479	1.0493	0.0000	1.0867	0.0000	1.5513	0.9212
Threonine	0.0000	2.5979	0.0000	0.0000	0.0000	0.0000	0.0000	1.2576	2.3763
Glycine	0.9078	5.2225	0.8304	0.9025	0.4940	0.5527	0.5660	1.5459	0.6595
Isoleucine	0.3711	4.0744	0.4699	0.3444	0.0000	0.4940	0.4245	1.1801	0.6595
Methionine	0.3498	4.3067	0.7983	0.6675	0.0000	0.6168	0.3711	1.1481	0.5340
Glutamine	2.8516	2.8249	0.0000	0.0000	0.0000	0.0000	0.0000	0.0000	0.0000
Phenylalanine	0.0000	5.3854	0.6114	0.0000	0.0000	0.6408	0.0000	1.1908	0.8411
Ornithine	0.0000	2.7234	0.3177	0.0000	0.0000	0.0000	0.0000	0.0000	0.4486
Tyramine	0.9505	2.5232	0.9585	0.0000	0.0000	0.0000	0.0000	0.0000	0.0000
β-Alanine	0.0000	1.5219	0.0000	0.0000	0.0000	0.0000	0.0000	0.0000	0.0000
Putrescine	0.0000	0.0000	0.0000	0.0000	0.0000	0.0000	0.0000	0.6622	0.0000
Cadaverine	0.0000	1.2736	0.0000	0.9532	0.0000	0.0000	0.0000	0.0000	0.0000
Dimethylglycine	0.0000	0.3044	0.0561	0.0961	0.0000	0.0000	0.0000	0.0000	0.0000
Taurine	7.1075	10.0953	12.9548	30.6383	4.4562	0.6942	3.7567	25.9684	2.2802
Choline	0.4886	1.7061	0.0000	0.4859	0.3257	0.5073	0.3311	0.7983	0.3925
Tauro-conjugated bile acids	0.0000	0.0000	0.0000	0.0000	0.0000	0.0000	0.0000	0.0000	0.0000
Methylamine	0.4032	1.8476	0.2857	0.2563	0.2083	0.3952	0.3791	0.7449	0.5100
Trimethylamine	0.1175	1.1588	0.1789	0.2350	0.2056	0.1602	0.1068	0.4753	0.3231
Dimethylamine	0.1789	0.4779	0.1655	0.2750	0.0000	0.3845	0.1976	0.4753	0.0881
Glutamate	9.4411	65.3002	5.7031	9.0353	9.7455	4.4108	5.3026	11.1125	6.2024
2-Oxoglutarate	0.0000	1.3430	0.0000	0.0000	0.0000	0.0000	0.0000	0.4005	0.0000
Uracil	0.8731	9.3824	1.4471	2.5819	1.3537	1.4071	1.4258	1.9571	1.1748
Hypoxanthine	0.0000	8.6054	0.9345	0.0000	1.0733	0.8918	0.0000	0.8971	0.8624
Cytidine phosphate	0.0000	5.3907	0.0000	0.0000	0.0000	0.0000	0.0000	1.5833	0.0000
Guanosine phosphate	0.0000	1.5299	0.0000	0.0000	0.0000	0.0000	0.0000	1.3857	0.0000
Xanthine	0.0000	6.2558	0.0000	0.0000	0.0000	1.5326	0.0000	0.0000	0.0000
Uridine phosphate	0.0000	2.4564	0.0000	0.0000	0.0000	0.0000	0.0000	0.7449	0.0000
Adenosine phophate	0.0000	0.0000	0.0000	0.0000	0.0000	0.0000	0.0000	1.1214	0.0000
Creatinine	0.0000	1.0199	0.0000	0.2136	0.0000	0.0000	0.1388	0.0000	0.0000
Creatine	0.0614	0.0000	0.0000	0.0908	0.0000	0.0000	0.0000	0.0000	0.0000
4-Hydroxyphenylacetate	1.5353	1.9518	1.1081	1.6260	0.0000	0.9639	0.0000	3.2521	0.8063
Formate	2.2722	3.9783	2.1093	1.7916	0.0000	5.5376	1.7435	1.2522	0.9986
Lactate	0.6568	7.2384	0.4352	0.8784	0.0000	1.3751	0.4833	0.9105	0.0000
3-(3-Hydroxyphenyl)propanoate	0.0000	0.0000	0.0000	0.0000	0.0000	0.0000	0.0000	0.0000	0.0000
Acetate	91.551	336.956	63.2683	33.9571	30.6863	58.9403	20.7566	64.9291	27.3381
Propionate	28.061	72.5679	23.2477	13.8012	9.2168	17.2749	9.7775	20.0010	12.7226
Butyrate	19.087	186.144	13.0670	6.5175	9.3530	9.3210	5.1985	8.6428	4.8167
Succinate	4.6805	3.6499	3.6419	1.9384	0.4325	2.9637	1.3751	3.2307	0.9879
5-Aminovalerate	1.6741	1.9651	1.5166	1.6955	0.3338	0.0000	0.0000	4.0210	0.0000
Valerate	0.5046	6.9046	0.4940	0.8117	0.9078	0.3151	0.3151	0.9398	0.6995
Fumarate	1.2309	2.2802	0.8864	0.7102	0.0000	0.4406	0.0000	2.8329	0.3097
Isobutyrate	0.7449	3.6659	0.6942	0.9772	0.3364	0.7636	0.0000	1.1935	1.4525
Malate	0.0000	0.0000	0.0000	0.0000	0.0000	0.0000	0.0000	3.2868	0.0000
Isovalerate	0.4486	0.6809	0.0935	0.1629	0.2189	0.2296	0.0614	0.4245	0.2964
2-methylbutyrate	0.0000	1.6768	0.1495	0.2056	0.0721	0.0000	0.0000	0.4619	0.2590
Malonate	0.0000	1.7836	0.0000	0.0000	0.0000	0.0000	0.0000	0.2830	0.0000
Glucose	19.587	112.543	28.1338	15.0855	9.3290	27.7066	13.9988	40.2209	19.6272
Xylose	12.840	32.1094	24.6067	8.3090	6.3413	14.9493	10.5518	25.6320	12.9415

Sialate	2.1280	36.8594	10.1914	9.2783	4.4108	7.3772	2.0879	20.3721	15.7023
Glycerol	9.0566	15.0695	10.3836	5.5162	2.2615	13.7558	6.4934	13.0349	8.2690
Galactose	2.1947	10.9604	0.0000	0.7796	0.0000	6.4214	0.0000	6.3226	4.4509
Arabinose	3.0198	6.0102	3.3135	1.8476	0.0000	6.2825	1.8824	4.7366	2.6967
UDP-glucose	0.0000	20.5483	3.9783	0.0000	0.0000	0.0000	0.0000	0.0000	0.0000
Galacturonate	0.0000	10.8989	0.0000	0.0000	0.0000	0.0000	0.0000	2.4270	0.0000
Fucose	0.4646	3.0011	1.0013	0.0000	0.0000	1.2576	0.0000	2.5285	1.1561
Maltose	4.7766	0.0000	4.3628	0.0000	0.0000	4.1946	2.4190	0.0000	1.5913
1,3-Dihydroxyacetone	0.2270	0.7316	0.2884	0.3338	0.3097	0.3097	0.2483	0.4139	0.2857
Sucrose	0.0000	2.8943	0.0000	0.0000	0.0000	0.0000	0.0000	2.3763	0.7796
Nicotinate	0.0000	8.9605	0.0000	1.1534	0.0000	0.0000	0.0000	3.8742	0.0000

RAW metabolite concentrations (mM.kg-1)

Age	Young	Young	Young	Aged	Aged	Aged	Aged	Aged	Aged
Timepoint	Post FMT	Post FMT	Post FMT	Pre Abx	Pre Abx	Pre Abx	Pre Abx	Pre Abx	Pre Abx
Ethanol	70.0608	54.2144	42.3195	41.1981	43.1953	44.9201	40.9044	40.1354	66.2961
Methanol	10.2021	5.8553	3.1666	0.0000	4.7473	3.3215	4.8781	3.7674	6.8165
Aspartate	11.6439	13.5262	3.3962	7.8979	7.7483	12.2366	6.7498	16.1028	9.8683
Alanine	10.4210	5.9755	2.1253	13.5636	3.0812	6.5228	2.0586	3.5645	9.9057
Citrulline	8.1462	3.8555	1.9171	11.6332	3.9116	5.2546	2.8729	2.8088	7.8391
Lysine	0.0000	0.0000	0.0000	10.2234	4.5363	3.9810	2.2375	2.7448	8.8110
Leucine	6.6590	1.8770	1.1561	5.5563	2.4804	3.0572	0.7156	1.8557	5.2332
Valine	5.4869	1.9865	0.7289	12.2046	2.0693	2.3176	1.0440	1.3831	4.9368
Tyrosine	4.6298	2.3870	0.8277	6.9874	1.8717	2.0345	0.6515	2.2001	3.9356
Threonine	0.0000	0.6435	0.8170	39.6281	0.0000	0.0000	0.0000	0.6835	0.0000
Glycine	3.8795	2.6460	0.5020	0.0000	1.9144	2.4324	1.1641	0.9639	4.2640
Isoleucine	3.7113	1.8530	0.7209	4.3201	1.2362	1.4044	0.5340	1.2816	2.8195
Methionine	3.4123	1.3590	0.4245	2.2909	1.1828	1.2309	0.1255	1.1321	2.9744
Glutamine	4.5123	0.0000	0.0000	16.1588	1.3510	0.0000	3.3268	1.5887	0.0000
Phenylalanine	3.1800	1.7622	0.8784	2.9664	1.5112	1.8743	0.0000	0.9318	2.6593
Ornithine	1.9571	1.5620	0.3017	0.0000	0.5367	0.9398	0.0000	0.6221	0.0000
Tyramine	0.0000	0.0000	0.0000	0.0000	1.8370	0.0000	1.1534	0.6595	2.6006
β-Alanine	1.1214	0.0000	0.0000	0.0000	0.0000	0.0000	0.0000	0.0000	1.0253
Putrescine	0.5954	2.0666	0.4005	0.0000	0.6835	0.0000	0.0000	0.0000	1.8209
Cadaverine	0.0000	0.0000	0.2964	0.0000	0.0000	0.0000	0.0000	0.5393	0.0000
Dimethylglycine	0.1709	0.0000	0.0000	0.0000	0.0000	0.1228	0.0000	0.0481	0.2216
Taurine	70.4213	31.6956	10.6747	4.0077	13.7825	0.0000	0.0000	56.0753	26.1633
Choline	1.7302	1.2816	0.4005	29.1858	0.9852	0.0000	0.8197	0.6328	1.1962
Tauro-conjugated bile acids	0.0000	0.0000	0.0000	7.9272	0.0000	0.0000	0.0000	0.0000	0.0000
Methylamine	2.0746	0.9265	0.5847	0.0000	0.7209	1.6741	0.7796	0.5500	1.2095
Trimethylamine	3.4123	0.5420	0.1976	0.2270	0.2376	1.2229	0.4859	0.3284	2.3469
Dimethylamine	0.6355	0.7983	0.1549	0.0000	0.1522	0.0000	0.0000	0.3524	0.2723
Glutamate	20.6418	12.2873	4.2773	14.2017	10.9176	26.4383	14.1403	14.4928	32.4859
2-Oxoglutarate	0.7156	0.0000	0.0000	0.7503	0.0000	0.0000	0.5073	0.0000	1.1134
Uracil	4.4456	2.2642	0.9051	0.0000	1.3110	2.8088	0.9505	1.5353	11.1152
Hypoxanthine	2.9557	1.6394	0.5207	0.0000	1.7569	0.9345	1.3190	1.2709	5.8046
Cytidine phosphate	3.8715	2.0826	0.0000	0.0000	2.7554	0.0000	0.0000	1.7462	4.8567
Guanosine phosphate	3.1693	2.2295	1.5112	0.0000	2.5712	0.0000	0.0000	2.1040	0.0000
Xanthine	0.0000	1.8049	0.0000	0.0000	0.0000	0.5447	0.0000	0.0000	2.8756
Uridine phosphate	2.2588	0.9265	0.0000	0.0000	1.0039	0.0000	0.0000	0.0000	1.6127
Adenosine phophate	1.7756	0.8651	0.4806	0.0000	0.0000	0.0000	0.0000	1.4632	0.6141
Creatinine	1.0920	0.0000	0.0000	6.5308	0.0000	0.0000	0.0000	0.0000	0.0000
Creatine	2.4724	0.4486	0.0000	3.2547	0.0000	0.0000	0.1682	0.0000	0.0000
4-Hydroxyphenylacetate	3.3642	3.2547	1.7489	0.0000	0.9185	0.7983	1.0894	2.6406	6.5068
Formate	2.0586	1.8503	1.0867	1.5913	1.7195	1.6607	1.3911	1.9731	0.0000
Lactate	3.0812	1.0173	0.9932	0.0000	4.1812	1.7355	0.8224	0.6782	2.6166
3-(3-Hydroxyphenyl)propanoate	0.5153	0.9559	0.6008	0.0000	1.3430	0.0000	0.0000	0.0000	0.0000
Acetate	197.6628	104.9043	54.5721	0.0000	97.7140	103.5212	111.1094	59.0791	302.5511
Propionate	67.2146	41.7054	17.7902	0.0000	27.1058	26.9109	29.9975	21.0663	100.9580
Butyrate	37.5322	26.4837	10.1914	0.0000	21.3039	16.2229	32.5446	8.5413	67.2920
Succinate	7.0274	16.4552	4.3521	0.4993	26.9056	0.9852	1.1134	3.2975	7.0088
5-Aminovalerate	10.0499	11.4970	1.7008	0.0000	7.3105	1.3217	1.0947	1.0680	2.3069

Valerate	2.5685	0.8411	0.8811	0.0000	2.0132	1.7969	1.4285	2.0559	8.8297
Fumarate	4.0771	3.6179	0.6034	0.0000	0.5474	1.5246	0.6061	2.7661	1.0199
Isobutyrate	1.2122	2.2081	1.6207	0.0000	0.8517	0.0000	0.0000	0.7289	5.0490
Malate	2.7608	4.9822	1.7969	0.0000	0.0000	0.0000	0.0000	0.0000	0.0000
Isovalerate	0.8784	0.6969	0.2723	0.0000	0.3444	0.4886	0.1522	0.4779	3.5965
2-methylbutyrate	0.7129	0.5020	0.1228	0.0000	0.3498	0.2189	0.2643	0.2029	1.9384
Malonate	0.0000	0.5340	0.3017	0.0000	0.3845	0.8437	0.4459	0.4779	1.1081
Glucose	112.8956	66.4136	22.3853	9.7589	53.6270	29.3727	29.2071	26.9083	83.0263
Xylose	53.4134	42.8321	18.8475	0.0000	36.8754	31.1269	22.0729	15.9719	58.7000
Sialate	52.1184	24.8390	13.1204	7.2357	15.8999	32.2269	6.1437	26.1820	68.0396
Glycerol	29.7491	21.9527	8.9498	0.0000	17.5926	13.0323	19.7046	9.3931	26.3930
Galactose	12.8400	6.0903	4.1732	7.8738	4.5684	3.6205	2.0185	3.2414	8.2877
Arabinose	12.5437	9.0540	3.7220	3.8501	7.6122	3.8688	5.5242	2.3977	10.0819
UDP-glucose	9.3210	3.9650	0.0000	0.0000	1.6474	6.1357	3.2547	6.8005	18.3669
Galacturonate	7.8525	0.0000	0.0000	0.0000	1.5166	0.0000	0.0000	5.6150	7.3131
Fucose	6.4881	3.1426	1.7115	0.0000	1.2175	2.1574	0.9158	1.0173	4.3815
Maltose	0.0000	4.6965	2.9664	0.0000	0.0000	1.8290	5.4735	0.0000	0.0000
1,3-Dihydroxyacetone	0.6061	0.5313	0.3551	0.0000	0.2910	0.3952	0.3791	0.3311	0.6542
Sucrose	1.5086	0.0000	0.0000	0.0000	0.8651	0.0000	0.0000	0.0000	0.0000
Nicotinate	2.4991	2.5552	0.0000	0.0000	0.0000	2.6647	1.5459	1.2469	5.0009

RAW metabolite concentrations (mM.kg-1)

Age	Aged	Aged	Aged	Aged	Aged	Aged	Aged	Aged	Aged
Timepoint	Pre Abx	Pre Abx	Post FMT	Post FMT	Post FMT	Post FMT	Post FMT	Post FMT	Post FMT
Ethanol	45.2485	45.0643	42.3248	70.5628	49.6246	42.3542	48.8503	60.1711	88.7054
Methanol	4.1732	5.0249	4.9796	10.3035	2.5205	5.3747	6.7391	5.9408	17.4671
Aspartate	7.8445	12.5837	6.4347	18.7674	4.5764	8.9792	8.8003	7.2277	29.3059
Alanine	1.3751	6.2398	4.7553	24.4145	5.2439	6.9927	4.4108	3.3642	18.2067
Citrulline	0.0000	4.8621	5.3453	12.0364	3.5191	5.9701	4.3761	4.1919	10.0499
Lysine	1.7141	7.8498	4.1225	12.0844	5.8660	7.3345	3.5297	3.2147	11.1713
Leucine	0.5500	4.7366	2.7047	8.7736	2.6513	4.4696	1.2976	1.8290	11.0164
Valine	0.4592	3.1186	2.4457	8.3731	2.3176	3.8368	0.9879	1.5299	9.3797
Tyrosine	0.4406	4.0344	2.5739	8.1488	2.9904	3.1319	2.1146	1.3430	8.4425
Threonine	0.0000	0.0000	0.0000	2.6540	2.3603	0.0000	1.3724	0.0000	11.8655
Glycine	0.8597	2.6700	1.3297	6.3653	1.9144	2.7020	2.0879	1.4712	10.7921
Isoleucine	0.5794	2.2562	2.0906	5.3987	0.8063	2.3736	0.8544	0.9238	5.2065
Methionine	0.4112	3.2627	1.8049	6.3893	1.4632	2.0773	1.4044	1.1695	5.2332
Glutamine	3.4443	1.4231	1.7275	4.1198	0.0000	1.3857	0.0000	0.0000	0.0000
Phenylalanine	0.0000	2.6673	1.9144	5.4148	1.3804	2.3549	0.8971	0.6248	3.6606
Ornithine	0.0000	1.2255	1.1134	2.1040	1.3697	1.4552	1.1775	0.5313	4.6058
Tyramine	0.0000	1.0974	0.0000	1.3697	0.0000	0.0000	1.5860	0.7182	3.1079
β-Alanine	0.0000	0.9959	0.0000	2.6433	4.0985	1.3003	0.0000	0.0000	1.8530
Putrescine	0.0000	0.0000	0.8224	1.6127	0.5821	0.0000	1.0787	0.4459	1.5780
Cadaverine	0.0000	0.9372	0.0000	0.0000	0.0000	0.0000	0.0000	0.0000	1.2522
Dimethylglycine	0.0000	0.1175	0.0000	0.0000	0.0000	0.0000	0.0000	0.0000	0.2376
Taurine	25.5119	6.7551	0.0000	15.2350	0.0000	0.0000	42.7787	49.1333	74.7680
Choline	0.3311	0.7236	0.8063	2.1520	0.6168	1.0360	1.0840	0.7423	0.0000
Tauro-conjugated bile acids	0.0000	0.0000	1.4018	2.2321	0.8170	0.9398	0.0000	0.0000	4.2720
Methylamine	0.4005	0.9131	0.5714	1.5887	0.5393	0.8704	0.3845	0.4539	1.2736
Trimethylamine	0.2537	1.0440	0.3284	1.1454	0.4619	1.0093	0.3605	0.3444	1.1027
Dimethylamine	0.0000	0.3231	0.0000	0.0000	0.0000	0.1362	0.1736	0.1442	0.7957
Glutamate	9.8363	35.2600	9.4358	46.3218	27.4529	43.3902	14.0095	8.2343	47.8918
2-Oxoglutarate	0.7743	0.0000	0.0000	0.0000	0.0000	0.0000	0.0000	0.0000	0.0000
Uracil	0.0000	4.7366	2.0960	10.8002	8.0234	3.5965	3.4470	1.7141	8.9819
Hypoxanthine	0.0000	4.6698	0.7449	3.7781	5.1958	2.9050	1.2843	1.7061	4.6965
Cytidine phosphate	0.0000	5.3347	0.0000	10.1727	0.0000	0.0000	0.0000	0.0000	9.8657
Guanosine phosphate	0.0000	0.0000	1.5593	7.5428	0.0000	0.0000	1.1748	1.5780	5.2946
Xanthine	0.0000	4.9609	2.0559	4.0210	0.0000	2.5338	1.1161	0.0000	3.5057
Uridine phosphate	0.0000	2.0372	0.0000	0.0000	0.0000	0.0000	0.0000	0.0000	5.7005
Adenosine phophate	0.0000	1.3270	0.0000	1.7222	0.0000	0.0000	0.0000	0.0000	3.3535
Creatinine	0.0000	0.6568	0.0000	0.0000	0.0000	0.0000	0.0000	0.0000	0.0000
Creatine	0.0000	0.0000	0.0000	0.0000	0.0000	0.0000	0.0000	0.0000	0.0000
4-Hydroxyphenylacetate	0.9505	3.4310	1.1801	4.3788	0.0000	0.0000	2.6940	3.8101	17.1227

Formate	1.6180	1.1348	1.5326	3.3589	0.0000	1.6821	0.9906	1.5246	3.6899
Lactate	0.0000	2.3229	1.4979	2.2989	0.0000	2.3923	0.0000	0.6542	0.0000
3-(3-Hydroxyphenyl)propanoate	0.0000	0.3685	0.6782	1.6234	0.0000	0.0000	0.0000	0.0000	0.0000
Acetate	87.6187	216.4569	115.9821	451.2220	197.9138	295.3581	150.3664	133.1689	483.2620
Propionate	23.4186	55.6214	22.5989	115.9234	18.2842	28.8387	39.3291	44.9414	159.6927
Butyrate	15.3018	38.8912	11.7934	76.0817	50.3776	44.5570	16.6154	24.6121	53.3867
Succinate	2.1600	4.5604	5.1478	13.3313	1.1988	1.0867	6.7284	9.5960	12.9228
5-Aminovalerate	0.5741	2.0399	0.8624	5.4441	0.9532	2.0105	0.9906	6.6376	24.8016
Valerate	1.3110	5.1131	1.8156	7.5347	0.0000	3.6953	1.7675	2.6513	10.5892
Fumarate	0.6648	2.4858	0.8544	5.8019	0.2590	0.7316	2.6219	1.9277	14.6957
Isobutyrate	0.8678	2.8916	1.2149	5.7779	0.0000	1.2976	2.2428	2.0746	7.4920
Malate	0.0000	0.0000	0.0000	7.7216	0.0000	0.0000	2.7581	0.0000	11.8495
Isovalerate	1.7035	1.1615	0.2884	1.9758	0.0000	0.4406	0.7823	0.6675	3.8395
2-methylbutyrate	0.2350	1.2068	0.0801	1.5620	0.4299	0.2964	0.5981	0.6461	2.4110
Malonate	0.3391	1.5780	0.0000	2.2481	0.8411	0.8678	0.0000	0.0000	0.0000
Glucose	21.7365	49.3897	52.9648	114.8127	25.4665	60.6784	43.2727	43.7106	103.3958
Xylose	16.4953	24.8203	19.1092	88.4064	14.5835	22.8819	33.7755	20.6364	81.8542
Sialate	19.0905	41.9190	33.2896	79.8784	0.0000	0.0000	18.6820	26.7053	120.2435
Glycerol	10.0606	14.3192	16.0948	56.4251	6.5629	13.7959	18.9623	10.9657	39.4359
Galactose	2.7127	10.6640	4.5443	11.4944	3.0732	6.9794	4.1065	4.1465	29.1991
Arabinose	4.4509	3.7006	7.7991	17.4111	3.6766	8.1355	6.0075	2.6700	10.4611
UDP-glucose	0.0000	25.0019	4.8087	17.5312	14.2818	11.8575	0.0000	0.0000	0.0000
Galacturonate	0.0000	6.0956	3.7834	26.2728	3.7433	6.5549	0.0000	2.2642	26.4437
Fucose	1.1081	2.9610	1.6314	4.8113	1.3564	1.3217	1.7595	1.6928	11.3929
Maltose	0.0000	0.0000	0.0000	0.0000	2.5685	0.0000	0.0000	0.0000	0.0000
1,3-Dihydroxyacetone	0.3151	0.4993	0.2350	0.0000	0.1869	0.2670	0.4859	0.5340	1.7355
Sucrose	0.0000	0.0000	0.0000	0.0000	0.0000	0.0000	0.0000	0.0000	0.0000
Nicotinate	0.0000	3.9783	1.1641	8.2022	5.1184	3.9703	1.2469	1.8557	6.6002
