# Supplementary material for: Fecal microbiota transfer between young and aged mice reverses hallmarks of the aging gut, eye, and brain
Source: Microbiome. 2022 Apr 29;10:68. doi: 10.1186/s40168-022-01243-w (PMC9063061; doi:10.1186/s40168-022-01243-w)
Supplement: Supplementary file 5 — Additional file 4: Table S4. Full list of differentially abundant metabolic pathways Pre– vs. Post–FMT. Relates to Fig. 7. Provided as a separate Excel file. [file 40168_2022_1243_MOESM5_ESM.pdf]

Table S4. Significantly differentially-abundant pathways

	Comparison 1 aged mice + young FMT, post vs pre FMT								
Pathway Name (short)	contrast	estimate	SE	df	lower.CL	upper.CL	t.ratio	p.value	padjust
NONMEVIPP-PWY	1	-9.45667	0.69562	27.47488	-10.88281	-8.03053	-13.59457	0.00000	0.00000
PWY-6270	1	-8.73121	0.60386	27.47490	-9.96923	-7.49319	-14.45897	0.00000	0.00000
PWY-5121	1	-8.72411	0.48498	27.46902	-9.71842	-7.72981	-17.98856	0.00000	0.00000
PWY-5971	1	-8.62390	1.55347	27.40959	-11.80913	-5.43867	-5.55138	0.00001	0.00020
PWY-7560	1	-8.58546	0.68401	27.47383	-9.98781	-7.18311	-12.55157	0.00000	0.00000
PWY-6113	1	-8.49061	1.48837	27.41375	-11.54233	-5.43888	-5.70463	0.00000	0.00014
PWY-7392	1	-8.48279	0.37928	27.48869	-9.26037	-7.70522	-22.36543	0.00000	0.00000
PWY-6284	1	-8.06959	1.35175	27.41938	-10.84116	-5.29801	-5.96973	0.00000	0.00008
PWY-5367	1	-7.45408	1.22771	27.41453	-9.97135	-4.93681	-6.07153	0.00000	0.00008
PWY-7196	1	-5.26104	1.15455	27.42563	-7.62827	-2.89382	-4.55679	0.00010	0.00145
FOLSYN-PWY	1	-3.80191	1.42452	27.47285	-6.72244	-0.88138	-2.66890	0.01262	0.03544
PWY-6612	1	-3.73729	1.36452	27.47948	-6.53477	-0.93981	-2.73891	0.01069	0.03163
P122-PWY	1	-3.39368	1.15056	27.19633	-5.75364	-1.03373	-2.94959	0.00647	0.02327
P124-PWY	1	-3.36123	1.35708	27.49036	-6.14339	-0.57906	-2.47682	0.01969	0.04678
PWY-7388	1	-2.51628	0.83140	27.19650	-4.22161	-0.81096	-3.02655	0.00536	0.02113
FASYN-INITIAL-PWY	1	-2.51111	0.83445	27.19303	-4.22269	-0.79954	-3.00932	0.00559	0.02144
PWY0-862	1	-2.46119	0.84988	27.18197	-4.20447	-0.71792	-2.89591	0.00738	0.02485
PWY-6282	1	-2.45558	0.84542	27.18550	-4.18968	-0.72147	-2.90456	0.00722	0.02485
PWY-7664	1	-2.41917	0.85699	27.18496	-4.17700	-0.66133	-2.82288	0.00880	0.02844
PWY-5989	1	-2.41672	0.67601	27.12011	-3.80350	-1.02995	-3.57497	0.00134	0.00931
PWY0-1061	1	-2.40407	1.53275	27.45850	-5.54655	0.73842	-1.56847	0.12823	0.20797
PWYG-321	1	-2.40123	0.86151	27.18591	-4.16833	-0.63413	-2.78724	0.00958	0.03009
PWY-6859	1	-2.32230	1.18092	27.49036	-4.74332	0.09873	-1.96651	0.05941	0.11590
PWY-6519	1	-2.30522	0.85244	27.17997	-4.05374	-0.55670	-2.70427	0.01167	0.03344
FASYN-ELONG-PWY	1	-2.28994	0.86084	27.18882	-4.05565	-0.52422	-2.66013	0.01294	0.03577
PWY0-781	1	-2.26085	1.30559	27.48777	-4.93749	0.41578	-1.73167	0.09454	0.16759
BIOTIN-BIOSYNTHESIS-PWY	1	-2.22211	0.86726	27.17445	-4.00104	-0.44318	-2.56223	0.01626	0.04108
P4-PWY	1	-2.20794	1.31795	27.48676	-4.90991	0.49403	-1.67528	0.10522	0.18282
PWY0-1241	1	-2.10181	0.69986	27.40797	-3.53680	-0.66682	-3.00320	0.00565	0.02151
PYRIDOXSYN-PWY	1	-1.67129	1.00009	27.47959	-3.72163	0.37905	-1.67114	0.10605	0.18343
PWY0-845	1	-1.64855	1.21789	27.48745	-4.14538	0.84827	-1.35362	0.18689	0.27635
PWY-6147	1	-1.51282	0.30324	27.49036	-2.13450	-0.89114	-4.98885	0.00003	0.00057
PWY-7539	1	-1.50752	0.30397	27.49036	-2.13069	-0.88436	-4.95952	0.00003	0.00060
FUCCAT-PWY	1	-1.40751	0.30793	27.46722	-2.03882	-0.77620	-4.57093	0.00009	0.00144
PWY-5941	1	-1.40152	0.30354	27.46972	-2.02384	-0.77920	-4.61720	0.00008	0.00131
MET-SAM-PWY	1	-1.36577	0.56832	27.44624	-2.53098	-0.20056	-2.40317	0.02327	0.05369
HOMOSER-METSYN-PWY	1	-1.26068	0.54287	27.45934	-2.37368	-0.14767	-2.32224	0.02787	0.06333
METSYN-PWY	1	-1.18903	0.57778	27.45704	-2.37361	-0.00445	-2.05793	0.04920	0.09876
PWY-5347	1	-1.15838	0.58263	27.45042	-2.35292	0.03615	-1.98820	0.05685	0.11208
PWY-7282	1	-1.02103	1.30835	27.48649	-3.70331	1.66126	-0.78039	0.44183	0.53637
ARGININE-SYN4-PWY	1	-0.98989	0.29696	27.49036	-1.59869	-0.38109	-3.33342	0.00246	0.01304
PWY-7117	1	-0.78426	0.84059	27.49036	-2.50758	0.93905	-0.93299	0.35895	0.46091

PWY-241	1	-0.77612	0.83862	27.49036	-2.49539	0.94315	-0.92547	0.36277	0.46368
PWY-5913	1	-0.68417	0.26836	27.49036	-1.23435	-0.13399	-2.54941	0.01667	0.04175
PWY0-1297	1	-0.62833	0.82531	27.49036	-2.32030	1.06365	-0.76133	0.45294	0.54392
CITRULBIO-PWY	1	-0.60570	0.30365	27.46590	-1.22825	0.01685	-1.99472	0.05608	0.11097
P161-PWY	1	-0.58343	0.64400	27.49036	-1.90370	0.73684	-0.90595	0.37283	0.47280
PWY-7237	1	-0.50550	0.18596	27.32849	-0.88685	-0.12415	-2.71830	0.01126	0.03260
COBALSYN-PWY	1	-0.49349	0.68757	27.49036	-1.90309	0.91612	-0.71772	0.47898	0.56905
PWY-6608	1	-0.47557	0.24447	27.49036	-0.97676	0.02562	-1.94532	0.06203	0.11852
PENTOSE-P-PWY	1	-0.46779	0.19449	27.46937	-0.86654	-0.06905	-2.40521	0.02316	0.05365
POLYISOPRENSYN-PWY	1	-0.40683	0.73420	27.19696	-1.91278	1.09912	-0.55411	0.58403	0.65468
PWY4LZ-257	1	-0.39479	0.78656	27.49036	-2.00734	1.21776	-0.50191	0.61972	0.68096
PWY-1269	1	-0.39076	0.27383	27.49036	-0.95215	0.17062	-1.42702	0.16483	0.25178
FERMENTATION-PWY	1	-0.37981	0.21977	27.46757	-0.83038	0.07076	-1.72820	0.09518	0.16800
GLYCOLYSIS-E-D	1	-0.32930	0.23793	27.49036	-0.81708	0.15849	-1.38401	0.17749	0.26671
P441-PWY	1	-0.32313	0.88670	27.49036	-2.14096	1.49471	-0.36442	0.71834	0.76366
GLCMANNANAUT-PWY	1	-0.19046	0.23033	27.48226	-0.66267	0.28174	-0.82693	0.41540	0.51286
ANAEROFRUCAT-PWY	1	-0.14560	0.21609	27.44015	-0.58865	0.29744	-0.67380	0.50607	0.59315
PHOSLIPSYN-PWY	1	-0.13676	0.27804	27.49036	-0.70678	0.43326	-0.49187	0.62672	0.68729
PWY4FS-7	1	-0.11656	0.24209	27.49036	-0.61288	0.37975	-0.48149	0.63398	0.69252
PWY4FS-8	1	-0.11656	0.24209	27.49036	-0.61288	0.37975	-0.48149	0.63398	0.69252
SER-GLYSYN-PWY	1	-0.09010	0.17534	27.42656	-0.44960	0.26940	-0.51389	0.61144	0.67557
PWY-6901	1	-0.04839	0.18994	27.46295	-0.43782	0.34103	-0.25477	0.80080	0.83223
PWY0-162	1	0.03876	0.23015	27.31545	-0.43322	0.51074	0.16841	0.86750	0.88664
PWY-6703	1	0.22127	0.24448	27.48070	-0.27996	0.72250	0.90505	0.37330	0.47280
PWY-7383	1	0.30324	0.26827	27.36664	-0.24686	0.85335	1.13036	0.26813	0.35837
PWY-5484	1	0.36173	0.18634	27.49036	-0.02029	0.74375	1.94124	0.06254	0.11869
GLYCOLYSIS	1	0.38561	0.18937	27.49036	-0.00261	0.77383	2.03632	0.05146	0.10254
HEXITOLDEGSUPER-PWY	1	0.46504	0.17765	27.49036	0.10084	0.82924	2.61774	0.01422	0.03717
PWY-6353	1	0.48152	0.18670	27.28111	0.09862	0.86441	2.57907	0.01561	0.03963
PWY66-400	1	0.50331	0.20178	27.49036	0.08964	0.91698	2.49436	0.01892	0.04613
RIBOSYN2-PWY	1	0.51514	0.20961	27.36986	0.08533	0.94495	2.45762	0.02060	0.04852
PWY-6126	1	0.54306	0.19396	27.47045	0.14541	0.94071	2.79988	0.00925	0.02938
COMPLETE-ARO-PWY	1	0.54431	0.20561	27.43472	0.12274	0.96589	2.64726	0.01329	0.03621
PWY-7221	1	0.55973	0.19728	27.41583	0.15524	0.96422	2.83729	0.00846	0.02767
PANTO-PWY	1	0.57496	0.22211	27.39227	0.11954	1.03038	2.58868	0.01524	0.03905
GLYCOGENSYNTH-PWY	1	0.57769	0.19610	27.49036	0.17567	0.97972	2.94594	0.00649	0.02327
PWY-5695	1	0.58734	0.23122	27.43712	0.11327	1.06140	2.54020	0.01704	0.04192
PWY-7229	1	0.58744	0.18970	27.46822	0.19851	0.97636	3.09665	0.00447	0.01908
PANTOSYN-PWY	1	0.60596	0.20978	27.37738	0.17579	1.03612	2.88849	0.00748	0.02505
PWY-2942	1	0.63915	0.20531	27.40825	0.21818	1.06013	3.11304	0.00430	0.01908
PWY-6385	1	0.64438	0.19438	27.41454	0.24583	1.04294	3.31504	0.00259	0.01356
PWY-6386	1	0.64902	0.19370	27.41079	0.25185	1.04619	3.35061	0.00236	0.01300
PWY-4242	1	0.64964	0.19831	27.34386	0.24298	1.05630	3.27591	0.00286	0.01487
OANTIGEN-PWY	1	0.65335	0.19081	27.42842	0.26211	1.04458	3.42399	0.00196	0.01140
PYRIDNUCSYN-PWY	1	0.65928	0.22114	27.49036	0.20591	1.11265	2.98122	0.00595	0.02220
1CMET2-PWY	1	0.66102	0.20510	27.34296	0.24043	1.08161	3.22289	0.00327	0.01582

P42-PWY	1	0.67353	0.25676	27.37952	0.14705	1.20001	2.62323	0.01407	0.03717
PWY-5097	1	0.67476	0.20208	27.42100	0.26042	1.08910	3.33905	0.00243	0.01304
ARO-PWY	1	0.68132	0.20148	27.40400	0.26819	1.09444	3.38152	0.00218	0.01239
COA-PWY-1	1	0.69149	0.20060	27.38610	0.28016	1.10282	3.44710	0.00185	0.01116
PWY-6163	1	0.69378	0.20273	27.37660	0.27808	1.10949	3.42218	0.00197	0.01140
PWY-5667	1	0.70487	0.20144	27.46961	0.29188	1.11785	3.49918	0.00161	0.01017
PWY0-1319	1	0.70487	0.20144	27.46961	0.29188	1.11785	3.49918	0.00161	0.01017
PEPTIDOGLYCANSYN-PWY	1	0.73379	0.19328	27.38024	0.33746	1.13012	3.79642	0.00074	0.00591
PWY-6387	1	0.75030	0.19211	27.38481	0.35638	1.14423	3.90551	0.00056	0.00492
PWY-2941	1	0.76043	0.27758	27.39093	0.19126	1.32961	2.73947	0.01070	0.03163
PWY-7219	1	0.76491	0.20266	27.44350	0.34939	1.18043	3.77426	0.00079	0.00607
PWY-5686	1	0.77482	0.20103	27.36879	0.36261	1.18703	3.85436	0.00064	0.00522
PWY-6122	1	0.78821	0.19562	27.38639	0.38710	1.18933	4.02931	0.00040	0.00429
PWY-6277	1	0.78821	0.19562	27.38639	0.38710	1.18933	4.02931	0.00040	0.00429
PWY-6121	1	0.78831	0.19631	27.38979	0.38578	1.19085	4.01556	0.00042	0.00430
TEICHOICACID-PWY	1	0.81477	0.21639	27.28157	0.37100	1.25854	3.76535	0.00081	0.00617
PWY-7199	1	0.82945	0.19338	27.41156	0.43295	1.22596	4.28929	0.00020	0.00264
PWY-5676	1	0.84318	0.25245	27.47053	0.32561	1.36074	3.34000	0.00242	0.01304
ARGSYNBSUB-PWY	1	0.84460	0.24089	27.48806	0.35075	1.33844	3.50623	0.00158	0.01017
PWY-6151	1	0.87582	0.21918	27.32815	0.42635	1.32530	3.99583	0.00044	0.00437
ARGSYN-PWY	1	0.87868	0.23237	27.46327	0.40226	1.35509	3.78130	0.00077	0.00604
PWY-6609	1	0.88922	0.21022	27.47975	0.45825	1.32020	4.23005	0.00023	0.00295
PWY-4981	1	0.90363	0.19006	27.35681	0.51390	1.29337	4.75443	0.00006	0.00096
PWY-1042	1	0.90639	0.22810	27.44854	0.43872	1.37406	3.97361	0.00046	0.00444
PWY66-399	1	0.92055	0.23106	27.44439	0.44681	1.39428	3.98401	0.00045	0.00440
GLUTORN-PWY	1	0.93337	0.24209	27.44187	0.43702	1.42972	3.85551	0.00063	0.00522
SALVADEHYPOX-PWY	1	0.94136	0.25153	27.46605	0.42567	1.45705	3.74251	0.00085	0.00641
PWY66-422	1	0.96468	0.22053	27.49036	0.51257	1.41679	4.37440	0.00016	0.00220
PWY-7234	1	0.97782	0.20954	27.30157	0.54810	1.40754	4.66646	0.00007	0.00119
PWY-6317	1	1.00088	0.22377	27.49036	0.54212	1.45964	4.47276	0.00012	0.00173
PWY0-1296	1	1.13124	0.24871	27.49036	0.62135	1.64112	4.54844	0.00010	0.00145
PWY-5104	1	1.25249	0.24776	27.49036	0.74454	1.76043	5.05520	0.00003	0.00052
PWY-5177	1	1.27896	0.19731	27.25423	0.87430	1.68362	6.48212	0.00000	0.00004
GALACT-GLUCUROCAT-PWY	1	1.31469	0.21011	27.34243	0.88382	1.74555	6.25701	0.00000	0.00005
PWY-7242	1	1.35640	0.22619	27.32466	0.89255	1.82025	5.99661	0.00000	0.00008
PWY-5505	1	1.35893	0.66920	27.48538	-0.01302	2.73089	2.03068	0.05206	0.10338
PWY-6527	1	1.37634	0.25332	27.48370	0.85700	1.89569	5.43322	0.00001	0.00025
PWY-6507	1	1.43688	0.23900	27.31182	0.94675	1.92701	6.01199	0.00000	0.00008
GALACTUROCAT-PWY	1	1.46877	0.22076	27.39853	1.01612	1.92143	6.65321	0.00000	0.00002
PWY-6305	1	1.49467	0.28802	27.49036	0.90419	2.08515	5.18943	0.00002	0.00042
PWY-7315	1	1.54683	0.29993	27.48963	0.93193	2.16172	5.15726	0.00002	0.00042
PPGPPMET-PWY	1	1.92538	0.57392	27.45618	0.74870	3.10205	3.35478	0.00233	0.01298
METH-ACETATE-PWY	1	2.62523	0.66427	27.49036	1.26341	3.98706	3.95207	0.00049	0.00462
PWY-6590	1	2.89695	1.05886	27.48666	0.72616	5.06775	2.73593	0.01077	0.03168
CENTFERM-PWY	1	2.90040	1.03569	27.48664	0.77709	5.02371	2.80044	0.00923	0.02938

PWY-7090	1	3.34372	1.31434	27.49036	0.64917	6.03828	2.54403	0.01688	0.04189
PWY-5088	1	3.37242	1.26876	27.29819	0.77046	5.97438	2.65804	0.01298	0.03577
PWY-6834	1	3.42734	1.24192	27.34621	0.88063	5.97404	2.75970	0.01020	0.03116
PWY-5005	1	3.85031	0.96110	27.46101	1.87984	5.82078	4.00615	0.00042	0.00430
PWY-4321	1	4.20673	1.52665	27.45052	1.07670	7.33676	2.75552	0.01028	0.03125
ARGORNPROST-PWY	1	5.35262	1.72391	27.49036	1.81840	8.88684	3.10493	0.00438	0.01908
POLYAMINSYN3-PWY	1	7.67108	1.40652	27.47113	4.78746	10.55471	5.45394	0.00001	0.00025
P185-PWY	1	8.33453	1.32732	27.49036	5.61336	11.05570	6.27920	0.00000	0.00005
P562-PWY	1	8.81027	1.30813	27.42029	6.12814	11.49240	6.73503	0.00000	0.00002
ALLANTOINDEG-PWY	1	9.09310	1.06657	27.47829	6.90645	11.27974	8.52553	0.00000	0.00000

Comparison 2 young mice + aged FMT, post vs pre FMT

	contrast	estimate	SE	df	lower.CL	upper.CL	t.ratio	p.value	padjust
PWY-7211	2	-6.15590	1.71551	27.46824	-9.67302	-2.63878	-3.58839	0.00128	0.00899
PWY-7210	2	-5.87327	1.76088	27.49036	-9.48328	-2.26326	-3.33542	0.00245	0.01304
P562-PWY	2	-3.98897	1.39845	27.42029	-6.85628	-1.12165	-2.85243	0.00816	0.02697
PWY-2941	2	-1.55555	0.29675	27.39093	-2.16403	-0.94708	-5.24199	0.00002	0.00040
COBALSYN-PWY	2	-1.37579	0.73504	27.49036	-2.88272	0.13114	-1.87171	0.07192	0.13198
GLYCOLYSIS-E-D	2	-1.23357	0.25436	27.49036	-1.75503	-0.71211	-4.84979	0.00004	0.00076
PWY-7539	2	-1.14235	0.32495	27.49036	-1.80855	-0.47616	-3.51544	0.00154	0.01017
PWY0-1586	2	-1.13346	0.22634	27.49036	-1.59748	-0.66943	-5.00779	0.00003	0.00057
PWY-6147	2	-1.12971	0.32418	27.49036	-1.79431	-0.46510	-3.48484	0.00167	0.01031
PWY4FS-7	2	-1.08717	0.25880	27.49036	-1.61775	-0.55659	-4.20075	0.00025	0.00305
PWY4FS-8	2	-1.08717	0.25880	27.49036	-1.61775	-0.55659	-4.20075	0.00025	0.00305
UDPNAGSYN-PWY	2	-1.08498	0.26331	27.48402	-1.62480	-0.54516	-4.12054	0.00031	0.00362
PWY-7237	2	-1.01136	0.19880	27.32849	-1.41904	-0.60368	-5.08723	0.00002	0.00050
THISYNARA-PWY	2	-1.00648	0.24322	27.49036	-1.50511	-0.50785	-4.13815	0.00030	0.00353
ARGININE-SYN4-PWY	2	-1.00085	0.31746	27.49036	-1.65169	-0.35002	-3.15266	0.00389	0.01772
PWY-6901	2	-0.99644	0.20306	27.46295	-1.41275	-0.58012	-4.90712	0.00004	0.00067
PWY-7228	2	-0.95029	0.27116	27.47446	-1.50622	-0.39435	-3.50446	0.00159	0.01017
NAGLIPASYN-PWY	2	-0.94791	0.32583	27.49036	-1.61590	-0.27991	-2.90917	0.00710	0.02482
PWY-6125	2	-0.94314	0.25553	27.47489	-1.46703	-0.41926	-3.69091	0.00098	0.00724
PWY-7208	2	-0.92463	0.26329	27.47473	-1.46442	-0.38484	-3.51181	0.00156	0.01017
HISTSYN-PWY	2	-0.87104	0.22367	27.44409	-1.32964	-0.41245	-3.89424	0.00057	0.00497
PWY-7220	2	-0.86863	0.22236	27.43087	-1.32454	-0.41272	-3.90641	0.00055	0.00492
PWY-7222	2	-0.86863	0.22236	27.43087	-1.32454	-0.41272	-3.90641	0.00055	0.00492
THRESYN-PWY	2	-0.86175	0.22346	27.49036	-1.31988	-0.40362	-3.85632	0.00063	0.00522
PWY-5154	2	-0.82966	0.29499	27.49036	-1.43443	-0.22488	-2.81245	0.00897	0.02882
OANTIGEN-PWY	2	-0.82745	0.20399	27.42842	-1.24569	-0.40920	-4.05632	0.00037	0.00414
PWY-6700	2	-0.80902	0.25321	27.48100	-1.32813	-0.28991	-3.19512	0.00350	0.01647
HEXITOLDEGSUPER-PWY	2	-0.80425	0.18992	27.49036	-1.19360	-0.41490	-4.23477	0.00023	0.00295
PWY-3001	2	-0.79983	0.21791	27.49036	-1.24657	-0.35309	-3.67048	0.00103	0.00753
HISDEG-PWY	2	-0.79623	0.29017	27.47510	-1.39114	-0.20133	-2.74399	0.01057	0.03158
GLUCONEO-PWY	2	-0.71843	0.20974	27.49036	-1.14842	-0.28844	-3.42534	0.00195	0.01140
PWY-841	2	-0.70826	0.24477	27.48293	-1.21008	-0.20644	-2.89352	0.00737	0.02485
PWY-6151	2	-0.70698	0.23432	27.32815	-1.18749	-0.22646	-3.01716	0.00547	0.02115

RIBOSYN2-PWY	2	-0.70058	0.22408	27.36986	-1.16006	-0.24109	-3.12643	0.00416	0.01867
PANTOSYN-PWY	2	-0.69562	0.22427	27.37738	-1.15548	-0.23576	-3.10175	0.00443	0.01908
PWY-4242	2	-0.68463	0.21200	27.34386	-1.11936	-0.24990	-3.22937	0.00322	0.01570
PWY66-400	2	-0.68190	0.21571	27.49036	-1.12413	-0.23966	-3.16116	0.00381	0.01758
HSERMETANA-PWY	2	-0.67865	0.22738	27.37656	-1.14489	-0.21241	-2.98468	0.00591	0.02220
PWY-7221	2	-0.67754	0.21090	27.41583	-1.10996	-0.24512	-3.21267	0.00335	0.01592
PANTO-PWY	2	-0.67682	0.23744	27.39227	-1.16368	-0.18996	-2.85047	0.00820	0.02697
1CMET2-PWY	2	-0.66698	0.21926	27.34296	-1.11661	-0.21735	-3.04191	0.00514	0.02042
PWY-2942	2	-0.66237	0.21949	27.40825	-1.11241	-0.21233	-3.01776	0.00545	0.02115
PWY-6936	2	-0.65999	0.21887	27.41727	-1.10876	-0.21122	-3.01542	0.00548	0.02115
PWY-7663	2	-0.65373	0.21066	27.47898	-1.08561	-0.22185	-3.10331	0.00440	0.01908
ANAGLYCOLYSIS-PWY	2	-0.65178	0.19983	27.49036	-1.06146	-0.24211	-3.26169	0.00295	0.01509
PWY-5973	2	-0.65171	0.20983	27.46270	-1.08191	-0.22151	-3.10587	0.00437	0.01908
PWY-5695	2	-0.64714	0.24718	27.43712	-1.15394	-0.14035	-2.61810	0.01422	0.03717
PWY-6385	2	-0.64265	0.20780	27.41454	-1.06873	-0.21658	-3.09262	0.00453	0.01908
PWY-6386	2	-0.63587	0.20708	27.41079	-1.06046	-0.21128	-3.07070	0.00478	0.01940
THISYN-PWY	2	-0.63534	0.24199	27.49036	-1.13145	-0.13922	-2.62543	0.01397	0.03717
PWY0-1261	2	-0.63176	0.20197	27.49036	-1.04582	-0.21770	-3.12800	0.00414	0.01867
PWY-6892	2	-0.63060	0.22926	27.47070	-1.10063	-0.16058	-2.75062	0.01040	0.03139
COA-PWY-1	2	-0.62330	0.21445	27.38610	-1.06303	-0.18357	-2.90650	0.00716	0.02485
PWY-724	2	-0.61931	0.20881	27.45007	-1.04742	-0.19121	-2.96599	0.00618	0.02276
PEPTIDOGLYCANSYN-PWY	2	-0.61882	0.20663	27.38024	-1.04252	-0.19513	-2.99484	0.00577	0.02182
COMPLETE-ARO-PWY	2	-0.60749	0.21981	27.43472	-1.05817	-0.15681	-2.76368	0.01009	0.03099
PWY-6387	2	-0.60266	0.20538	27.38481	-1.02379	-0.18154	-2.93441	0.00669	0.02369
ASPASN-PWY	2	-0.59305	0.24193	27.48872	-1.08904	-0.09706	-2.45129	0.02086	0.04895
PWY-6163	2	-0.58908	0.21673	27.37660	-1.03348	-0.14467	-2.71804	0.01126	0.03260
PYRIDNUCSYN-PWY	2	-0.58498	0.23641	27.49036	-1.06965	-0.10030	-2.47439	0.01980	0.04684
DENOVOPURINE2-PWY	2	-0.58125	0.22315	27.49036	-1.03874	-0.12376	-2.60470	0.01466	0.03792
PWY-5686	2	-0.58086	0.21491	27.36879	-1.02153	-0.14019	-2.70285	0.01167	0.03344
PWY-6123	2	-0.57950	0.22755	27.41066	-1.04608	-0.11293	-2.54666	0.01679	0.04187
PWY-6124	2	-0.57243	0.23028	27.42121	-1.04459	-0.10027	-2.48576	0.01931	0.04659
ARO-PWY	2	-0.57139	0.21539	27.40400	-1.01304	-0.12974	-2.65277	0.01312	0.03594
PWY-5097	2	-0.56626	0.21604	27.42100	-1.00921	-0.12331	-2.62113	0.01413	0.03717
PWY-7187	2	-0.56325	0.21520	27.48831	-1.00445	-0.12206	-2.61733	0.01424	0.03717
PWY-7357	2	-0.55517	0.21165	27.42116	-0.98914	-0.12121	-2.62304	0.01406	0.03717
PWY-6126	2	-0.55425	0.20735	27.47045	-0.97936	-0.12914	-2.67302	0.01250	0.03528
BRANCHED-CHAIN-AA	2	-0.54010	0.21116	27.49036	-0.97300	-0.10720	-2.55777	0.01635	0.04113
PWY-5103	2	-0.52794	0.20945	27.49036	-0.95733	-0.09855	-2.52066	0.01781	0.04362
PWY-6545	2	-0.26621	0.25065	27.42389	-0.78012	0.24771	-1.06207	0.29747	0.39192
PWY0-162	2	0.20321	0.24604	27.31545	-0.30135	0.70778	0.82592	0.41600	0.51286
GALACTUROCAT-PWY	2	0.35054	0.23600	27.39853	-0.13337	0.83445	1.48533	0.14887	0.23502
PPGPPMET-PWY	2	0.63071	0.61355	27.45618	-0.62721	1.88863	1.02798	0.31293	0.40843
PWY-5676	2	0.91926	0.26988	27.47053	0.36596	1.47256	3.40620	0.00204	0.01172
PWY-6305	2	1.20765	0.30791	27.49036	0.57640	1.83890	3.92210	0.00053	0.00492
PWY-6834	2	3.04232	1.32767	27.34621	0.31977	5.76487	2.29147	0.02986	0.06650
PWY-5367	2	3.33441	1.31248	27.41453	0.64333	6.02549	2.54055	0.01703	0.04192

PWY-6284	2	3.94870	1.44508	27.41938	0.98576	6.91165	2.73251	0.01087	0.03180
KETOGLUCONMET-PWY	2	4.20699	1.30886	27.29378	1.52278	6.89120	3.21424	0.00335	0.01592
PWY-7090	2	4.32515	1.40509	27.49036	1.44455	7.20575	3.07821	0.00468	0.01940
PWY-6113	2	4.58111	1.59113	27.41375	1.31868	7.84355	2.87915	0.00765	0.02545
PWY-6588	2	4.64991	1.38119	27.48610	1.81828	7.48155	3.36659	0.00226	0.01270
PWY-5971	2	4.91005	1.66073	27.40959	1.50489	8.31520	2.95656	0.00633	0.02301
PWY-1861	2	5.06358	1.55677	27.30424	1.87101	8.25614	3.25261	0.00304	0.01509
