# Supplementary material for: Fecal microbiota transfer between young and aged mice reverses hallmarks of the aging gut, eye, and brain
Source: Microbiome. 2022 Apr 29;10:68. doi: 10.1186/s40168-022-01243-w (PMC9063061; doi:10.1186/s40168-022-01243-w)
Supplement: Supplementary file 6 — Additional file 5: Figure S1. Related to Fig. 2. Iba-1+ cell density in corpus callosum is regulated by the intestinal microbiota. (A) Iba-1+ microglia were identified by immunostaining (red), nuclei counterstained with Hoechst (blue) in the corpus callosum (highlighted in cartoon) of sagittal brain sections. (B) Representative immunostaining of young, old, and aged mice, either treated with PBS only, treated with antibiotics (Abx) only, or with antibiotics followed by FMT from young, old, or aged donors. Quantified in Fig. 2. Figure S2. Related to Fig. 5. Impact of antibiotics and microbiota transfers on observed species. Number of observed bacterial species pre– and post– antibiotic treatment, post–FMT and at the end of the experiment. Pairwise comparison, Kruskal–Wallis, * = P < .0001. Figure S3. Related to Fig. 6. Clustering of beta–diversity post–FMT is driven by donor age, and differential abundance of bacterial families identified pre–and post–transfer. (A) Differential abundance of bacterial families in aged mice receiving young donor microbiota, and in young mice receiving young, old, or aged donor microbiota. (B) Species enriched in the young mice receiving young donor microbiota. (C) Microbiota surviving/enriched post–antibiotics only. Figure S4. Metabolite profiles in fecal pellets estimated by (1 H)–NMR. (A) PLS–DA comparing fecal metabolite profiles (full list of metabolites in Supplementary Table S3) of young and aged groups pre– and post–heterochronic transfer estimated by NMR. (B) Specific metabolites contributing to the loading of component 1, 2, and 3 in the PLS–DA analysis. Figure S5. Behavioral testing in aged mice shows no difference between aged mice receiving young vs. aged donor FMT. (A) Novel object recognition (NOR) test and (B) Y -maze test results for: young mice (n = 11), aged mice (n = 20), aged mice+ young donor FMT (Y-FMT) (n = 10) and aged mice + aged donor FMT (A-FMT) (n = 10). Error bars denote 95% CI. Figure S6. Per-sample read n [file 40168_2022_1243_MOESM6_ESM.pdf]

Figure S1.

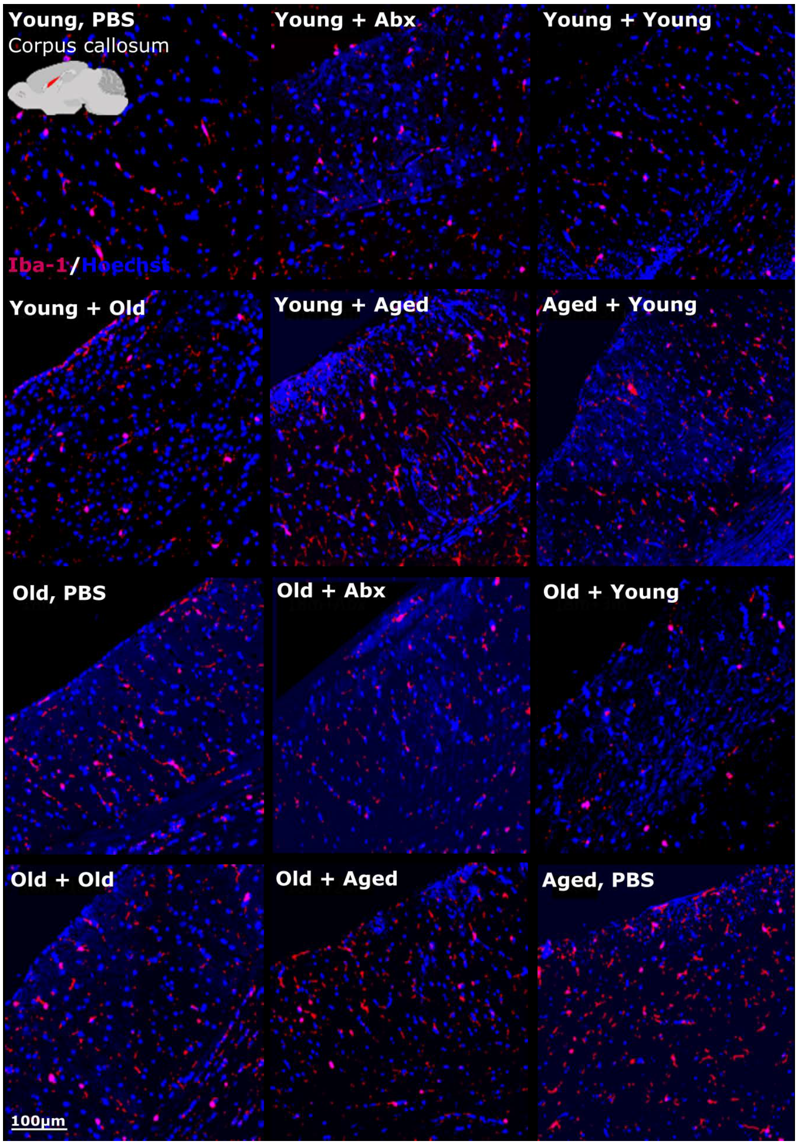

Figure S1. Related to Figure 2. Iba-1^{+} cell density in corpus callosum is regulated by the intestinal microbiota. (**A**) Iba-1^{+} microglia were identified by immunostaining (red), nuclei counterstained with Hoechst (blue) in the corpus callosum (highlighted in cartoon) of sagittal brain sections. (**B**) Representative immunostaining of young, old, and aged mice, either untreated, treated with antibiotics (Abx) only, or with antibiotics followed by FMT from young, old, or aged donors. Quantified in Figure 2.

Figure S2.

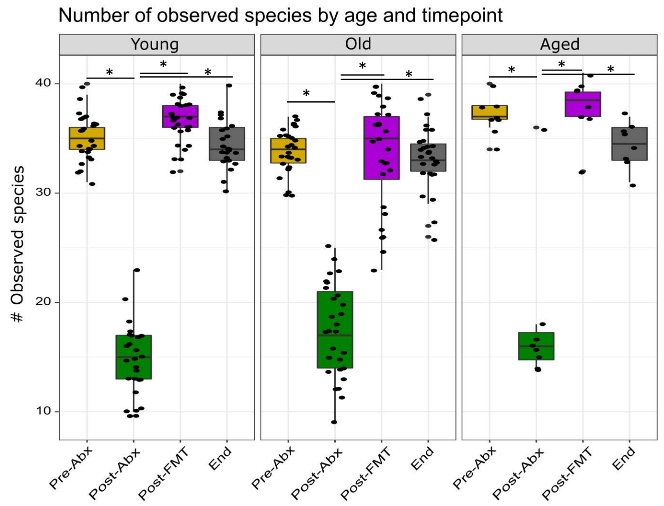

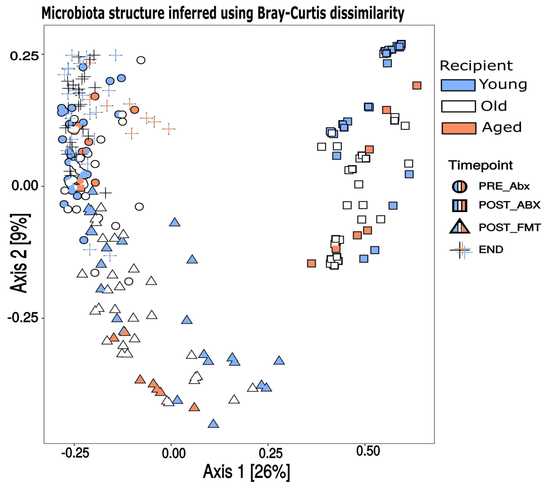

Figure S2. Related to Figure 5. Impact of antibiotics and microbiota transfers on number of observed species. (**A**) Number of observed bacterial species pre- and post- antibiotic treatment, post– FMT and at the end of the experiment. Pairwise comparison, Tukey post-hoc tests, *= P<.0001. (**B**) PCoA (Bray–Curtis) depicting clustering of all mice from all age and treatment groups, colored by age group, Young in blue markers, Old in white/black markers, Aged in orange markers.

Figure S3.

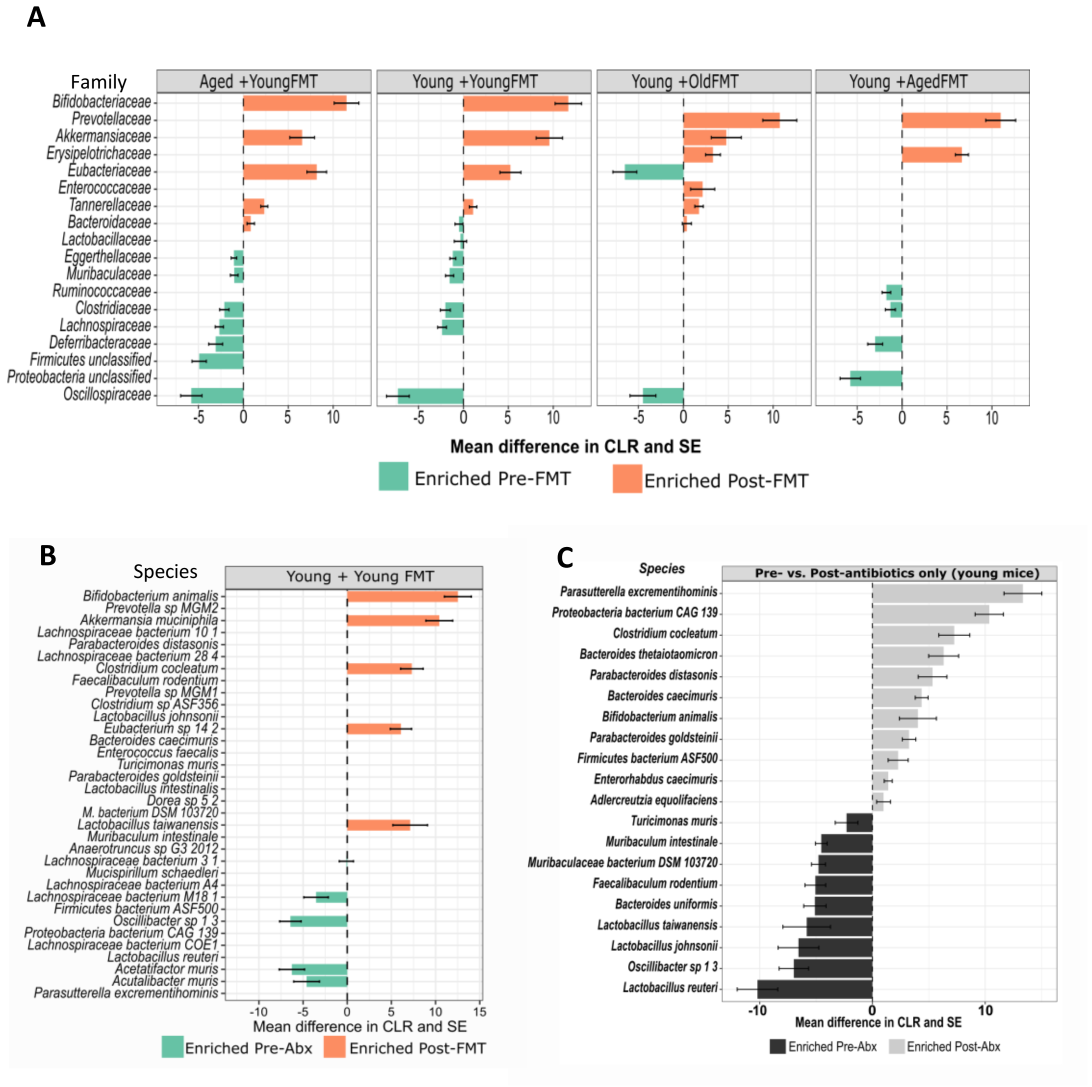

Figure S3. Related to Figure 6. Clustering of beta–diversity post–FMT is driven by donor age, and differential abundance of bacterial families identified pre–and post–transfer. (A) Differential abundance of bacterial families in aged mice receiving young donor microbiota, and in young mice receiving young, old, or aged donor microbiota. (B) Species enriched in the young mice receiving young donor microbiota. (C) Microbiota surviving/enriched post–antibiotics only.

Figure S4

A

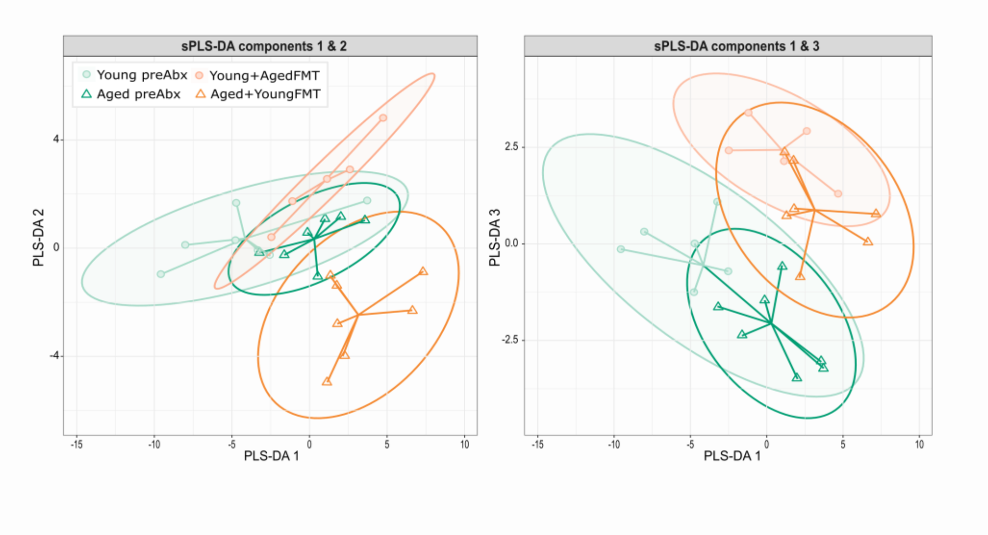

B

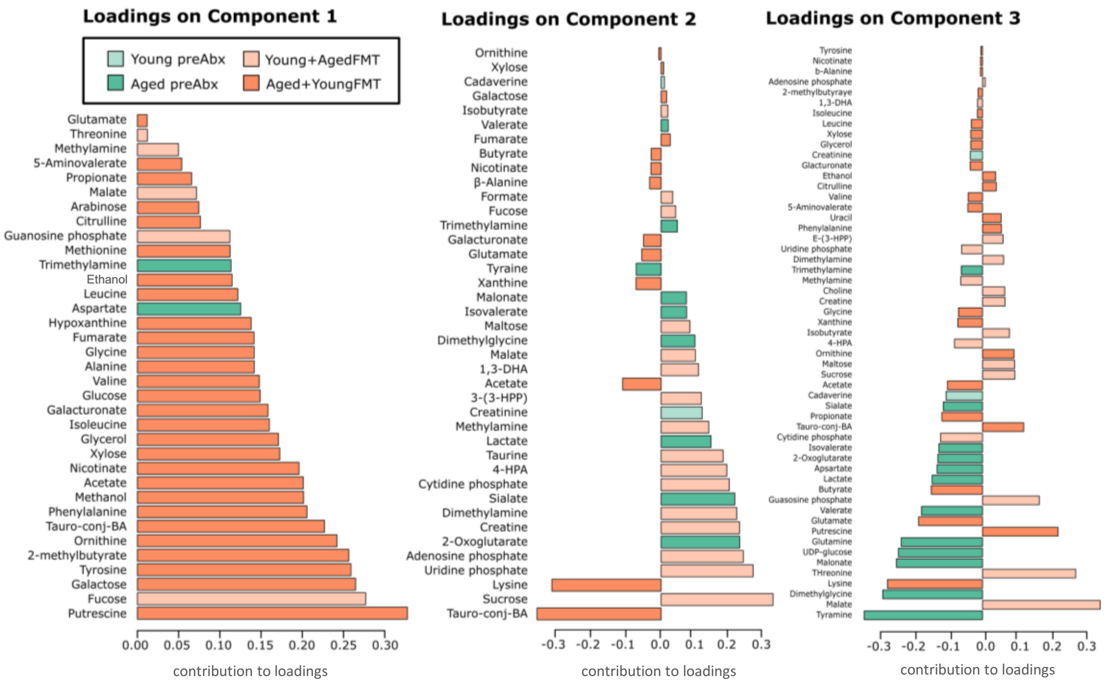

Figure S4. Metabolite profiles in fecal pellets estimated by (1 H)–NMR.
(A) PLS–DA comparing fecal metabolite profiles (full list of metabolites in Supplementary Table S3) of young and aged groups pre– and post–heterochronic transfer estimated by NMR. (B) Specific metabolites contributing to the loading of component 1, 2, and 3 in the PLS–DA analysis.

Figure S5

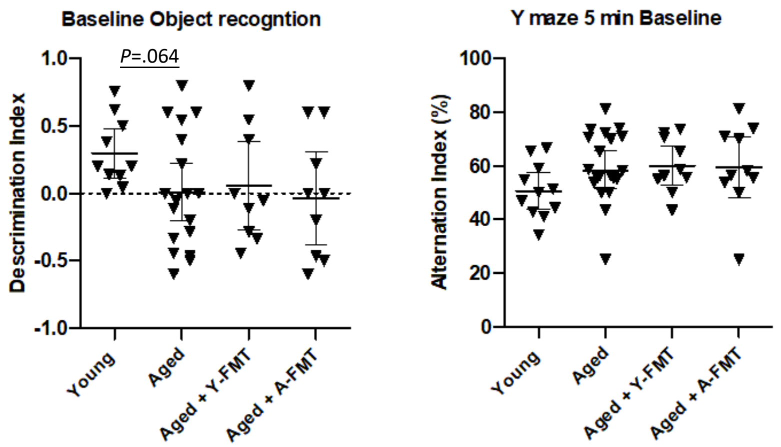

Figure S5. Behavioral testing in aged mice shows no difference between aged mice receiving young vs. aged donor FMT. (A) Novel object recognition (NOR) test and (B) Y -maze test results for: young mice (n = 11), aged mice (n = 20), aged mice+ young donor FMT (Y-FMT) (n = 10) and aged mice + aged donor FMT (A-FMT) (n = 10). Error bars denote 95% CI.

Figure S6

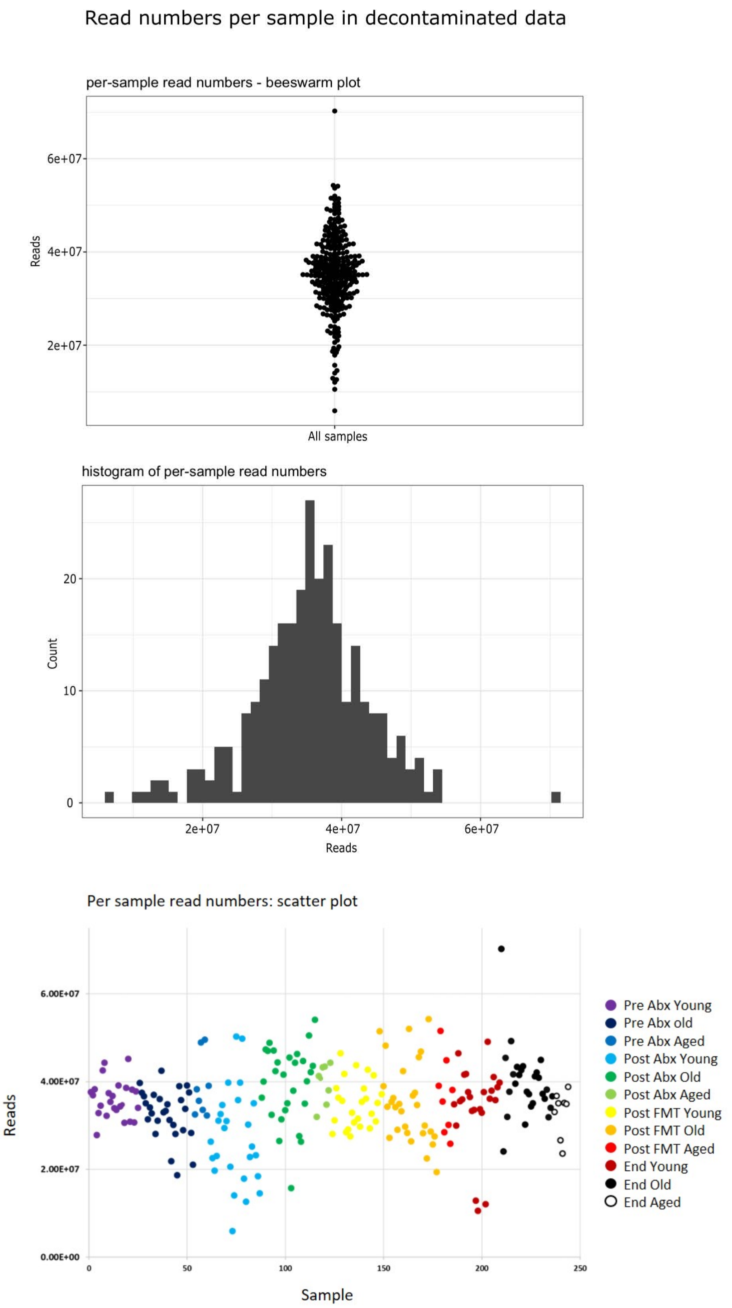

Figure S6. Per-sample read numbers in metagenomic sequencing data.
(**A**) Beeswarm plot, (**B**) histogram, and (**C**) scatter plot, depicting per-sample read numbers in trimmed and decontaminated metagenomic sequencing data. Samples from all mice from all groups.

Figure S7

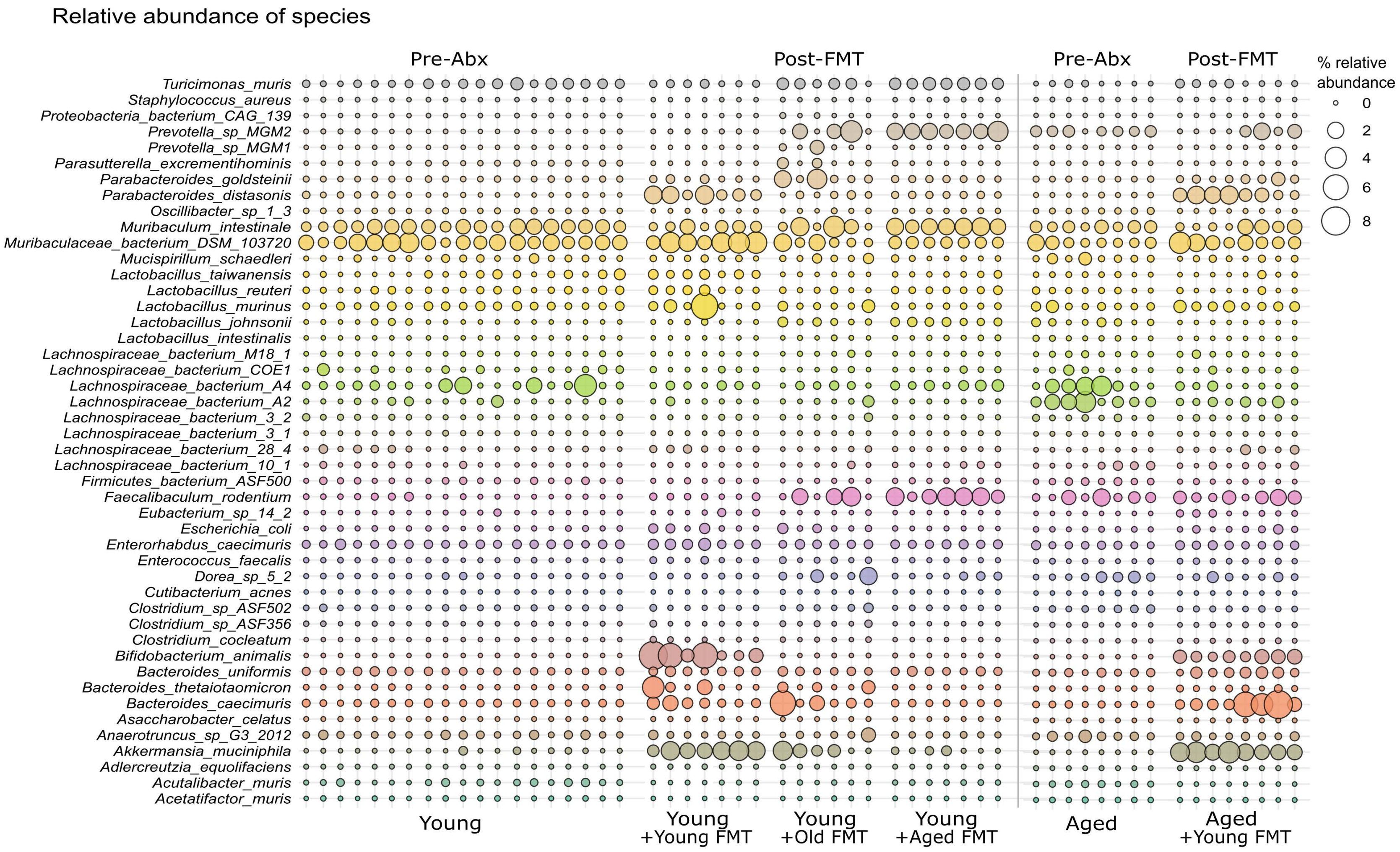

Figure S7. Bubble plot depicting variation between metagenomic samples across mice within young and aged groups pre- and post-FMT.

Figure S8

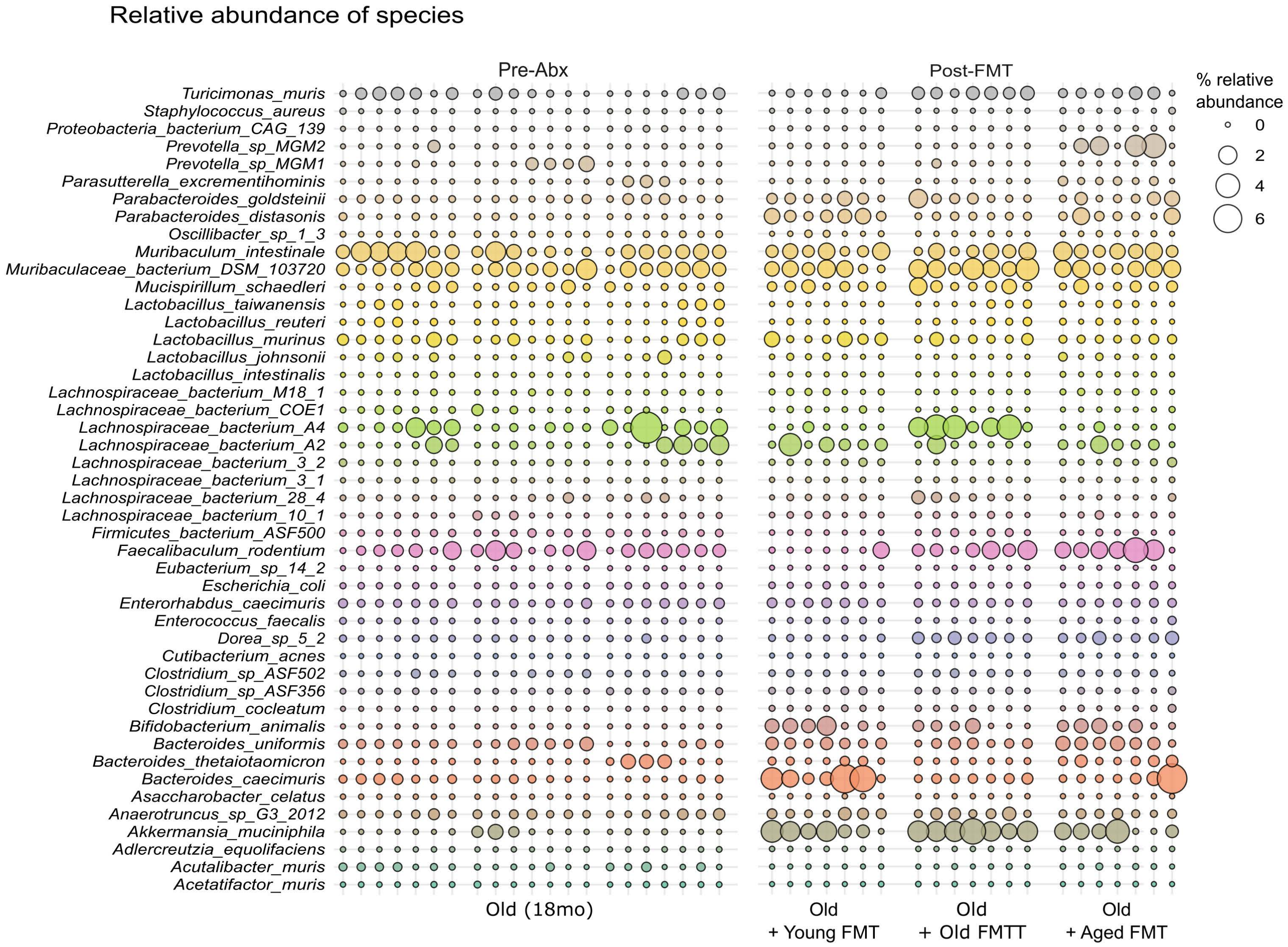

Figure S8. Bubble plot depicting variation between metagenomic samples across mice within old groups pre- and post-FMT.
