# Supplementary material for: Fecal microbiota transfer between young and aged mice reverses hallmarks of the aging gut, eye, and brain
Source: Microbiome. 2022 Apr 29;10:68. doi: 10.1186/s40168-022-01243-w (PMC9063061; doi:10.1186/s40168-022-01243-w)
Supplement: Supplementary file 7 — Additional file 6. Key Resources Table. [file 40168_2022_1243_MOESM7_ESM.docx]

| Key Resources Table |  |  |  |
| --- | --- | --- | --- |
| Mice | Supplier | Catalog number | RRID |
| C57BL/6J, bred, maintained, and aged at the UEA |  |  |  |
| Antibodies/stains/histology |  |  |  |
| Recombinant Anti-Iba1 antibody [EPR16588] | Abcam | ab178846 | AB_2636859 |
| Anti-Retinal Pigment Epithelium 65 Antibody | Merck | MAB5428 | AB_571111 |
| Anti-mouse Complement C3 goat antiserum | MP Biomedicals | 855444 |  |
| Donkey anti-Goat IgG (H+L) Cross-Adsorbed A568 | Invitrogen | A-11057 | AB_142581 |
| Alexa Fluor 594 Donkey anti-rabbit IgG | Biolegend | 406418 | AB_2563306 |
| Donkey anti-Goat IgG (H+L) Cross-Adsorbed A488 | Invitrogen | A11055 | AB_2534102 |
| Donkey anti-Mouse IgG (H+L) Cross-Adsorbed A568 | Invitrogen | A10037 |  |
| Sheep anti-BrdU-Biotin | Abcam | ab1893 | AB_302659 |
| Neutravidin-HRP | Pierce | 31032 |  |
| DAB kit | Vector Labs | SK-4100 |  |
| NucBlue Live ReadyProbes Reagent (Hoechst 33342) | Invitrogen | R37605 |  |
| DAPI | Sigma | D9542 |  |
| Tissue RNA/cDNA/qPCR |  |  |  |
| NucleoSpin RNA II kit | Machery-Nagel | 740955 |  |
| GoSCRIPT REVERSE TRANSCRIPTION SYSTEM | Promega | A5001 |  |
| Kicqstart SYBR mix | Sigma | 204054 |  |
| Facecal gDNA isolation, library preparation and WGS |  |  |  |
| FastDNA SPIN Kit for Soil | MP Biomedicals | 11492400 |  |
| Qubit dsDNA BR Assay Kit | Invitrogen | Q32850 |  |
| Nextera DNA Flex Library Prep Kit | Illumina | 20018704 |  |
| Kap2G Robust PCR kit | Sigma | KK5005 |  |
| Nextera XT Index Kit v2 index primers | Illumina | FC-131-2001 to 2004 |  |
| Quant-iT dsDNA Assay Kit | Invitrogen | 10164582/Q33120 |  |
| KAPA Pure Beads | Roche | 7983298001 |  |
| D5000 ScreenTape system | Agilent | 5067- 5588 & 5067- 5589 |  |
| Antibiotics |  |  |  |
| Ampicillin, Neomycin, Metranidzole, Vancomycin | Sigma | A9393, N6386, M1547, V0045000 | |
| ELISA |  |  |  |
| LBP, Mouse, ELISA kit | Cam Bioscience Ltd | HK205-01 |  |
| Serotonin ELISA Kit | Abcam | ab133053 |  |
| Mouse TNF-alpha Quantikine HS ELISA Kit | Bio-Techne Ltd | MHSTA50 |  |
| Luminex |  |  |  |
| Mouse Magnetic Luminex Assay | Bio-Techne Ltd | LXSAMSM |  |
| NMR |  |  |  |
| Sodium 3-trimethysilyl-propionate-d4 (TSP-d4) | Sigma | 269913 |  |
| Deuterium oxide (D_2_O) | Sigma | 450510 |  |
| Hormone release assay |  |  |  |
| Moclobemide | Sigma | M3071 |  |
| Fluoxetine | Sigma | F132 |  |
| Proilferation assay |  |  |  |
| 5-Bromo-2′-deoxyuridine | Sigma | B5002 |  |
| Software |  |  |  |
| FIJI/ImageJ v1.52n | https://fiji.sc/ |  |  |
| Jamovi (v1.1.9.0) | https://www.jamovi.org/ |  |  |
| GraphPad Prism v5.04 | https://www.graphpad.com/scientific-software/prism/ | | |
| Zeiss Zen 2010 | hzeiss.com/microscopy/int/products/microscope-software/zen.html | | |
| R packages as detailed in Methods. |  | | |
